# TGFβ1-Induced EMT in the MCF10A Mammary Epithelial Cell Line Model Is Executed Independently of SNAIL1 and ZEB1 but Relies on JUNB-Coordinated Transcriptional Regulation

**DOI:** 10.3390/cancers15020558

**Published:** 2023-01-16

**Authors:** Pablo Antón-García, Elham Bavafaye Haghighi, Katja Rose, Georg Vladimirov, Melanie Boerries, Andreas Hecht

**Affiliations:** 1 Institute of Molecular Medicine and Cell Research, Faculty of Medicine, University of Freiburg, 79104 Freiburg, Germany; pablo.anton.garcia@mol-med.uni-freiburg.de (P.A.-G.); katja.rose@mol-med.uni-freiburg.de (K.R.); georgvladimirov@web.de (G.V.); 2 Faculty of Biology, University of Freiburg, 79104 Freiburg, Germany; 3 Institute of Medical Bioinformatics and Systems Medicine, Medical Center–University of Freiburg, Faculty of Medicine, University of Freiburg, 79106 Freiburg, Germany; elham.bavafaye.haghighi@uniklinik-freiburg.de (E.B.H.); melanie.boerries@uniklinik-freiburg.de (M.B.); 4 German Cancer Consortium (DKTK), Partner Site Freiburg, German Cancer Research Center (DKFZ), 69120 Heidelberg, Germany; 5 BIOSS Centre for Biological Signalling Studies, University of Freiburg, 79104 Freiburg, Germany

**Keywords:** epithelial-mesenchymal transition, invasion, metastasis, breast cancer, SNAIL1, ZEB1, JUNB, chromatin accessibility, TGFβ signaling, transcriptional regulation

## Abstract

**Simple Summary:**

While invading tumor-adjacent tissue, cancer cells often undergo epithelial-mesenchymal transition (EMT). A mechanistic understanding of EMT may therefore improve diagnostic and therapeutic options. Traditionally, EMT is thought to be regulated by SNAIL, TWIST, and ZEB transcription factors. Since this view is increasingly challenged, we aimed to examine the importance of traditional EMT regulators while identifying alternative factors potentially orchestrating EMT. By combining computational analyses of epigenomic and transcriptomic data with loss-of-function experiments, we demonstrate that JUNB, a component of the AP-1 transcription factor complex, is crucial for EMT in the MCF10A mammary epithelial cell line model, but not SNAIL proteins and ZEB1. We exploited the JUNB-dependence of EMT to define a gene signature which is controlled by TGFβ in multiple cancer entities and which is predictive of patient survival. Our results provide a more refined picture of the dynamic regulation of EMT and suggest that traditional models for EMT regulation may need to be revised.

**Abstract:**

Epithelial-mesenchymal transition (EMT) fosters cancer cell invasion and metastasis, the main cause of cancer-related mortality. Growing evidence that SNAIL and ZEB transcription factors, typically portrayed as master regulators of EMT, may be dispensable for this process, led us to re-investigate its mechanistic underpinnings. For this, we used an unbiased computational approach that integrated time-resolved analyses of chromatin structure and differential gene expression, to predict transcriptional regulators of TGFβ1-inducible EMT in the MCF10A mammary epithelial cell line model. Bioinformatic analyses indicated comparatively minor contributions of SNAIL proteins and ZEB1 to TGFβ1-induced EMT, whereas the AP-1 subunit JUNB was anticipated to have a much larger impact. CRISPR/Cas9-mediated loss-of-function studies confirmed that TGFβ1-induced EMT proceeded independently of SNAIL proteins and ZEB1. In contrast, JUNB was necessary and sufficient for EMT in MCF10A cells, but not in A549 lung cancer cells, indicating cell-type-specificity of JUNB EMT-regulatory capacity. Nonetheless, the JUNB-dependence of EMT-associated transcriptional reprogramming in MCF10A cells allowed to define a gene expression signature which was regulated by TGFβ1 in diverse cellular backgrounds, showed positively correlated expression with TGFβ signaling in multiple cancer transcriptomes, and was predictive of patient survival in several cancer types. Altogether, our findings provide novel mechanistic insights into the context-dependent control of TGFβ1-driven EMT and thereby may lead to improved diagnostic and therapeutic options.

## 1. Introduction

The progression of solid tumors to metastatic disease accounts for most cancer-related deaths and remains a significant challenge in cancer treatment [1]. A cell biological process that empowers cancer cells with metastases-forming abilities, is the epithelial-mesenchymal transition (EMT) [2]. EMT represents reversible transdifferentiation events that occur naturally during embryonic development or wound healing but appear to be hijacked by cancer cells in the course of carcinogenesis [2]. When undergoing EMT, highly proliferative and immobile epithelial cells lose their apical-basal polarity, tight intercellular contacts, and interaction with the basal membrane. Instead, the cells gain mesenchymal properties including fibroblast-like shapes, increased migratory and invasive capacities, and become more therapy-resistant [2]. The replacement of epithelial by mesenchymal features is a gradual and reversible process, reflected by the existence of a broad and continuous spectrum of hybrid or partial EMT states which are characterized by the co-expression of epithelial and mesenchymal traits at variable ratios [3,4,5].

In the majority of cases, EMT processes are based on transcriptional reprogramming and encompass massive changes in gene expression including the repression of epithelial marker genes such as *CDH1* (encoding E-CADHERIN) [2,6]. However, a particular type of partial EMT that entailed the loss of epithelial traits based on internalization of E-CADHERIN rather than its transcriptional downregulation, was also reported [7]. Multiple conditions and external signals can induce EMT, including transforming growth factor β (TGFβ) signaling as a prominent and well-studied example [2,5,8]. Molecular models describing how EMT-associated transcriptional changes are orchestrated downstream of TGFβ signaling and other EMT inducers, commonly assign central roles in this process to a small cohort of transcription factors (TFs) from the SNAIL, ZEB, and TWIST families which are presumed to be EMT master regulators [3,9]. A shared property of these EMT TFs is the ability to directly bind to so-called E-box DNA motifs, thereby frequently, but not always leading to the repression of their target genes [9,10,11,12,13,14]. However, an increasing number of publications provide evidence that a multiplicity of molecularly distinct and context-dependent EMT programs may exist [6,7,8,15,16,17] and that at least some of these can proceed in the absence for example of SNAIL and ZEB TFs [6,7,17,18,19]. This challenges traditional views of the molecular underpinnings of transcriptionally regulated EMT programs and raises the question which other TFs aside from SNAIL, TWIST, and ZEB family members might play a role in their execution. 

Experimental strategies to identify candidate regulators of gene expression programs take advantage of structural hallmarks of cis-regulatory DNA sequences such as promoters and enhancers [20,21]. These DNA sequences represent clusters of TF binding motifs (TFBMs) and thus facilitate site-specific assembly of multifactorial protein complexes formed by TFs and their co-regulators [20]. Thereby, promoters and enhancers control the reorganization of chromatin structure and transcriptional activation or repression of associated genes. Promoters and enhancers can exist in alternative functional states which exhibit characteristic differences. Thus, inactive cis-regulatory elements for instance are occupied by nucleosomes, display low DNA accessibility, and feature absence of TF binding. In contrast, active promoters and enhancers are nucleosome-free, exhibit increased DNA accessibility, and are bound by TFs [20]. Consequently, by probing for open and closed chromatin with accessible and inaccessible DNA sequences, respectively, for example by employing the assay for transposase-accessible chromatin using sequencing (ATAC-seq), one can determine the location of candidate regulatory elements and changes in their activity under different experimental conditions [21]. Furthermore, by examining the sequence composition of candidate regulatory elements with respect to the occurrence of TFBMs, and by additionally consulting curated databases with collections of potential target genes for a given TF, the integrated analyses of ATAC-seq and RNA-seq data allows for high confidence prediction of TFs likely to govern a biological process [22,23]. 

The increasing number of reports calling into question a central and obligatory role for TFs commonly considered to be master regulators of EMT, prompted us to re-examine the regulatory landscape of TGFβ1-induced EMT in the widely used human MCF10A mammary epithelial cell line model. By analyzing in a time-resolved manner TGFβ1-induced changes in chromatin structure and gene expression, and by conducting integrative analysis of motif activity and gene expression changes of transcription factors (IMAGE) [23] we obtained evidence that SNAIL proteins and ZEB1 may play only subordinate roles in the MCF10A EMT model system. This was experimentally confirmed by individual knockout of the *SNAI1*, *SNAI2*, and *ZEB1* genes and by combinatorial inactivation of *SNAI1* and *ZEB1*, none of which had a discernible impact on TGFβ1-induced EMT of the mammary epithelial cell line. In contrast, guided by IMAGE, we confirmed that JUNB, one of the TFs recognizing the AP-1 TFBM, is a critical regulator of TGFβ1-induced EMT in MCF10A cells. Even though its functional importance in EMT turned out to vary in a cell-type-specific fashion, we exploited the JUNB-dependence of TGFβ1-induced EMT in MCF10A cells to define a set of high confidence direct JUNB target genes which were activated in response to TGFβ1 in several cell lines originating from different tissues. Moreover, expression of this gene set and TGFβ1 pathway activity were correlated in various tumor transcriptomes and turned out to be predictive of poor prognosis in several tumor entities. Altogether, our findings provide new mechanistic insights into the molecular machinery of TGFβ1-induced EMT and provide valuable information to better identify cancer cells undergoing EMT in a clinical context.

## 2. Materials and Methods

### 2.1. Cell Lines, Oligonucleotides, and Antibodies

Cell lines that were generated and used in this study, their origin, and their culture conditions are listed in Table S1. Oligonucleotides used as primers for qRT-PCR and chromatin immunoprecipitation followed by qPCR (ChIP-qPCR) are presented in Table S2. Antibodies utilized in immunoblotting, immunofluorescence, and ChIP experiments, as well as their corresponding dilutions, can be found in Table S3.

### 2.2. CRISPR/Cas9-Mediated Genome Editing

Single guide RNAs (sgRNAs) were designed and selected using the online tool Benchling (https://benchling.com/ (accessed several times from 15 May 2018 to 2 July 2021)). For expression of the sgRNAs, suitable oligonucleotides were cloned into the plasmid pMuLE_ENTR_U6_stuffer_sgRNA_scaffold_R3-R4 [24]. For genome editing, a total number of 6 × 10^6^ MCF10A cells were transfected with 3 μg of each sgRNA construct, and 4 μg of the Cas9-turboRFP expression vector [25] using the Cell Line Nucleofector kit L (#VCA-1005, Lonza, Basel, Switzerland) in a Nucleofector 2b device (#AAB-1001, Lonza), or Lipofectamine LTX with Plus Reagent (#A12621, Thermo Fisher Scientific, Dreieich, Germany). RFP^+^ cells were single-cell sorted into 96-well plates 72 h post transfection, expanded, and screened for successful genome editing by PCR. Details about the genomic state of the targeted loci for all cell clones can be found in Table S4. A list of sgRNA target sequences used for all genome editing strategies in this study is presented in Table S5.

### 2.3. Generation of Cell Lines Stably Expressing JUNB-ER Fusion Proteins

For constitutive expression of JUNB-ER fusion proteins in MCF10A and A549 cells, the human *JUNB* coding sequences flanked by extensions with BamHI restriction enzyme sites were PCR-amplified from genomic DNA of MCF10A cells using the Q5 high fidelity polymerase (M0491, New England Biolabs GmbH, Frankfurt, Germany). After digestion with BamHI, the JUNB PCR fragment was inserted in sense and antisense orientations into the retroviral vector pWZL-Blast-SNAIL1-ER (addgene plasmid #18798; http://n2t.net/addgene:18798 (accessed on 28 August 2022); RRID: addgene_18798) [26], whereby the SNAIL1 coding sequences were deleted from the vector. Importantly, the estrogen receptor (ER) hormone binding domain in this construct carries a G525R amino acid exchange which renders the mutant ER domain insensitive towards 17-β-estradiol and allows selective activation by 4-hydroxytamoxifen (4-OHT) [27]. Recipient cell lines were transduced with the *JUNB* sense and antisense constructs and were selected by addition of 6 µg/ml blasticidin to cell culture media as previously described [28]. JUNB-ER activity was induced by treating cells with 100 nM 4-OHT (H7904, Sigma-Aldrich, Taufkirchen, Germany), while solvent control samples received a corresponding volume of ethanol (EtOH). Both reagents were refreshed every 48 h. 

### 2.4. TGFβ1 Treatment of Cells

In every experiment that involved TGFβ1 stimulation, equal cell numbers were seeded for each experimental condition. At 24 h post-seeding, cells received recombinant human TGFβ1 (#100-21, PeproTech, Rocky Hill, NJ, USA) at a final concentration of 5 ng/mL, or a corresponding volume of solvent solution containing 0.1% BSA in PBS. TGFβ1 and solvent were refreshed every 48 h for longer treatment experiments.

### 2.5. Protein Isolation and Western Blotting

In order to prepare protein extracts for Western blotting, 1 × 10^6^ cells were seeded in 10 cm dishes and treated as described in the figure legends. Cells were scraped off the dishes and washed with ice-cold PBS. Nuclear and cytoplasmic protein fractions were prepared as previously described [29]. Cytoplasmic fractions were used to detect N-Cadherin, EpCAM, Fibronectin, RBM47, and Beta-actin. Nuclear fractions were used to detect JUN, JUNB, SNAIL1, SNAIL2, ZEB1, and GSK-3 beta. Protein concentrations of cell lysates were determined with the BioRad DC™ Protein Assay (#500-0113, BioRad, Feldkirchen, Germany). From each sample, aliquots with 20 μg of protein content were separated by SDS-polyacrylamide gel electrophoresis, except for the immunodetection of JUN and JUNB, for which 5 μg of protein were used. After electrophoresis, proteins were transferred onto nitrocellulose membranes for detection as previously described [29].

### 2.6. Phase Contrast and Fluorescence Microscopy

In order to document cellular morphology, phase contrast images were taken using a 10× objective of a Nikon Eclipse TS100 microscope equipped with a DS-L3 digital camera (Nikon, Minato, Tokyo, Japan). To detect expression and intracellular localization of proteins by immunofluorescence, 2.5 × 10^4^ cells were seeded on glass coverslips coated with 0.1% gelatin and placed in 24-well plates. After attachment, cells were treated with TGFβ1 or solvent for 72 h. Thereafter, cells were fixed with 4% formaldehyde (#28908, Thermo Fisher Scientific, Dreieich, Germany), and processed for immunofluorescence staining as previously described [30]. F-actin was stained using phalloidin coupled to Alexa Fluor-555 (#8953S, Cell Signaling Technologies, Danvers, MA, USA). Images were taken with a Zeiss Axio Observer Z1 fluorescence microscope equipped with an ApoTome2 device (Zeiss, Oberkochen, Germany). Images were taken using the 40× objective with immersion oil. If necessary, brightness and contrast were adjusted for all images of an experimental series in an identical way using the Canvas™ X 2017 software (Plantation, FL, USA). 

### 2.7. RNA Isolation and Targeted Gene Expression Analysis by qRT-PCR

In order to prepare total RNA using the PeqGOLD total RNA kit (#732–2871, Peqlab/VWR Life Science, Bruchsal, Germany), 1 × 10^5^ cells were seeded per well of a 6-well plate and treated as described in the figure legends. Subsequently, cDNA was synthesized with the qScript Flex cDNA Kit (#95049, Quantabio, Beverly, MA, USA) and used as template for qRT-PCR using the PerfeCTa SYBR Green SuperMix (#95049, Quantabio) and the CFX96 Touch Real-Time PCR Detection System (Bio-Rad Laboratories, Hercules, CA, USA). Relative gene expression levels were calculated by normalizing transcript levels of the genes under investigation to those of *GAPDH*, using the 2^−ΔCt^ method.

### 2.8. Population Dynamics and Cell Cycle Analysis

In order to monitor changes in population dynamics and cell cycle progression, 1 × 10^5^ cells were seeded in 6 cm dishes and treated with TGFβ1 or solvent for 72 h. At the end of the treatment, cells were trypsinized. Trypsin was inactivated by adding advanced DMEM/F12 medium containing 5% horse serum, finally generating a single cell suspension with a total volume of 1 ml. From this cell suspension, 10 µL were used to manually determine cell counts with a hemocytometer. The remaining part of the cell suspension was transferred to FACS tubes and processed for cell cycle analysis as previously published [25]. Flow cytometry data were acquired and analyzed using the CytoFLEX S machine and the CytExpert software for the CytoFLEX platform (B78557, Beckman Coulter, Krefeld, Germany).

### 2.9. Transwell Migration Assays

For each cell line or cell clone to be analyzed, 2 wells of a 6-well plate were seeded with 1 × 10^5^ cells per well. Cells were pre-treated with TGFβ1, 4-OHT, or the corresponding solvent controls for 48 h. Thereafter, cells were detached with trypsin, counted, and transferred to the upper chambers of transwell membrane inserts with 8 µm pore size (#353097; Corning, Corning, NY, USA). For MCF10A cells and their derivatives, 7.5 × 10^4^ cells/sample were seeded in advance DMEM/F12 medium without supplements. Complete MCF10A growth medium was added to the lower chambers. For A549 cells and their derivatives, 1.5 × 10^4^ cells/sample were seeded in DMEM medium without supplements. Complete A549 growth medium was added to the lower chambers. Both upper and lower chambers received TGFβ1, 4-OHT, or the corresponding solvent controls. Cells were incubated for 24 h after which cells that had migrated through the membrane, were stained with crystal violet and detected with a BZ-9000 microscope (Keyence Deutschland GmbH, Neu Isenburg, Germany) as previously described [31]. Quantification was carried out using ImageJ. First, a binary image was created and subsequently the integrated area of all signals was divided by the total area of the transwell to calculate the percentage of covered area by migrating cells [17,31].

### 2.10. Spheroid Invasion Assays

Cells were seeded and pretreated as described as for the transwell migration assays. Thereafter, cells were detached with trypsin, counted, and spheroids were formed using the hanging drop method as previously described [28]. For MCF10A cells and their derivatives, 1500 cells per hanging drop were used. For A549 cells and their derivatives, 750 cells per hanging drop were used. Spheroids were allowed to form in hanging drops in the presence of TGFβ1, 4-OHT, or the corresponding solvent controls for 24 h. Thereafter, spheroids were collected and embedded in matrices containing collagen I as previously described [28]. Once the collagen I matrices had solidified, the samples were overlaid with media containing TGFβ1, 4-OHT, or the corresponding solvent controls. After incubation for 72 h, pictures were taken using the 10× objective of a Nikon Eclipse TS100 microscope equipped with a DS-L3 digital camera. For quantitative evaluation, single cells and small cell aggregates that had detached from the bulk of the spheroids, were manually counted. Alternatively, the number and length of invasive protrusions extending from the spheroids were measured using ImageJ. For this, the length of the scale bar in μm was transformed into an equivalent number of pixels and set as reference. Next, the straight line selection tool of ImageJ was used to connect the center of a spheroid and the tip of each of its invasive protrusions. The lengths of all lines were recorded and used to calculate mean sprout lengths.

### 2.11. ChIP-qPCR

Prior to ChIP experiments, two 15 cm dishes were seeded with 3 × 10^6^ cells per dish and treated with TGFβ1 or solvent for 24 h. After treatment, chromatin was prepared using the truChIP chromatin shearing kit (#520154, Covaris, Woburn, MA, USA) with the following two modifications. First, nuclei were isolated following the NEXSON protocol [32], using the Bioruptor Plus sonicator device (Diagenode, Seraing, Belgium) applying 5 cycles of sonication (15 s on/30 s off) with the low amplitude setting. Second, chromatin shearing was performed using the Bioruptor Plus for 50 cycles (30 s on/30 s off) with the high amplitude setting. All probes were gently mixed every 10 cycles. The concentration of isolated chromatin was determined in a NanoDrop 2000 device (ND-2000, Thermo Fisher Scientific). JUNB immunoprecipitations were carried out using 50 µg of chromatin and Dynabeads Protein G (#10003D, Thermo Fisher Scientific) but otherwise following the protocol provided in the truChIP chromatin shearing kit. After immunoprecipitation, the subsequent washing steps, reversal of crosslinking, and qPCR analyses were carried out as previously described [30], except that DNA was purified with the ChIP DNA Clean and Concentrator kit (#D5205, Zymo Research, Irvine, CA, USA). 

### 2.12. ATAC-seq

Chromatin accessibility profiles of MCF10A cells were obtained using time-resolved ATAC-seq. For this, 1 × 10^5^ cells per well were seeded in four wells of a 6-well plate and treated with TGFβ1 for 6, 24, and 72 h, with one well receiving solvent as control. From each condition, 5 × 10^4^ viable cells were taken to prepare ATAC-seq reactions following the Omni-ATAC protocol [33]. Double size selection and cleanup of the amplified libraries was carried out with AMPure XP beads (A63880, Beckman Coulter), using 1:0.5 and 1:1.8 sample-to-reagent ratios. Purified libraries were subjected to 2 × 150 bp paired-end sequencing on an HiSeqX 10 sequencing device (Illumina, San Diego, CA, USA) at 100 million reads per sample at Macrogen (Macrogen Inc., Seoul, Republic of Korea).

### 2.13. ATAC-seq Data Processing and Analysis

Removal of adapter sequences and quality filtering of the raw sequencing data was performed with Trimmomatic [34]. Paired reads were mapped to the human reference genome (Ensembl GRCh38) using Bowtie2 [35], with a maximum insert size of 1000 bp. PCR duplicates were removed using Picard MarkDuplicates [36] and SAMtools [37] was used to remove reads that mapped to the mitochondrial genome and retain properly paired map reads with a MAPQ score higher than 30. Bam files were converted to bigwig files using deepTools [38] for visualization in the IGV genome browser [39]. ATAC-seq peaks were called collectively from two independent biological replicates using Genrich [40], using the ATAC-seq mode and considering an AUC value higher than 200 and an adjusted (adj.) *p*-value for the peak calling below 0.01 as thresholds. The ENCODE blacklisted regions for the GRCh38 (ENCFF356LFX) were filtered out from the called peaks. The R/Bioconductor package ChIPseeker was used to annotate the genomic location of peaks [41]. Peaks found within ±3 kb around the transcriptional start site (TSS) of annotated genes were considered as proximal (which included the ChIPseeker categories “5’ UTR” and “First exon”). The rest of the peaks were considered as distal. Differential accessibility analysis of TGFβ1-treated samples versus solvent-treated was carried out following a previously described pipeline [42], that employed the R/Bioconductor package csaw [43] to count reads within peaks and for non-linear loess-based normalization. Subsequently, the R/Bioconductor package edgeR [44] was used to identify differentially accessible regions (DARs). Chromatin accessibility changes were considered significant with an adj. *p*-value cutoff of 0.05. De novo TFBM discovery within the identified peaks was performed with HOMER [22].

### 2.14. RNA Sequencing (RNA-seq) Data Processing and Analysis

For time-resolved transcriptome analyses of TGFβ1 or solvent-treated MCF10A cells, total RNA was submitted to the Genome and Proteome Core Facility of the German Cancer Research Center, (Heidelberg, Germany). Sequencing libraries were prepared using the TruSeq Stranded RNA Sample Prep Kit (Illumina) and paired-end sequenced (2 × 100 bp) on an HiSeq 4000 sequencing device (Illumina) at 50 million reads per sample. For transcriptome analyses of *JUNB* WT and mutant (mut) cell clones, total RNA preparations were submitted to Macrogen Inc. Sequencing libraries were generated using the TruSeq RNA Sample Prep Kit v2 (Illumina) and subjected to 2 × 150 bp paired-end sequencing on a NovaSeq 6000 sequencing device (Illumina) at 50 million reads per library. Removal of adapter sequences and quality filtering of the raw sequencing data was performed with Trimmomatic [34]. Paired reads were mapped to the human reference genome (Ensembl GRCh38) and the read counts per gene were obtained using the STAR aligner [45]. Read counts were normalized according to library size and used for the identification of differentially expressed genes (DEGs) using the R/Bioconductor package Limma [46]. Specifically, the design matrix which was used in the voom function of Limma, was based on the model: matrix (~0 + condition), in which “condition” was defined by the different time points of TGFβ1 treatment and in combination with cellular genotypes where applicable. As a result, all possible pairwise comparisons were considered. Thereby, DEGs were identified between TGFβ1-treated samples from MCF10A cells versus the corresponding solvent-treated samples of each experiment. Similarly, DEGs were identified comparing *JUNB* mut cell clones versus *JUNB* WT cell clones, in solvent and in each TGFβ1-treated sample. Time series and clustering analysis of genes which were DEGs in at least one condition when comparing TGFβ1-treated versus solvent-treated MCF10A cells was carried out by applying fuzzy c-means clustering and the R package e1071 [47,48]. The z-scores of normalized values of log_2_ fold changes (FCs) were used for clustering analysis.

### 2.15. Combined Analysis of Time-Resolved RNA-seq and ATAC-seq Data

First, by considering all DEGs and DARs from the entire time series, we identified DEGs that had at least one DAR annotated to them. Next, correlation analysis was performed between the z-scores of normalized values of log_2_FCs associated to DEGs and DARs. As selecting criteria, a pair of proximal or distal DARs and a DEG was considered highly correlated with an absolute value of the Pearson correlation coefficient higher than 0.7, or with an absolute value of the Spearman rank correlation coefficient equal to 1. Clustering analysis of DAR-DEG pairs that fulfilled the correlation criteria, was carried out by applying fuzzy c-means clustering and the R package e1071 [47,48]. For this, again, the z-scores of normalized values of log_2_FCs were used. Functional enrichment analysis was carried out for DEGs belonging to the identified clusters, using the GO term collection “Biological Processes” [49,50]. Functional enrichment analysis, as well as gene enrichment analysis of known EMT genes were evaluated by Fisher’s exact test. TFBM activity analysis and prediction of TF downstream targets was carried out using IMAGE [23]. At first, IMAGE was applied to the distal and proximal DARs separately. In a second step, TFBM activity changes that occurred at both distal and proximal DARs were identified. The z-scored normalized values of the average motif activities and predicted target gene expression were used for illustrating the results of IMAGE for the TFs positioned at both proximal and distal DARs.

### 2.16. Survival Analysis

We defined the 274 genes forming the intersection of JUNB direct target genes predicted by IMAGE and the DEGs identified by the comparative transcriptome analyses of *JUNB* WT and mut cell clones, as the TGFβ-regulated EMT signature. The GEPIA2 web server [51] was employed to perform correlation analyses between the average expression of this signature and the average expression of the REACTOME gene set “Signaling by TGF beta receptor complex” (Stable identifier: R-HSA-170834) across transcriptomic datasets from The Cancer Genome Atlas (TCGA). For, this the mean of the log2(TPM+1) values of the components of the two signatures was calculated as described [51], whereby genes common to both signatures were excluded from the analyses. The impact of the TGFβ-regulated EMT signature defined in this study on cancer patient survival was analyzed using clinical and gene expression data from TCGA. As an initial step, correlation analyses were carried out to select those components of the TGFβ-regulated EMT signature that actually showed cancer-type-specific correlated expression with at least 33% of the genes from the REACTOME gene set “Signaling by TGF beta receptor complex”. Based on this, individual genes with an absolute value of the Pearson correlation coefficient higher than 0.25 were chosen for survival analysis using multivariate Cox proportional hazards regression for each cancer entity investigated. The R packages survival and ggplot were used to fit the Cox proportional hazards models and to generate Kaplan-Meyer plots [52,53], respectively. Patients were stratified into high and low risk groups with a log-rank *p*-value < 0.05. The number of samples with available survival, staging, and subtype information used for these analyses were 473 (breast invasive carcinoma [BRCA]), 140 (lung squamous cell carcinoma [LUSC]), 185 (colon adenocarcinoma [COAD]), and 151 (pancreatic adenocarcinoma [PAAD]).

## 3. Results

### 3.1. TGFβ Pathway Activation Increases Chromatin Accessibility Predominantly at Promoter-Distal Candidate DNA Regulatory Elements

**Figure 1:**
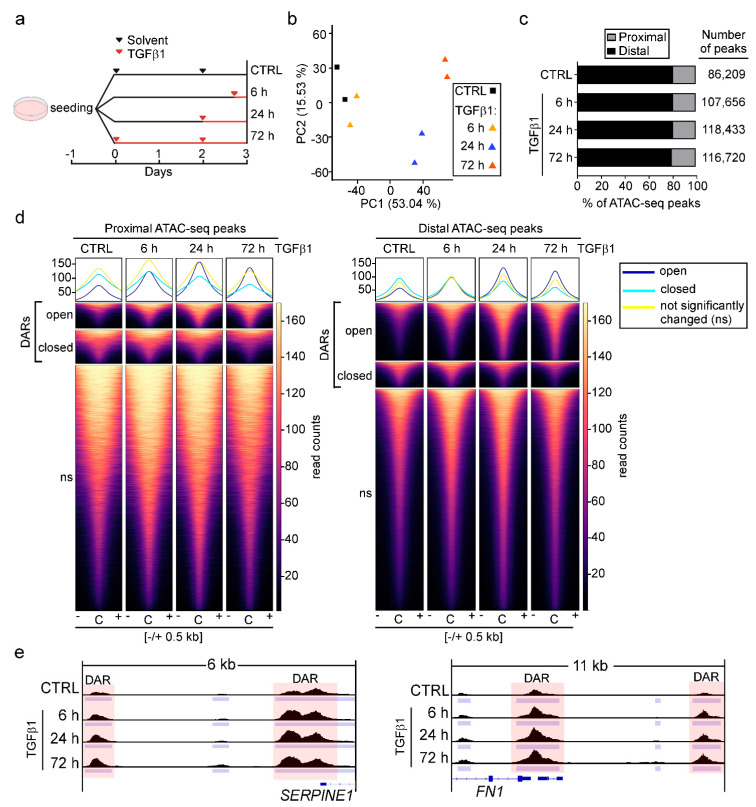
TGFβ pathway activation predominantly increases chromatin accessibility at promoter-distal regions. (**a**) Experimental setup for the ATAC-seq time course experiment. MCF10A cells were seeded in four cell culture dishes and treated with 5 ng/mL TGFβ1 as indicated. A control (CTRL) plate received solvent solution for 72 h. All samples were harvested and subjected to ATAC-seq three days after seeding. (**b**) Principal component analysis (PCA) of ATAC-seq data from MCF10A cells treated with solvent or TGFβ1 as described in panel (**a**). For each condition, two independent biological replicates were analyzed. (**c**) Proportions of ATAC-seq peaks across experimental conditions annotated to proximal and distal genomic regions relative to the TSSs of annotated genes. (**d**) Read density heatmaps showing the distribution of ATAC-seq reads aligned to the centers (C) of proximal (**left**) and distal (**right**) peaks. Peaks that were significantly changing over the time course of TGFβ1 treatment compared to CTRL samples were classified as open or closed and denoted as differentially accessible regions (DARs). (**e**) Genome browser views depicting ATAC-seq tracks from CTRL and TGF-β1-treated samples for *SERPINE1* and *FN1*. Peak locations are indicated by light blue bars. Peaks exhibiting statistically significant opening of chromatin are shaded in pink.

To identify and characterize candidate DNA regulatory elements whose activity might change in response to TGFβ1, we treated the human mammary epithelial cell line MCF10A with solvent or TGFβ1 for 6, 24, and 72 h and subsequently performed ATAC-seq (Figure 1a). Principal component analysis (PCA) showed that biological replicates were highly similar. Furthermore, we observed clear segregation of TGFβ1-treated samples from each other and from control samples along the principal component 1 (53.04% of variance explained), likely reflecting differences induced by exposure to TGFβ1 (Figure 1b). We annotated all detectable ATAC-seq peaks and classified them as proximal if they were located within the interval of −3 kb to +3 kb of transcriptional start sites (TSS) of annotated genes. All ATAC-seq peaks outside of this range were classified as distal to TSSs. Remarkably, while TGFβ1 treatment increased the total number of ATAC-seq peaks by approximately 28,000 compared to the solvent-treated sample, the relative proportions of proximal and distal peaks did not change over the time of TGFβ1 treatment. Approximately 80% of all peaks were found to localize distally (Figure 1c).

Furthermore, we identified differentially accessible regions (DARs) which exhibited statistically significant opening or closing of chromatin structure in TGFβ1-treated samples compared to the solvent controls. The overall number of DARs increased from 537 in samples treated with TGFβ1 for 6 h to 25,976 in samples treated with TGFβ1 for 72 h (Figure S1; Table S6). The majority of DARs exhibited chromatin opening (Figure S1). However, when considering proximal and distal DARs separately, a comparatively smaller proportion of TSS-proximal DARs showed this behavior (Figure 1d and Figure S1). Thus, after 72 h of exposure to TGFβ1, only 43% of TSS-proximal DARs exhibited chromatin opening, whereas 72% of TSS-distal DARs gained chromatin accessibility. This difference can be accounted for by the large fraction of instances where multiple distal, open DARs appear to be linked to a single TSS (Figure S1). Given that distal DARs often represent transcriptional enhancer elements, this observation is in agreement with the notion that in many cases multiple enhancers are connected to one gene and may cooperate in its activation [54]. Furthermore, consistent with the observed preponderance of chromatin opening, proximal and distal DARs associated with the known TGFβ1-responsive genes *SERPINE1* and *FN1* displayed a rapid and continuous increase in accessibility over the time course of TGFβ1 treatment (Figure 1e).

### 3.2. Changes in Chromatin States Parallel Transcriptional Reprogramming Indicative of TGFβ1-Induced EMT

**Figure 2:**
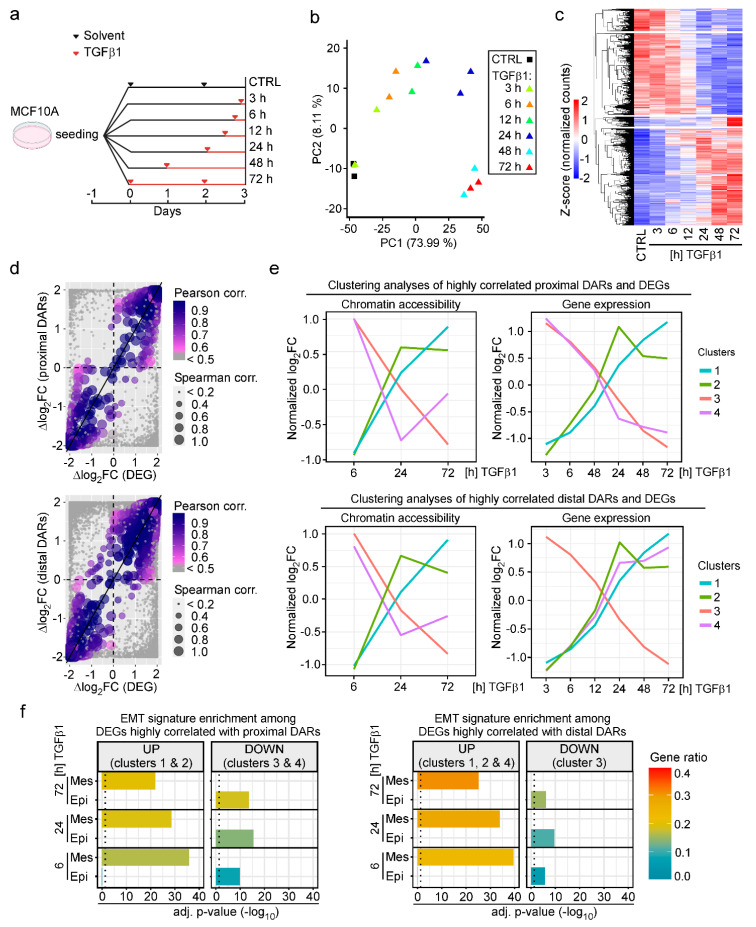
TGFβ1-induced changes in chromatin accessibility and gene expression changes are highly correlated and indicate acquisition of an EMT phenotype. (**a**) Experimental setup of the RNA-seq experiment. MCF10A cells were seeded in seven cell culture dishes and treated with 5 ng/mL TGFβ1 as indicated. A control (CTRL) sample was treated with solvent solution for 72 h. All samples were harvested and processed for RNA isolation three days after seeding. (**b**) PCA of RNA-seq data from MCF10A cells treated with solvent or TGFβ1 as described in panel (**a**). For each condition, two independent biological replicates were analyzed. (**c**) Heatmap showing all genes differentially expressed (adj. *p*-value < 0.05) in MCF10A cells during the time course of TGFβ1 treatment. (**d**) Top: Correlation analysis of ∆log_2_FCs of proximal DARs and differentially expressed genes (DEGs). Bottom: Correlation analysis of ∆log_2_FCs of distal DARs and DEGs. To take into account the time-course nature of the ATAC-seq and RNA-seq experiments, the log_2_FCs of chromatin accessibility and gene expression dynamics were normalized across the experimental time points, and the slopes of the log_2_FCs during TGFβ1 treatment were determined as ∆log_2_FC = log_2_FC(72 h) − log_2_FC(6 h). In cases where multiple distal DARs were linked to a particular DEG, all DAR-DEG pairs were depicted as separate dots. Dot color and dot size indicate Pearson and Spearman correlation coefficients, respectively. Grey dots represent non-correlated DARs and DEGs. (**e**) Clustering analyses of time series data of highly correlated proximal DARs and DEGs (**top**) and highly correlated distal DARs and DEGs (**bottom**). The centroids of the four different clusters identified by the fuzzy c-means method of highly correlated DEGs and DARs are depicted in the plots. (**f**) Left: Enrichment analysis of published EMT signatures among DEGs highly correlated with proximal DARs for each time point of TGFβ1 treatment compared to CTRL samples. Upregulated (UP) genes correspond to genes from clusters 1 and 2 in panel (**e**). Downregulated (DOWN) genes correspond to clusters 3 and 4 in panel (**e**). Right: Enrichment analysis of published EMT signatures among DEGs highly correlated with distal DARs for each time point of TGFβ1 treatment compared to CTRL samples. Upregulated (UP) genes correspond to genes from clusters 1, 2, and 4 in panel (**e**). Downregulated (DOWN) genes correspond to cluster 3 in panel (**e**). These analyses used the sum of five different published EMT signatures as a core signature. Genes within the EMT signature were classified as mesenchymal (Mes) or epithelial (Epi) according to the annotations provided in the original publications. The color of the bars indicates the enrichment ratio. The length of the bars shows statistical significance. The dotted line indicates an adj. *p*-value < 0.05.

To be able to integrate dynamic changes in chromatin accessibility and gene expression induced by TGFβ1, we next performed transcriptome analyses. For this, we treated MCF10A cells for increasing times with TGFβ1 and conducted RNA-seq (Figure 2a). PCA indicated good concordance of independent biological replicates, while TGFβ1-treated samples showed a clear segregation along the principal component 1 (73.99% of variance explained), likely a result of TGFβ1-mediated gene expression changes over time (Figure 2b). The number of differentially expressed genes (DEGs) steadily increased over time with the highest number of DEGs observed after 72 h of TGFβ1 treatment (Figure S2; Table S7). Although at early time points of TGFβ1 stimulation slightly more genes were upregulated, near equal numbers of DEGs were up- and downregulated after 48 h and 72 h of TGFβ1 exposure (Figure 2c and Figure S2).

Next, we examined the relationships between changes in chromatin accessibility and gene expression. Correlation analysis indicated that opening and closing of chromatin structure at proximal and distal DARs was paralleled by up- and downregulation of the associated DEGs whereby nearest neighbor relationships were applied to link distal DARs and genes (Figure 2d). For more refined analyses, sets of highly correlated DARs and DEGs were further characterized by fuzzy c-means clustering. Thereby, four distinct clusters which differed by their temporal profiles of TGFβ1-induced alterations in chromatin accessibility and gene expression, were identified each for the highly correlated proximal and distal pairs of DARs and DEGs (Figure S3). A membership score was calculated for all cluster-constituent genes, representing how close the genes were to the cluster centroids (Figure S3). By focusing on the cluster centroids, clusters 1 and 2 of highly correlated proximal DARs and DEGs represent cases of TGFβ1-induced increases in chromatin accessibility and gene expression, whereas clusters 3 and 4 represent cases where decreased chromatin accessibility was paralleled by downregulation of gene expression in response to TGFβ1 (Figure 2e, top). Similarly, clusters 1, 2, and 3 are formed by cohorts of highly correlated distal DARs and DEGs where chromatin accessibility and gene expression changed in same direction (Figure 2e, bottom). In contrast, cluster 4 comprises of a set of distal DARs which exhibit a gradual loss of accessibility over the time course of TGFβ1 treatment while expression of their associated DEGs continuously increased.

To obtain information about the biological program(s) linked to DEGs from highly correlated sets of DARs and DEGs, we subjected them to functional enrichment analysis. Gene ontology (GO) terms related to developmental processes, extracellular matrix organization, cellular movement, cellular junctions and adhesion, actin filament organization, and response to TGFβ signaling were among the most significantly positively enriched categories among cluster 1 and 2 DEGs with proximal DARs, and cluster 1, 2, and 4 DEGs with distal DARs (Figure S4; Tables S8 and S9). Furthermore, DEGs that were downregulated over time (clusters 3, 4 proximal DARs; cluster 3 distal DARs) showed enrichment for GO terms related to various metabolic processes (Figure S4; Tables S8 and S9). Beyond this, it appeared that gene expression changes indicative of alterations of the same biological processes could be accompanied (i) by chromatin opening and closing of promoter-proximal as well as promoter-distal DARs (Figure S4, e.g., cluster 1 and 3 DEGs correlated with proximal or distal DARs); (ii) by distinct temporal profiles of gene expression and chromatin dynamics (Figure S4, cluster 1 and 2 DEGs); and (iii) by parallel or antiparallel dynamics in gene expression and chromatin structure (Figure S4, cluster 1 and 4 of DEGs correlated with distal DARs). These observations hint that genes dedicated to the execution of specific biological processes may be subject to different modes of regulation. Nonetheless, the functional enrichment analysis of DEGs provided unbiased evidence for ongoing EMT which is known to be induced by TGFβ1 in MCF10A cells. Indeed, DEGs up- and downregulated in response to TGFβ1 were exclusively and increasingly enriched among the mesenchymal and epithelial components, respectively, of publicly available EMT gene signatures (Figure 2f) [15,55,56,57,58]. In summary, the global analyses of TGFβ1-induced gene expression changes are indicative of EMT. By extrapolation, this argues that the accompanying chromatin structural changes likewise represent characteristic and informative features of this process.

### 3.3. Analysis of Transcription Factor Motif Activity Predicts AP-1 Subunits JUN and JUNB as Key Regulators of TGFβ1-Induced EMT

**Figure 3:**
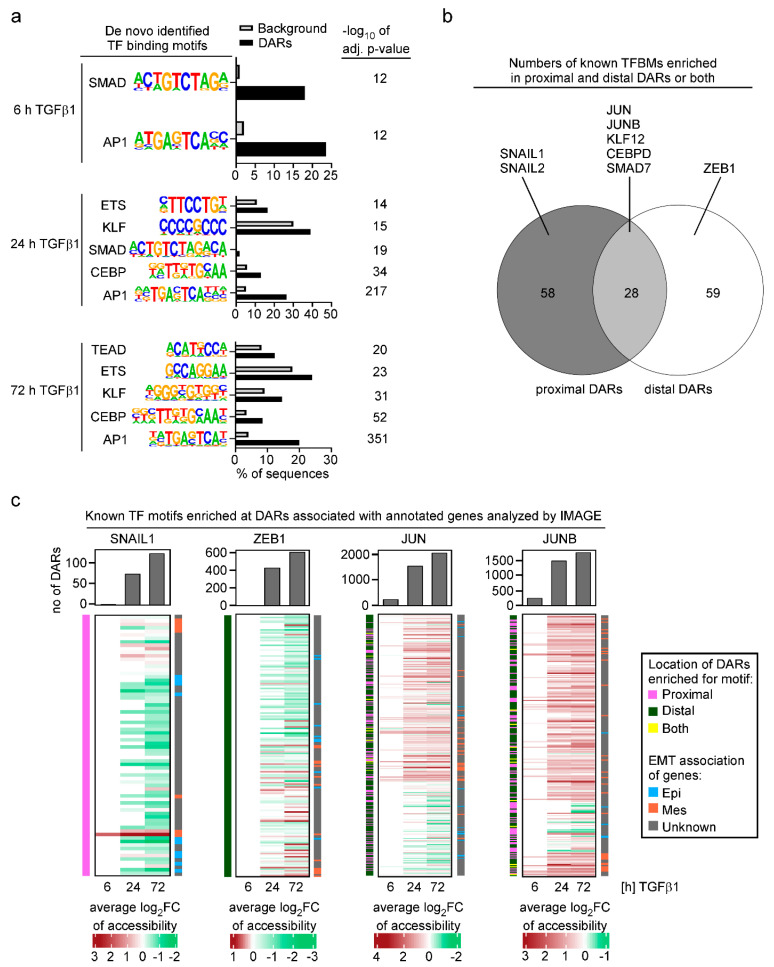
Analyses of TFBM activity suggests a prominent function for AP-1 family members JUN and JUNB in TGFβ1-induced EMT. (**a**) De novo motif analysis of DARs from the TGFβ1 time course experiment using HOMER. For each time point of the TGFβ1 treatment, all significantly identified TF motifs are shown. Bar plots display the percentage of DARs and random background genomics regions in which the motifs were enriched. Statistical significance is shown next to the corresponding bar as −log_10_ of the adj. *p*-value. (**b**) Number of TFBMs enriched in proximal and distal DARs or both according to IMAGE. Examples for TFBMs specifically enriched in proximal, distal, or proximal and distal DARs are given. (**c**) DARs with predicted motif activities of JUN and JUNB are more numerous and have broader regulatory scopes compared to DARs with SNAIL1 and ZEB1 motif activities. For each time point of TGFβ1 treatment, the DARs with predicted activities of SNAIL1, ZEB1, JUN, and JUNB motifs were identified by IMAGE, and their numbers were plotted. The heatmaps below represent, for each time point, the average values of log_2_FC of accessibility calculated for all DARs harboring a given TF motif and which are associated with annotated genes. The annotation bars to the left of each of the heatmaps specify the location of the DARs (proximal, distal, or both) in which the TF motifs are enriched. The annotation bars to the right of each of the heatmaps show whether the genes annotated to the DARs were previously identified as epithelial markers (Epi), mesenchymal markers (Mes), or have no known connection to EMT (unknown).

The results of the ATAC-seq and RNA-seq experiments provided a powerful basis to explore without a priori assumptions which transcription factors could be involved in the TGFβ1-mediated changes in chromatin structure and gene expression. To this end, we subjected identified DARs to de novo TFBM discovery and to TFBM activity predictions using HOMER [22] and IMAGE [23], respectively, aiming to ensure consensus and more profound insights. In agreement with previous analyses of TFs involved in EMT [59,60,61], TFBMs which were identified by HOMER and which were significantly more frequently enriched in DARs compared to random genomic sequences, corresponded to the binding motifs for SMAD TFs, and members of the AP-1, ETS, KLF and CEBP TF families (Figure 3a). Among the TFBMs, the AP-1 motif stood out because it was the most significantly enriched TFBM across all time points of TGFβ1 stimulation and was identified in 20–25% of DARs including already the 6 h time point (Figure 3a). Notably, the E-box motif which serves as binding site for classical EMT-TFs such as SNAIL1, SNAIL2, and ZEB1, was not discovered by HOMER in TGFβ1-responsive DARs.

To extend and to complement the TFBM analyses, we further applied IMAGE [23]. IMAGE utilizes a comprehensive collection of known motifs to compute the enrichment of TFBMs at cis-regulatory elements. It further calculates how the identified TFBMs contribute to changes in transcriptional activity by integrating motif activity analyses with gene expression data from RNA-seq experiments. Ultimately, IMAGE identifies with high predictive power positive and negative regulators of a biological process. While IMAGE confirmed the results of HOMER by pinpointing the same TFBMs as being enriched in DARs, it also returned a number of additional TFBMs that had not been discovered by HOMER (Table S10). When considering the distribution of TFBMs identified by IMAGE, it turned out that some of them were selectively found in proximal or distal DARs, while for example binding motifs for JUN and JUNB were present in both types of DARs (Figure 3b; Table S10). JUN and JUNB belong to the large group of TFs which variably homo- and heterodimerize to recognize the AP-1 motif that had already become apparent in the HOMER analysis. Although it is impossible to deduce from TFBM analysis precisely which members of a TF family function through a motif in a given context, when additionally taking into account gene expression data (Figure S5a), JUN and JUNB are the most likely TFs operating at the AP-1 motif in TGFβ1-stimulated MCF10A cells.

Interestingly, in contrast with HOMER, IMAGE identified binding motifs for classical EMT-TFs, but allocated binding motifs for SNAIL1 and SNAIL2 exclusively to proximal DARs while the ZEB1 binding motif was assigned to distal DARs only (Figure 3b,c; Table S10). When comparing the number of DARs harboring the TFBMs, DARs with binding motifs for the SNAIL proteins and ZEB1 were vastly outnumbered by DARs with JUN and JUNB binding motifs (Figure 3c), which could explain why HOMER did not call the SNAIL and ZEB1 binding motifs as significantly enriched. Further examination revealed that the majority of DARs featuring the SNAIL and ZEB1 binding motifs exhibited decreasing chromatin accessibility over the time course of TGFβ1 treatment (Figure 3c). Some of these closing DARs were associated with known marker genes for epithelial cell states which is in line with the reported functions of SNAIL factors and ZEB1 as transcriptional repressors of epithelial gene expression [13,62]. JUN and JUNB binding motifs, on the other hand, were mainly associated with DARs where TGFβ1 treatment resulted in chromatin opening and which often were linked to mesenchymal marker genes (Figure 3c) which suggests that JUN and JUNB could be instrumental for the activation of mesenchymal gene expression. This view is supported by the parallel increase in JUN and JUNB TFBM activity and the expression of their target genes as predicted by IMAGE (Figure S5b). Taken together, the results of the integrated analyses of chromatin accessibility and gene expression changes identified a number of TFBMs and potentially associated TFs which might orchestrate transcriptional reprogramming during TGFβ1-induced EMT in the MCF10A mammary epithelial cell line. Surprisingly, according to these analyses, TFs typically considered to be master regulators of EMT seem to play only minor roles in this process while the AP-1 family members JUN and JUNB might play more central roles.

### 3.4. TGFβ1-Induced EMT Proceeds in the Absence of SNAIL1, SNAIL2, and ZEB1

**Figure 4:**
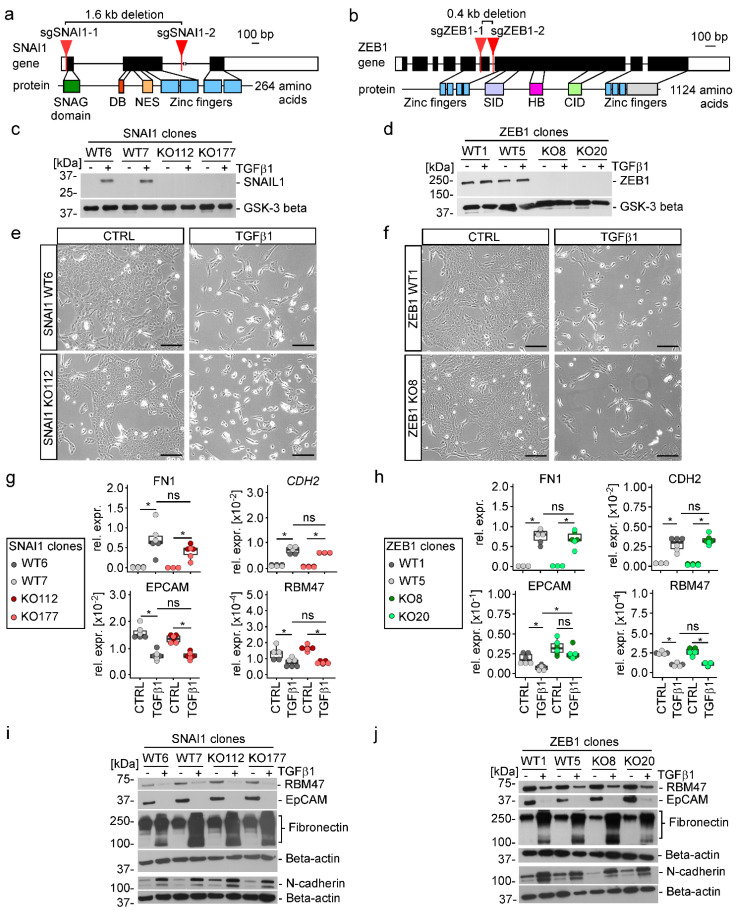
SNAIL1 and ZEB1 are not required for TGFβ1-induced EMT. (**a**,**b**) Schematic representation of the human *SNAI1* and *ZEB1* genes and proteins. Exons are depicted as boxes with protein-coding parts colored in black and UTRs in white. Relevant functional domains of the proteins are indicated by color and linked to the exonic region by which they are encoded. DB: destruction box; NES: nuclear export signal; SID: Smad interaction domain; HB: homeobox; CID: CtBP interaction domain. The locations of sgRNA targets used for inactivation of *SNAI1* and *ZEB1* are marked by red arrows. (**c**,**d**) Detection of SNAIL1 and ZEB1 proteins by Western blot using nuclear extracts from MCF10A *SNAI1* and *ZEB1* wild type (WT) and knock-out (KO) cell clones. Glycogen synthase kinase-3 beta (GSK-3 beta) was used as loading control. Molecular weights are given in kilodaltons (kDa). One representative result from three independent biological replicates is presented. (**e**,**f**) Representative phase-contrast microscopy pictures from one of three independent biological replicates showing the indicated MCF10A *SNAI1* and *ZEB1* WT and KO clones after 72 h of TGFβ1 treatment. The scale bar represents 200 µm. (**g**,**h**) Gene expression analysis of epithelial and mesenchymal marker genes after 72 h of TGFβ1 treatment. RNA levels were measured by qRT-PCR and are shown as relative expression compared to the RNA levels of *GAPDH*. In the box plots each dot represents the result of a single qRT-PCR measurement. Dot color identifies MCF10A *SNAI1* and *ZEB1* WT and KO cell clones. Stars indicate *p*-values corrected for multiple testing by the false discovery rate (FDR) method. *: FDR < 0.05, ns: not significant; Mann-Whitney *U* test. (**i**,**j**) Detection of epithelial and mesenchymal markers after 72 h of TGFβ1 treatment by Western blot using the cytoplasmic fractions of protein lysates from MCF10A *SNAI1* and *ZEB1* WT and KO cell clones. Beta-actin was used as loading control. Molecular weights are given in kilodaltons (kDa). Representative results from one of three independent biological replicates are shown. Uncropped versions of immunoblots including densitometry readings can be found in Figure S23.

To experimentally gauge the impact of SNAIL1, SNAIL2, and ZEB1 on TGFβ1-driven EMT we decided to inactivate their genes. To this end, we made use of CRISPR/Cas9 technology in order to delete parts of the *SNAI1*, *SNAI2*, and *ZEB1* genes encoding important protein domains and to introduce premature stop codons (Figure 4a,b and Figure S6a). Thereby, we generated MCF10A cell clones without detectable expression of SNAIL1, SNAIL2, and ZEB1 (Figure 4c,d and Figure S6b). Additionally, *ZEB1* was inactivated in one of the MCF10A *SNAI1* single knockout (KO) cell clones yielding *SNAI1*-*ZEB1* double KO (DKO) cell clones (Figure S7). As wild-type (WT) controls for each series of KO and the DKO cell clones, we isolated cell clones which had been exposed to the CRISPR/Cas9 system and which had undergone the same cloning procedure but showed no genetic changes at the *SNAI1*, *SNAI2*, and *ZEB1* loci (Figure 4c,d, Figures S6 and S7). When treated with TGFβ1, *SNAI1* and *ZEB1* KO cell clones exhibited morphological changes indistinguishable from their WT counterparts, showing elongated cell shapes and single cell delamination (Figure 4e,f and Figure S8). Likewise, TGFβ1 provoked the up- and downregulation of mesenchymal markers Fibronectin and N-cadherin and of epithelial markers RBM47 and EpCAM, respectively, without discernible differences between MCF10A *SNAI1* and *ZEB1* WT and KO cells (Figure 4g–j). Consistent with these observations, basal and TGFβ1-enhanced migratory capacity of MCF10A cells was not affected by inactivation of *SNAI1* and *ZEB1* (Figure S9a,b). Similarly, the number of single cells that detached from three-dimensional cell aggregates and invaded a surrounding collagen type I matrix did not differ between TGFβ1-treated MCF10A SNAI1 and ZEB1 WT and KO cell clones (Figure S9c,d). A possible explanation why MCF10A cells with single deficiencies in SNAI1 and ZEB1 still underwent TGFβ1-induced EMT, could have been functional redundancy among EMT-TFs. However, TGFβ1-treated MCF10A SNAI1-ZEB1 DKO cell clones still displayed changes in cell morphology and marker gene expression characteristic for EMT (Figure S7). Furthermore, basal and TGFβ1-induced motility of MCF10A cells was maintained, if not increased, in the combined absence of SNAIL1 and ZEB1 (Figure S10a). Likewise, the number of invading single cells was not significantly reduced in SNAI1-ZEB1 DKO cell clones although we observed comparatively larger remnants of the multilobular structures formed by control cells (Figure S10b). Nonetheless, when taking together all of the observations made with the single and double KO cell clones, we conclude that SNAIL1 and ZEB1 are not required for TGFβ1-induced EMT of MCF10A cells which is in agreement with the integrated results of the ATAC-seq and RNA-seq analyses.

In contrast with the inactivation of *SNAI1* and *ZEB1* which had no noticeable impact on the phenotype of MCF10A cells, neither when untreated nor in the presence of TGFβ1, the KO of *SNAI2* strongly affected growth and morphology of MCF10A cells already in the absence of TGFβ1. Compared to WT cells, MCF10A *SNAI2* KO cell clones no longer formed tightly clustered cell islands but grew as small grape-like cell aggregates, possibly due to a defect in cell-substrate adhesion (Figure S6c). In addition, MCF10A *SNAI2* KO cell clones displayed impaired population dynamics which might arise from delayed cell cycle progression (Figure S6d,e). Yet, when treated with TGFβ1, MCF10A *SNAI2* KO cells showed gene expression changes indicative of EMT (Figure S6f,g). However, this has to be interpreted with caution due to the altered phenotype of untreated MCF10A *SNAI2* KO cells and some clonal variability with respect to the expression of *EPCAM* (Figure S6f,g). Nonetheless, we conclude that similar to SNAIL1 and ZEB1, also SNAIL2 is not a major player in TGFβ1-induced EMT. Rather, SNAIL2 seems to exert important functions in naive MCF10A cells which is in line with its reported role as regulator of mammary epithelial cell identity and stemness [63].

### 3.5. TGFβ1-Induced EMT Depends on JUNB in MCF10A Cells

**Figure 5:**
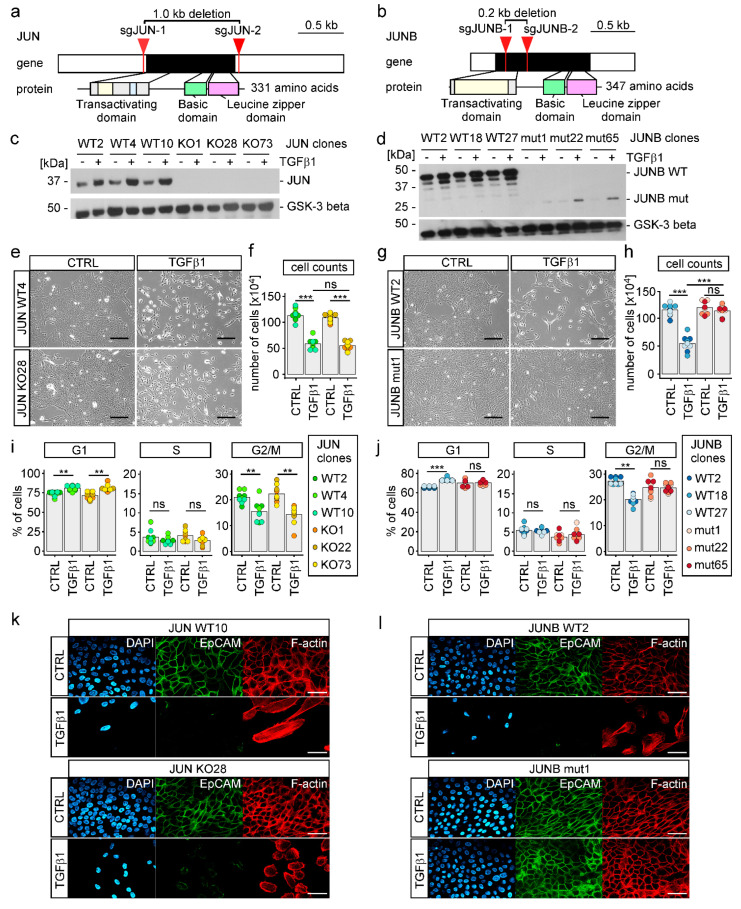
Inactivation of *JUNB* impairs TGFβ1-induced morphological changes and cell cycle progression of MCF10A cells. (**a**,**b**) Schematic representation of the human *JUN* and *JUNB* genes and proteins. Exons are depicted as boxes with protein-coding parts colored in black and UTRs in white. Relevant functional domains of the proteins are indicated by color and linked to the exonic region by which they are encoded. The locations of sgRNA targets used for inactivation of *JUN* and *JUNB* are marked by red arrows. (**c**,**d**) Detection of JUN and JUNB by Western blot using nuclear extracts from MCF10A wild type (WT) and knock-out (KO) or mutant (mut) cell clones. One representative result from three independent biological replicates is shown. Uncropped versions of immunoblots including densitometry readings can be found in Figure S23. (**e**,**g**) Representative phase-contrast microscopy pictures from one of three independent biological replicates showing the indicated MCF10A *JUN* and *JUNB* WT and KO/mut cell clones after 72 h of TGFβ1 treatment. The scale bars depict 200 µm. (**f**,**h**) Cell counts for MCF10A *JUN* and *JUNB* WT and KO/mut cell clones after 72 h of TGFβ1 treatment compared to untreated control (CTRL) are provided. For all cell clones, equal numbers of cells were seeded at the beginning of the experiment. (**i**,**j**) Proportions of MCF10A *JUN* and *JUNB* WT and KO/mut cell clones in G1, S, and G2/M phases of the cell cycle analyzed by flow cytometry. (**k**,**l**) EpCAM expression and intracellular localization as well as F-actin intracellular distribution were analyzed by immunofluorescence staining and decoration with Alexa Fluor 555 phalloidin, respectively, for the MCF10A *JUN* and *JUNB* WT and KO/mut cell clones indicated. Representative images from one out of three independent biological replicates are shown. DAPI was used to stain nuclei of cells. The scale bar depicts 50 µm. In panels (**f**,**h**–**j**), the height of the bars represents mean values, while each dot represents the result of a single independent biological replicate. The color code identifies the cell clones. Stars indicate *p*-values corrected for multiple testing by the FDR method. **: FDR < 0.01, ***: FDR < 0.001, ns: not significant; Mann-Whitney *U* test that had delaminated from the main spheroid structures and invaded the surrounding collagen I matrix (Figure 6g). In summary, elevating the expression and activity of JUNB did not bring about the fully mesenchymal state that was observed upon TGFβ pathway activation. Nonetheless, mainly based on the observed morphological changes and markedly enhanced single cell invasion, JUNB-ER activity imposed an advanced hybrid epithelial/mesenchymal phenotype upon MCF10A cells, indicating that it was sufficient to initiate EMT.

The results of IMAGE had suggested that AP-1 TFs represented by JUN and JUNB could be more critical downstream effectors during TGFβ1-induced EMT compared to classical EMT-TFs. Accordingly, we again employed the CRISPR/Cas9 system and generated single cell-derived MCF10A cell lines with extended deletions in the *JUN* and *JUNB* genes (Figure 5a,b). This yielded MCF10A cell clones with no detectable expression of JUN (Figure 5c). Similarly, MCF10A cell clones in which *JUNB* had been targeted showed no expression of JUNB WT protein. However, in *JUNB* mutant (mut) cell clones, we observed low level, TGFβ1-inducible expression of a smaller polypeptide specifically reacting with the anti-JUNB antibody (Figure 5d). Most likely, this polypeptide is generated by re-initiation of translation at an internal methionine codon and represents a JUNB derivative lacking the transactivation domain. Due to this functional impairment and the much-reduced expression levels, we decided to continue with the *JUNB* mut cell clones for phenotypic analyses. When treated with TGFβ1 for 72 h, MCF10A *JUN* KO cells somewhat unexpectedly still acquired several features indicative of largely unperturbed EMT. This included morphological changes, diminished cell numbers and reduced proliferation, downregulation of EpCAM, rearrangement of actin filaments, and up- and downregulation of mesenchymal and epithelial marker genes (Figure 5e,f,i,k, Figures S11a, S12a and S13a,b). However, MCF10A *JUN* KO cell clones had reduced migratory and invasive capacities under TGFβ1 treatment (Figure S14a,c). This suggests that JUN to some extent contributes to a subset of EMT-associated phenotypic alterations.

In contrast to the rather mild effects resulting from *JUN* deficiency, we found that *JUNB* inactivation severely blunted TGFβ1-induced EMT. When exposed to TGFβ1, MCF10A *JUNB* mut cell clones did not lose their epithelial appearance, continued to expand and proliferate, maintained EpCAM expression and localization at cell-cell interfaces, and did not rearrange their actin cytoskeleton (Figure 5g,h,j,l, Figures S11b and S12b). Furthermore, migratory capacities of MCF10A *JUNB* mut cell clones were strongly impaired, both in the presence and absence of TGFβ1 (Figure S14b). When grown as three-dimensional spheroids embedded in collagen I, *JUNB* mutants showed no invasive behavior (Figure S14d). In agreement with the nonappearance of mesenchymal traits, induction of *FN1* and *CDH2*, coding for Fibronectin and N-cadherin, respectively, was diminished in MCF10A *JUNB* mut cell clones, albeit *EPCAM* and *RBM47* were still downregulated in response to TGFβ pathway activation (Figure S13c,d). Furthermore, expression of *SNAI1* and *ZEB1* turned out to be reduced in JUNB mut cell clones (Figure S13c,d). While this places JUNB upstream of these EMT-TFs and implicates JUNB in their regulation, it does not explain the strong impact of JUNB deficiency on TGFβ1-induced EMT, since SNAIL1 and ZEB1 were not required for this. On the other hand, we noticed that inactivation of JUNB had no impact on the expression of JUN, whereas *JUN* KO cell clones showed higher levels of TGFβ1-inducible JUNB expression (Figure S13c) arguing for some cross regulation and potential compensation of the loss of JUN by JUNB. In sum, we conclude that TGFβ1-induced EMT in MCF10A cells critically depends on JUNB and to a lesser extent on JUN.

**Figure 6:**
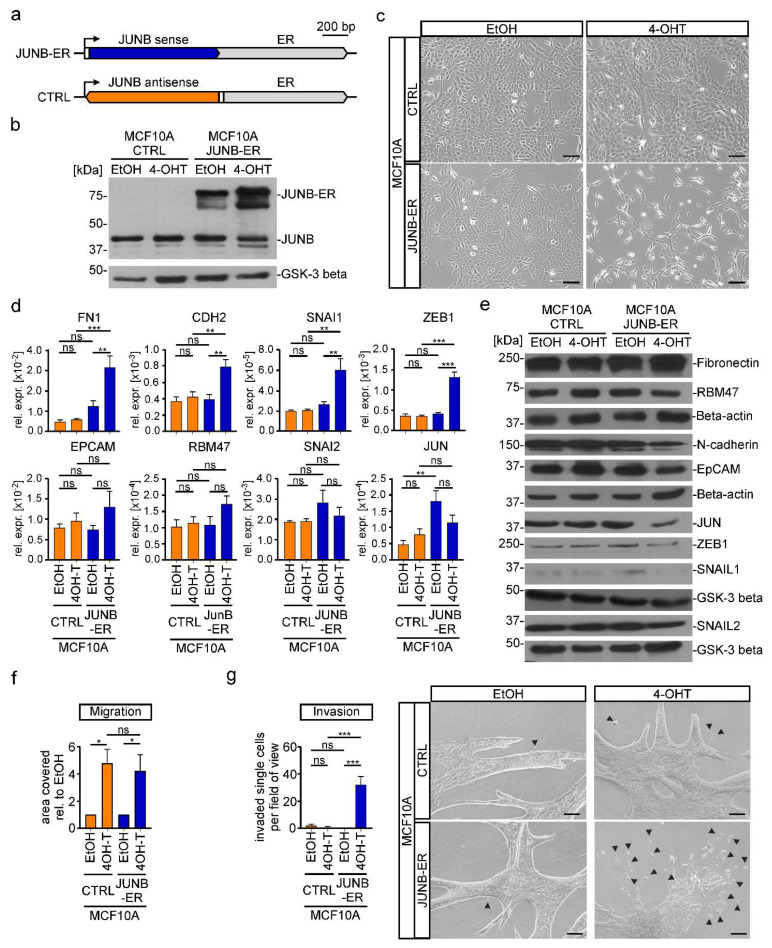
Increased JUNB activity is sufficient to induce EMT in MCF10A cells. (**a**) Schematic representation of the gene expression cassette for a fusion protein consisting of the human *JUNB* coding region (JUNB sense) and a mutant estrogen receptor hormone binding domain (ER). A control construct (CTRL) harbored the JUNB coding region in the opposite orientation (JUNB antisense). The angled arrow indicates the TSS; the white box represents the 5′-untranslated region. (**b**) Simultaneous detection of endogenous JUNB and ectopic JUNB-ER expression by Western blot using nuclear extracts from MCF10A cells that had been stably transduced with a retroviral vector for the expression of JUNB-ER or the control construct. Glycogen synthase kinase-3 beta (GSK-3 beta) was used as the loading control. Molecular weights are given in kilodaltons (kDa). One representative result from three independent biological replicates is presented. (**c**) Representative phase-contrast microscopy pictures from one of three independent biological replicates showing the indicated MCF10A *JUNB-ER* and CTRL cells. The scale bar represents 200 µm. (**d**) Gene expression analysis of epithelial and mesenchymal marker genes in MCF10A *JUNB-ER* and CTRL cells. RNA levels were measured by qRT-PCR and are shown as relative expression compared to those of *GAPDH*. (**e**) Detection of epithelial and mesenchymal markers in the cytoplasmic (Fibronectin, N-cadherin, EpCAM, RBM47) and nuclear fractions (SNAIL1, SNAIL2, ZEB1, JUN) of protein lysates from MCF10A *JUNB-ER* and CTRL cells. Beta-actin and GSK-3beta were used as loading controls. Molecular weights are given in kilodaltons (kDa). Representative results from one of three independent biological replicates are shown. (**f**) Results from transwell migration assays performed with MCF10A *JUNB-ER* or CTRL cells. Depicted is the area covered by cells on the bottom surface of transwell inserts relative to the value of EtOH treated cells. (**g**) Spheroid invasion assays performed with cellular aggregates embedded in a collagen I matrix. For the quantification single cells and small aggregates (exemplarily marked by arrow heads) that had detached from the bulk of the cell aggregates were counted in two fields of view per cell line and condition. Pictures of representative spheroids from one out of three independent biological replicates are shown on the right. The scale bars represent 100 μm. (**b**,**e**) Uncropped versions of immunoblots including densitometry readings can be found in Figure S23. (**b**–**g**) For all experiments, cells were treated with 100 nM 4-OHT or a corresponding volume of ethanol (EtOH) for 72 h prior to harvest. (**d**,**f**,**g**) Bars represent the mean values from at least three independent biological replicates. Error bars depict the standard error of the mean. Stars indicate *p*-values corrected for multiple testing by the FDR method. *: FDR < 0.05, **: FDR < 0.01, ***: FDR < 0.001, ns: not significant; one-way ANOVA.

To test a potential EMT-inducing capacity of JUNB, we stably transduced MCF10A cells with a retroviral construct that enabled expression of a fusion protein consisting of JUNB and a mutant ER hormone binding domain (Figure 6a,b) [27]. Control cells received a construct with the *JUNB* coding region in antisense orientation. Upon activating transcriptional activity of the JUNB-ER fusion by administration of 4-OHT, the cells adopted more elongated, fibroblastoid shapes and displayed a disperse growth pattern (Figure 6c). At the RNA level the mesenchymal markers *FN1*, *CDH2* and the EMT-TFs *SNAI1* and *ZEB1* were upregulated, while for instance expression of the epithelial markers *EPCAM* and *RBM47* remained unchanged (Figure 6d). At the protein levels, though, amounts of N-Cadherin, JUN, ZEB1, and SNAIL1 rather decreased (Figure 6e). Unfortunately, the impact of JUNB-ER on cell motility could not be assessed due to non-specific effects of 4-OHT (Figure 6f). However, in a 3D spheroid invasion assay, MCF10A *JUNB-ER* but not control cells exhibited vastly increased numbers of single cells 

Next, we asked whether JUNB played a critical role as downstream effector of TGFβ1 signaling and had EMT-inducing potential on its own also in other cells. To this end, we utilized the lung adenocarcinoma cell line A549 and, following the same experimental strategies as in MCF10A cells, generated A549 *JUNB* WT and mut cell clones as well as mixed populations of A549 control and JUNB-ER-expressing cells (Figures S15 and S16). However, in A549 cells, *JUNB* deficiency did not prevent TGFβ1-induced alterations in cell shape (Figure S15a,b). Nonetheless, A549 *JUNB* mut cells exhibited changes in the expression of EMT markers and the TF genes *SNAI1*, *SNAI2*, *ZEB1*, and *JUN* (Figure S15b–d). Yet, the genes affected and the direction of their response to *JUNB* inactivation in most cases were different in A549 and MCF10A *JUNB* mut cells (compare Figures S13c,d and S15c,d). Despite this difference, TGFβ1-induced migration and invasion seemed to be impaired also in A549 *JUNB* mut cells (Figure S15e,f). Consistent with expendability of JUNB for TGFβ1-induced changes in the shape of A549 cells, also the JUNB-ER fusion proved to be functionally neutral in this regard (Figure S16). Likewise, JUNB-ER activation had only little or no impact on the expression of selected genes related to EMT (Figure S16c,d). This notwithstanding, JUNB-ER activation enhanced motility of 4-OHT treated A549 cells (Figure S16e), and in the 3D spheroid invasion assay, we observed a larger number of single cells and small cell aggregates that were separated from the bulk of the spheroids (Figure S16e,f). However, JUNB-ER activation did not result in the formation of multicellular protrusions. Yet, when combined, the results from the A549 *JUNB* mut and *JUNB-ER* cells suggest that JUNB does contribute to TGFβ1-induced EMT in these cells, albeit with reduced penetrance compared to MCF10A cells, thereby revealing a context-dependent role of JUNB as inducer and mediator of EMT processes.

### 3.6. JUNB Loss-Of-Function Impairs TGFβ1-Mediated Transcriptional Reprogramming

**Figure 7:**
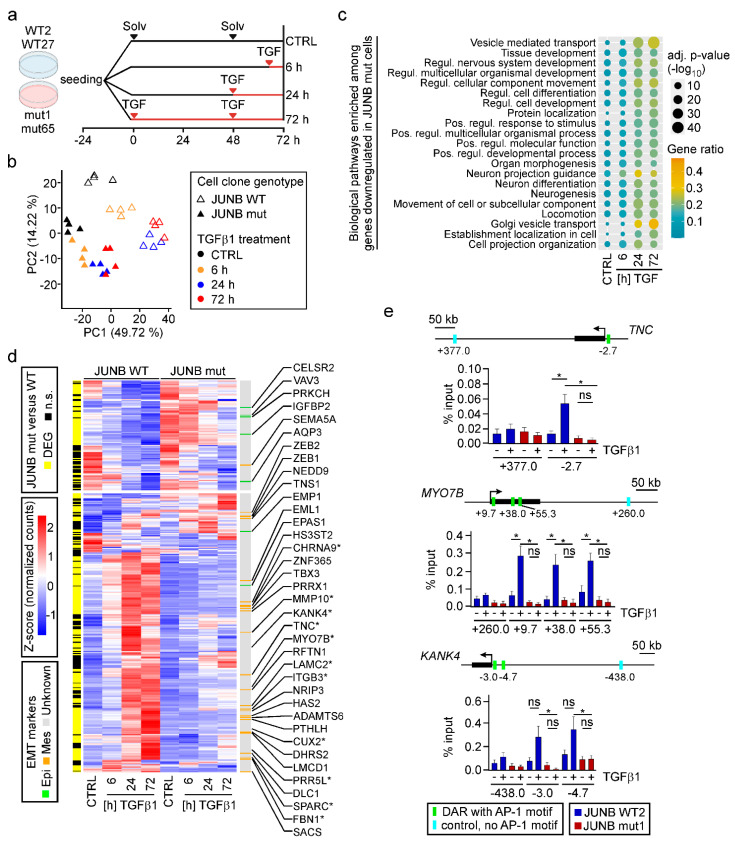
JUNB loss-of-function impairs TGFβ1-induced transcriptional reprogramming. (**a**) Experimental setup for the RNA-seq time course experiment of MCF10A *JUNB* WT and mut clones. Each JUNB clone was seeded in four different plates and treated with 5 ng/mL TGFβ1 (TGF) for the times indicated. Control (CTRL) samples were treated with solvent (Solv) solution for 72 h. All samples were harvested and processed for RNA isolation three days after seeding. (**b**) PCA of RNA-seq data from MCF10A JUNB WT and mut clones treated with solvent or TGFβ1 as described in (**a**). For each condition, two independent biological replicates are analyzed. (**c**) Enrichment analysis of GO terms “Biological Processes” for DEGs showing reduced responses to TGFβ1 in MCF10A *JUNB* mutant cell clones. The dot plot shows the top 21 most significantly enriched GO terms. Dot size indicates statistical significance. Color of the dots indicates gene ratio. (**d**) Heatmap representing the expression of JUNB targets predicted by IMAGE in *JUNB* WT and mut cells. The z-scores of the normalized counts of these genes were plotted in the central heatmap. IMAGE-predicted JUNB targets which turned out to be actual DEGs in *JUNB* WT versus *JUNB* mut cells at least at one of the time-points of TGFβ1 treatment, are indicated in yellow by the sidebar on the left side of the heatmap. Known EMT marker genes are displayed in the color-coded sidebar on the right side of the heatmap. Epi: epithelial; Mes: mesenchymal; unknown: no known connection to EMT. Genes further validated by qRT-PCR are marked with asterisks. (**e**) ChIP-qPCR to validate JUNB binding at DNA regulatory regions associated with candidate target genes. Schemes above the bar plots show for each gene under investigation the locations of transcriptional start sites (angled arrows), the whole gene body including exons and introns (black box) and DARs with AP-1 motifs examined by ChIP-qPCR (green bars). At each locus, a control region (turquoise bars) with no recognizable AP-1 motif was analyzed in parallel. The positions of the PCR amplicons relative to the transcriptional start sites are given in kilobases (kb). The experiment was performed for MCF10A *JUNB* WT2 and mut1 cell clones. Results of the ChIP-qPCR experiments are presented as percentages of the input material (% input). The mean values from five independent biological replicates and the standard error of the mean were plotted. Stars indicate *p*-values corrected for multiple testing by the FDR method. *: FDR < 0.05, ns: not significant; Mann-Whitney *U* test.

Given how strongly *JUNB* loss-of-function had impaired TGFβ1-induced EMT in MCF10A cells, we aimed to comprehensively characterize the JUNB-dependent transcriptome and performed time-resolved RNA-seq with MCF10A *JUNB* WT and mut cell clones (Figure 7a). Initial evaluation of the RNA-seq data by PCA revealed that samples segregated both according to genotype and TGFβ1 treatment (Figure 7b). The clear separation of solvent-treated *JUNB* WT and mut samples additionally hinted that JUNB plays a role not only during TGFβ1-induced EMT but also in naïve MCF10A cells. This is consistent with the fact that MCF10A cells express JUNB also in the absence of TGFβ1 (Figure 5h), and the observed change in cell motility under control conditions (Figure S11b). TGFβ pathway-independent functions of JUNB in MCF10A cells are further suggested by the finding that 1623 genes were differentially expressed in untreated *JUNB* mut versus WT cell clones (Figure S17a, bottom). Beyond this, and as expected, inactivation of *JUNB* severely restricted TGFβ1-mediated transcriptional responses, reducing the number of DEGs to 26%, 32%, and 41% of the numbers observed in *JUNB* WT cells at the different time points of TGFβ pathway stimulation (Figure S17a; Table S11). Of note, the vast majority of genes that responded to TGFβ1 in MCF10A WT cells were not regulated in MCF10A *JUNB* mut cells, and only very few genes showed opposite directions of regulation in the two genetic backgrounds (Figure S17b). To which extent the remaining TGFβ1 response might still be influenced by other AP-1 family members, some of which appear to be significantly upregulated in *JUNB* mut cells (Figure S18), remains unclear. However, the pronounced transcriptional changes caused by *JUNB* loss-of-function identify JUNB as a pivotal regulator of gene expression in MCF10A cells, both in the absence and presence of TGFβ pathway activation.

To characterize the genetic programs under control of JUNB, we performed functional enrichment analyses for DEGs which showed reduced expression in TGFβ1-treated *JUNB* mut cells. GO terms enriched among these DEGs, were found to be related to developmental processes, locomotion, and organization of cell projections (Figure 7c). This matches the phenotypic changes of *JUNB* mut cells when treated with TGFβ1. Interestingly, 65% of the potential direct JUNB target genes predicted by IMAGE were significantly affected by *JUNB* inactivation (Figure 7d). Among this set of 274 genes, we found 24 and 7 known mesenchymal and epithelial markers, respectively (Figure 7d), confirming the tight connection between JUNB, JUNB-dependent gene expression, and TGFβ1-induced EMT in MCF10A cells.

In order to corroborate that IMAGE correctly predicted direct target genes of JUNB, we selected from the 274-gene set the known mesenchymal marker Tenascin C (*TNC*), and two genes which so far had not been linked to JUNB and EMT, namely KN motif and ankyrin repeat domains 4 (*KANK4*), and Myosin VIIB (*MYO7B*) and performed ChIP-qPCR. This demonstrated TGFβ1-inducible binding of JUNB to DARs with predicted JUNB TFBMs in *JUNB* WT but not in *JUNB* mut cells (Figure 7e), thus testifying to the predictive power of IMAGE.

### 3.7. Definition of a TGFβ-Regulated EMT Signature Predictive of Patient Survival across Cancer Types

In MCF10A cells, TGFβ1-induced EMT decisively depended upon JUNB. Therefore, the TGFβ1-regulated and JUNB-dependent transcriptome inevitably comprises of genes crucial for EMT. The 274 genes representing with high likelihood direct targets of JUNB constitute a particularly relevant subset within the TGFβ1- and JUNB-coregulated gene expression profile. We termed this gene set TGFβ-regulated EMT signature and subjected it to further investigation. For a selection of the TGFβ-regulated EMT signature (marked by asterisks in Figure 7d) both with and without previously established connections to EMT, we examined inducibility by TGFβ1 and the contribution of JUNB to its regulation in different experimental settings and cellular backgrounds. In MCF10A cells, upregulation by TGFβ1 and JUNB-dependent expression could be verified for all genes tested (Figure S19). Except for *MYO7B* and *PRR5L*, these genes were also upregulated in 4-OHT-treated MCF10A *JUNB-ER* cells (Figure S20). Interestingly, TGFβ1-inducible expression of *TNC*, *FBN1*, *LAMC2 SPARC*, and *PRR5L* was attenuated not only in MCF10A *JUNB* mut cell clones but also in *JUN* KO clones (Figure S19). This might explain the observed migratory defect of *JUN* KO cells. It is also in line with the differential requirements for JUN and JUNB in TGFβ1-induced EMT of MCF10A cells.

TGFβ1-inducible expression of *JUNB* and the selected members of the TGFβ-regulated EMT signature could additionally be demonstrated in human immortalized mammary epithelial cells (IMECs) and A549 cells, although temporal profiles and the magnitude of gene expression changes differed (Figure S21). In the pancreatic cancer cell line PANC1, *JUNB* and four of the other eight genes analyzed showed a positive TGFβ1 response (Figure S21). Gene expression analyses in A549 *JUNB* WT and mut cell clones and in A549 *JUNB-ER* and control cells, however, failed to confirm that JUNB played a role in the TGFβ1-mediated regulation of these genes in the lung cancer cell line (Figure S22). Thus, a highly similar group of EMT-related genes appears to be regulated by TGFβ1 in different cellular backgrounds albeit through JUNB-dependent and JUNB-independent mechanisms. 

**Figure 8:**
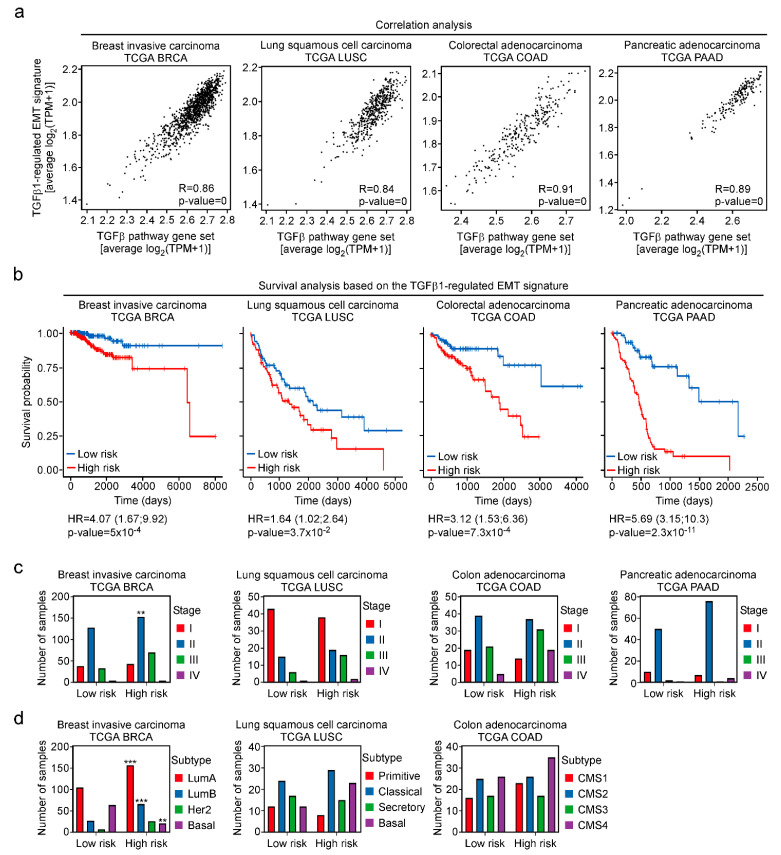
The TGFβ-regulated EMT signature can be predictive for patient survival across cancer types. (**a**) TCGA RNA-seq datasets were used to calculate the correlation between the average expression of the TGFβ-regulated EMT signature and the average expression of the REACTOME gene set “Signaling by TGF beta receptor complex” in breast invasive carcinoma (BRCA), lung squamous cell carcinoma (LUSC), colon adenocarcinoma (COAD), and pancreatic adenocarcinoma (PAAD). The Spearman correlation coefficients (R) and the *p*-values of the correlations are indicated for each dataset. (**b**) Kaplan–Meier curves for overall survival of patients in the TCGA transcriptomic datasets of BRCA, LUSC, COAD, and PAAD. For each dataset, those genes from the TGFβ-regulated EMT signature whose expression correlated with that of the REACTOME gene set “Signaling by TGF beta receptor complex” (absolute value of R greater than 0.25) were selected. The filtered gene sets were used to stratify patients into the low-risk and high-risk of death groups by applying a multivariate Cox proportional hazard regression model. Hazard ratios of each dataset are given together with the *p*-value (log-rank test) to show statistical significance of the estimated models. (**c**,**d**) Numbers of samples representing different tumor stages or cancer subtypes in low and high risk groups. No subtype information for PAAD was available. Stars indicate *p*-values corrected for multiple testing by the FDR method. **: FDR < 0.01, ***: FDR < 0.001; Fisher’s exact test.

In order to extend the analyses beyond cell culture experiments, we turned to TCGA cancer transcriptome data. Correlation analyses revealed that average expression of the TGFβ-regulated EMT signature and the REACTOME gene set “Signaling by TGF beta receptor complex” were positively correlated in breast invasive carcinomas (BRCA), lung squamous cell carcinomas (LUSC), colon adenocarcinomas (COAD) and pancreatic adeno-carcinomas (PAAD) (Figure 8a). This argues that the TGFβ pathway impinges on the expression of the 274-gene set not only in MCF10A and other cell lines but also in a large number of tumors from multiple cancer types.

Finally, we investigated whether the TGFβ-regulated EMT signature might have prognostic value for cancer patient survival. To ascertain a tight connection to TGFβ1 pathway activity and to take into account the direction of regulation, we restricted the analyses to those genes from the signature the expression of which was actually correlated with at least 33% of the components of REACTOME gene set “Signaling by TGF beta receptor complex”. This filtering step was carried out independently for the transcriptomes from the TCGA BRCA, LUSC, COAD, and PAAD cohorts. The filtered gene sets were subsequently used for survival analyses by a multivariate Cox proportional hazards model. Thereby, patients from all four cancer types could be segregated into low risk and high risk groups with significant log-rank *p*-values (Figure 8b), indicating that expression of the TGFβ-regulated EMT signature can be used to predict survival probability of cancer patients. Notably, this does not simply reflect tumor progression because numbers of patients at high risk were not consistently elevated in advanced tumor stages or in more aggressive subtypes across the cancer entities examined (Figure 8c,d).

## 4. Discussion

Growing evidence that TFs typically portrayed as EMT master regulators may be dispensable for EMT [6,7,17,18,19,64], led us to re-assess the molecular mechanisms underlying this process. To this end, we used an unbiased computational approach that integrated time-resolved analyses of chromatin structure and differential gene expression in the widely-used MCF10A cell model of TGFβ1-inducible EMT. The results of this were indicative of comparatively minor contributions of SNAIL proteins and ZEB1 whereas for instance AP-1 subunits JUN and JUNB were predicted to have a much larger impact on TGFβ1-induced EMT. We experimentally confirmed these predictions by showing that indeed SNAIL proteins and ZEB1 were not required for TGFβ1-induced EMT in MCF10A cells, while inactivation of JUNB largely abrogated this process. However, the capacity of JUNB to act as inducer and mediator of EMT appeared to be cell-type-specific. Irrespective of this, by intersecting the JUNB-dependent gene expression profile and IMAGE-based prediction of direct JUNB targets in MCF10A cells, we delineated a 274-gene set which was controlled by TGFβ1 in JUNB-dependent and independent ways in mammary epithelial cells, lung adenocarcinoma and pancreatic cancer cell lines, and showed correlated expression with TGFβ signaling in breast, lung, colon, and pancreatic cancer transcriptomes. Notably, expression of this signature allowed to stratify patients from several cancer types into low and high-risk groups. While this segregation appeared to be independent of tumor stages and subtypes, other clinical parameters might still be correlated with TGFβ pathway activity and could be reflected by the observed survival data. Irrespective of this, the newly identified TGFβ-regulated EMT signature may be a pan-cancer indicator of EMT and prove useful for diagnostic and potentially therapeutic purposes. 

As an entry point to identify putative DNA regulatory elements and associated TFs of potential importance in TGFβ1-induced EMT of the human MCF10A mammary epithelial cell line, we systematically catalogued genomic regions displaying increased accessibility under control conditions and at several time points after stimulation with TGFβ1. Thereby we covered fully epithelial, transitory hybrid, and fully mesenchymal cell states. The majority of open chromatin regions was found to be located distally from TSSs and might represent to a large extent transcriptional enhancers [20,21,65]. Furthermore, the larger part of the ATAC-seq peaks was unaffected by TGFβ1 treatment. This differs from previous results describing widespread and pervasive opening of chromatin in a model of normal murine mammary gland cells [66]. This discrepancy may be explained by species-dependent differences but also by overall shorter exposure to TGFβ1 and prior serum starvation of the murine cells [66]. We also detected 25,976 DARs in TGFβ1-treated MCF10A cells, again mostly at distal positions. Interestingly, while chromatin opening prevailed at distal DARs, roughly equal numbers of proximal DARs eventually exhibited increased and decreased accessibility. The divergent behavior of proximal and distal DARs is a novel aspect not noticed before [67,68]. Of note, chromatin dynamics at TSSs more closely reflect the relative proportions of up- and downregulated genes which is not mirrored by the predominant opening of distal DARs. The imbalance between the number of distal DARs showing chromatin opening and the number of up-regulated genes is likely to be a consequence of multiple enhancers acting upon the same promoter region [20,54]. It cannot be excluded, though, that there are also instances where chromatin structural changes at distal DARs are uncoupled from transcriptional responses of associated genes. This functional separation may involve so-called poised enhancers which are structurally evident yet functionally inert [20,65].

The analysis of TGFβ1-induced chromatin reorganization in MCF10A cells was complemented by time-resolved RNA-seq. Consistent with previously published work, TGFβ1-treated cells showed progressive and extensive transcriptional changes while undergoing EMT [5,8,17,66,67,69]. As reported for other systems [70,71], we observed that gene expression changes correlated with alterations in chromatin accessibility, and that these were mostly, but not always, colinear. An interesting exception to this parallel behavior is provided by the group of highly correlated distal DARs and DEGs where the DARs over time showed a decrease in accessibility while expression of their associated DEGs increased. We hypothesize that these DARs represent transcriptional silencer elements [72]. Transcriptional silencers feature open chromatin in their functional state, yet negatively regulate gene expression [72]. Accordingly, upon physiological or experimental inactivation of silencers their target genes are re-expressed [72,73]. Of note, the candidate silencers from TGFβ1-treated MCF10A cells did not significantly overlap with published collections of silencers from leukemic and hepatic cells [73], possibly due to a high degree of cell type specificity which is a shared property of transcriptional silencers and enhancers [65,72]. 

The central concern of our study was to obtain information about candidate TFs that might operate during TGFβ1-driven EMT and bring about the observed chromatin structural and gene expression changes. For this purpose, we applied two different approaches for TFBM analyses both of which implicated binding motifs for members of the SMAD, ETS, KLF, TEAD, and AP-1 TF families in TGFβ1-induced EMT. This is fully in line with results of comparable analyses in diverse model systems [4,6,59,66,67,74], thus supporting reliability of our approach and arguing that these TF families may be universally involved in EMT processes. In contrast to the TFBMs mentioned above which were readily and repeatedly detected in DARs, SNAIL and ZEB binding motifs were identified only by IMAGE, already hinting that SNAIL1 and ZEB1 might be of limited importance in EMT regulation, at least in MCF10A cells. This idea was further substantiated by the observation that SNAIL and ZEB1 binding motifs appeared to be associated with comparatively fewer DARs that mainly lost accessibility in response to TGFβ1, and were found predominantly in proximal (SNAIL) and distal (ZEB1) positions. The latter, however, is in perfect agreement with results of genome-wide binding studies showing similar genic preferences of SNAIL proteins and ZEB1 [16,74,75,76,77] which again underscores the significance of the TFBM analyses.

In order to experimentally confirm the bioinformatic predictions, we inactivated the *SNAI1*, *SNAI2*, and *ZEB1* genes. *TWIST1*, *TWIST2*, and *ZEB2* were not considered because these genes were not expressed or did not respond to TGFβ1 treatment in our MCF10A cells. Strikingly, individual or combined inactivation of *SNAI1* and *ZEB1* did not noticeably impair TGFβ1-induced EMT. Likewise, *SNAI2* was not obviously required for EMT but sustained proliferation and adhesion of MCF10A cells in the absence of TGFβ1. Although SNAIL1 and SNAIL2 on occasion may function interchangeably [28], and a role for SNAIL2 in EMT of MCF10A cells was proposed [67], the unique requirement for SNAIL2 exhibited by TGFβ1-treatment naïve MCF10A cells, precisely fits to its well-documented roles in mammary gland tissue homeostasis [63].

Our findings that SNAI1 and ZEB1 were dispensable for TGFβ1-induced EMT of MCF10A cells, extend the growing list of studies reporting that EMT processes can proceed in the absence of these TFs [5,6,7,17,18,19,64]. However, our results contrast with the widely held view of SNAIL1 and ZEB1 being EMT master regulators [9]. They also differ from previous reports in which shRNA- or siRNA-mediated knockdown of *SNAI1* and *ZEB1* impaired TGFβ1-induced EMT [62,78]. An explanation for this discrepancy could be the use of the CRISPR/Cas9 system in more recent studies [5,17,18,64] versus the use of shRNAs and siRNAs in earlier work. Overexpression of siRNAs and shRNAs may lead to competition between the exogenous RNA species and endogenous miRNAs and may saturate the RNA interference machinery [79,80]. This could offset EMT regulatory circuits which are known to involve miRNAs [81,82,83]. As a consequence, loss of function experiments involving RNA interference might have overestimated the regulatory potency of SNAIL1 and ZEB1. Still, it is indisputable that ectopically expressed SNAIL1 and ZEB1 can trigger EMT processes, also in MCF10A cells [84,85,86,87]. The EMT-inducing capacity of overexpressed SNAIL1 and ZEB1 probably played a major role in shaping the view of these TFs being EMT master regulators. Yet, while SNAIL1 and ZEB1 appear to be sufficient to induce EMT, apparently, they are not obligatory for the process. Clearly, further investigations are needed to clarify the role of these factors in EMT.

The SNAIL, TWIST, and ZEB TF families are often placed in the limelight of EMT regulation. However, all the while, several other TFs were repeatedly implicated in EMT processes [88]. This certainly applies to constituents of the AP-1 TF [59,74,89,90]. Consistent with previous studies, also our TFBM analyses predicted a prominent role for the AP-1 motif in TGFβ1-induced EMT and pinpointed JUN and JUNB as highly likely TFs operating through this motif. Given the contrasting outcome of the TFBM analyses for SNAIL1 and ZEB1 compared to AP-1, as well as prior knowledge about the functional importance of JUN and JUNB in EMT, we considered the inactivation of the *JUN* and *JUNB* genes as informative controls to further scrutinize the reliability of TFBM analyses. Indeed, in MCF10A cells, we confirmed the critical role of JUNB in EMT-related gene expression changes [59,90] and report the identification of several novel direct JUNB target genes. In addition, we demonstrated the importance of JUNB for bringing about major aspects of the EMT phenotype such as slowing down proliferation while enhancing migration and invasion [2,91,92], altogether confirming the importance of JUNB for TGFβ1-induced EMT of MCF10A cells as anticipated from the bioinformatic analyses.

While the phenotype of MCF10A cells with an inactivated *JUNB* gene completely fulfilled the expectations, knocking out *JUN* resulted in surprisingly mild changes in EMT-related gene expression, migration and invasion. Similarly, compared to the MCF10A cell background, TGFβ1-induced EMT was less severely compromised in A549 *JUNB* mut cells. JUN and JUNB belong to a large family of proteins forming AP-1 TF complexes [93]. As frequently encountered characteristics, members of such TF families may variably homo- and heterodimerize, recognize highly similar and even identical TFBMs, and demonstrate functional redundancy. These features, all of which are applicable to the AP-1 family [93,94], make it difficult to predict by computational means precisely which TF or which combination of TF family members actually function at a given TFBM and could explain the unexpected outcome of the *JUN* loss-off-function experiments and the cell-type-dependent severity of the *JUNB* mut phenotype. For example, consistent with the observed changes in the expression AP-1 family members in *JUN* and *JUNB* KO cell clones, it could be that JUNB or another AP-1 family member filled the void and substituted for JUN in MCF10A *JUN* KO cells. As an extension of this reasoning, cell-type-specific differences in the expression levels of AP-1 subunits in control and TGFβ1-treated conditions combined with functional interchangeability [94] might underlie TGFβ1-inducible but apparently JUNB-independent regulation of genes in A549 cells that had been validated as JUNB targets in MCF10A cells. Ultimately, to clarify these issues, comparative analyses of gene expression and genome-wide binding patterns of members of the AP-1 TF family in WT and KO cell clones from different cell lines will be necessary.

The results of our complementary loss-of-function and gain-of-function experiments demonstrated that JUNB was necessary and sufficient to trigger EMT in MCF10A cells. However, its importance for TGFβ1-induced EMT in A549 cells was much less pronounced. Therefore, in contrast with previous suggestions [59] we do not consider JUNB to be a master regulator which orchestrates EMT-associated transcriptional reprogramming all by itself and in a universal manner. Rather, the results of the integrated analyses of our RNA-seq and ATAC-seq experiments combined with the functional analyses in MCF10A and A549 cells indicate that JUNB is more likely to be embedded in larger, cell-type-specific gene regulatory networks that comprise additional transcription factors. The observation that inactivation of a single TF such as *JUNB* can strongly impair many, if not all phenotypic changes that accompany EMT, and that expression of several other TFs was deregulated in *JUNB* mut cell clones, hints at a hierarchical organization of these gene regulatory networks. Interestingly, JUNB appears to be situated at different levels of the hierarchy in MCF10A and A549 cells. Possibly, context-dependent changes in functional importance also apply to other TFs implicated in EMT. Accordingly, it will be of considerable interest to further determine which TFs govern EMT transcriptional regulation in different cellular settings and to examine the layout of the underlying gene regulatory circuits.

## 5. Conclusions

A major conclusion from our current study is that SNAIL proteins and ZEB1 are not strictly required for EMT, and therefore, absence of these TFs does not necessarily indicate absence of EMT. As an extension of this reasoning, even when SNAIL proteins and ZEB1 are found to be expressed, this likewise may not be a reliable indication of an ongoing EMT process. Furthermore, the observation that phenotypic changes characteristic for EMT can occur in the absence of SNAIL proteins and ZEB1, argues for plasticity of the underlying regulatory mechanisms. Apparently, EMT-associated transcriptional reprogramming can be brought about in a highly context-dependent fashion by different cohorts of TFs, including AP-1 family members and probably many others. From a technical perspective, we consider the integrated analyses of ATAC-seq and RNA-seq datasets by computational tools a powerful and versatile strategy to predict with high confidence transcriptional regulators of a biological process and their target genes. Nonetheless, the discrepancy between the roles of JUN and JUNB in TGFβ1-induced EMT as anticipated from IMAGE and as determined by loss-of-function experiments, may serve as a reminder that the output of computational analyses oftentimes are predictions that ideally should be complemented and corroborated by independent experimental approaches. Irrespective of this, the results of our study yield an extended and considerably more refined picture of the dynamic regulatory landscape of TGFβ1-induced EMT and provide a valuable resource for its further exploration.

## Data Availability

All ATAC-seq and RNA-seq data were deposited in the Gene Expression Omnibus under the accession numbers GSE210496, GSE210498, and GSE210499. All other data on which the results and conclusions of this study are based, are available from the corresponding author on reasonable request.

## Supplementary Materials

The following supporting information can be downloaded at: https://www.mdpi.com/2072-6694/15/2/558/s1. Figure S1. Percentages of closed and open differentially accessible regions (DARs) in MCF10A cells treated with TGFβ1 compared to untreated control samples. Figure S2. Percentages of differentially expressed genes (DEGs) up- and downregulated in MCF10A cells treated with TGFβ1 compared to untreated control samples. Figure S3. TGFβ1 induces dynamic changes in chromatin accessibility and gene expression indicative of EMT. Figure S4. Enrichment analysis of GO terms “Biological Processes” for DEGs highly correlated with proximal DARs and distal DARs. Figure S5. Analyses of transcription factor binding motif activity suggests a prominent function for AP-1 family members JUN and JUNB in TGFβ1-induced EMT. Figure S6. SNAIL2 performs TGFβ1-independent functions regulating the basal state of MCF10A cells. Figure S7. The combined inactivation of *SNAI1* and *ZEB1* does not prevent EMT. Figure S8. Morphological changes of MCF10A *SNAI1* and *ZEB1* KO cell clones in response to TGFβ1. Figure S9. Inactivation of *SNAI1* and *ZEB1* does not impair TGFβ1-induced migration and invasion. Figure S10. TGFβ1-induced migration and invasion are not abolished by dual inactivation of *SNAI1* and *ZEB1*. Figure S11. Morphological changes of MCF10A *JUN* and *JUNB* WT and KO/mutant cell clones in response to TGFβ1. Figure S12. Differential impact of *JUN* and *JUNB* inactivation on TGFβ-1-induced EMT. Figure S13. Expression analysis of EMT marker genes and EMT-TFs after TGFβ1 treatment of MCF10A *JUN* and *JUNB* WT and KO/mut cell clones. Figure S14. Inactivation of *JUN* and *JUNB* impairs TGFβ1-induced migration and invasion. Figure S15. Inactivation of *JUNB* has a comparatively mild effect on TGFβ1-induced EMT of A549 cells. Figure S16. Overexpression of *JUNB* does not elicit EMT in A549 cells. Figure S17. JUNB is a major determinant of gene expression in treatment-naïve and TGFβ1-stimulated MCF10A cells. Figure S18. *JUNB* inactivation alters the expression of some AP-1 family members. Figure S19. Effects of *JUN* and *JUNB* inactivation on the expression of predicted *JUNB* target genes during TGFβ1-induced EMT. Figure S20. Activation of TGFβ1-regulated and JUNB-dependent genes by JUNB-ER in MCF10A cells. Figure S21. Components of the TGFβ-regulated EMT signature and *JUNB* can be activated by TGFβ1 in immortalized human mammary epithelial cells (IMECs), the lung adenocarcinoma cell line A549, and the pancreatic cancer cell line PANC1. Figure S22. JUNB-independent regulation of selected TGFβ1-inducible genes in A549 cells. Figure S23. Compilation of uncropped immunoblots for all figures and quantification of proteins analyzed. Table S1: List of cell lines and their culture conditions. Table S2: Sequences of oligonucleotides and their application. Table S3: List of antibodies and their applications. Table S4: List summarizing the genomic status of MCF10A and A549 cell clones derived from CRISPR/Cas9 genome-editing experiments. Table S5: List of sgRNA target sequences used in CRISRP/Cas9 experiments. Table S6. Annotated DARs in MCF10A treated with TGFβ1 compared to solvent-treatment. Table S7. DEGs in MCF10A cells treated with TGFβ1 compared to solvent-treated samples. Table S8. GO terms “Biological Processes” enriched among DEGs of highly correlated proximal DARs and DEGs. Table S9. GO terms “Biological Processes” enriched among DEGs of highly correlated distal DARs and DEGs. Table S10 Transcription factor (TF) motif activity changes at proximal and distal DARs and predicted target gene expression determined by IMAGE. Table S11: DEGs in JUNB mut clones compared to JUNB WT clones.
