# Correction: Antón-García et al. TGFβ1-Induced EMT in the MCF10A Mammary Epithelial Cell Line Model Is Executed Independently of SNAIL1 and ZEB1 but Relies on JUNB-Coordinated Transcriptional Regulation. *Cancers* 2023, *15*, 588

**DOI:** 10.3390/cancers16030509

**Published:** 2024-01-24

**Authors:** Pablo Antón-García, Elham Bavafaye Haghighi, Katja Rose, Georg Vladimirov, Melanie Boerries, Andreas Hecht

**Affiliations:** 1 Institute of Molecular Medicine and Cell Research, Faculty of Medicine, University of Freiburg, 79104 Freiburg, Germany; pablo.anton.garcia@mol-med.uni-freiburg.de (P.A.-G.); katja.rose@mol-med.uni-freiburg.de (K.R.); georgvladimirov@web.de (G.V.); 2 Faculty of Biology, University of Freiburg, 79104 Freiburg, Germany; 3 Institute of Medical Bioinformatics and Systems Medicine, Medical Center–University of Freiburg, Faculty of Medicine, University of Freiburg, 79106 Freiburg, Germany; elham.bavafaye.haghighi@uniklinik-freiburg.de (E.B.H.); melanie.boerries@uniklinik-freiburg.de (M.B.); 4 German Cancer Consortium (DKTK), Partner Site Freiburg, German Cancer Research Center (DKFZ), 69120 Heidelberg, Germany; 5 BIOSS Centre for Biological Signalling Studies, University of Freiburg, 79104 Freiburg, Germany

In the original publication [1], there was a mistake in Figure 6 as published. In Figure 6c, the image showing control cells treated with EtOH (CTRL/EtOH) was inadvertently duplicated and used to also display control cells treated with 4-OHT (CTRL/4-OHT), which is not correct. The corrected Figure 6 in which the CTRL/4-OHT micrograph of panel c was replaced appears below.

The authors state that the scientific conclusions are unaffected. This correction was approved by the Academic Editor. The original publication has also been updated.

## Figures and Tables

**Figure 6:**
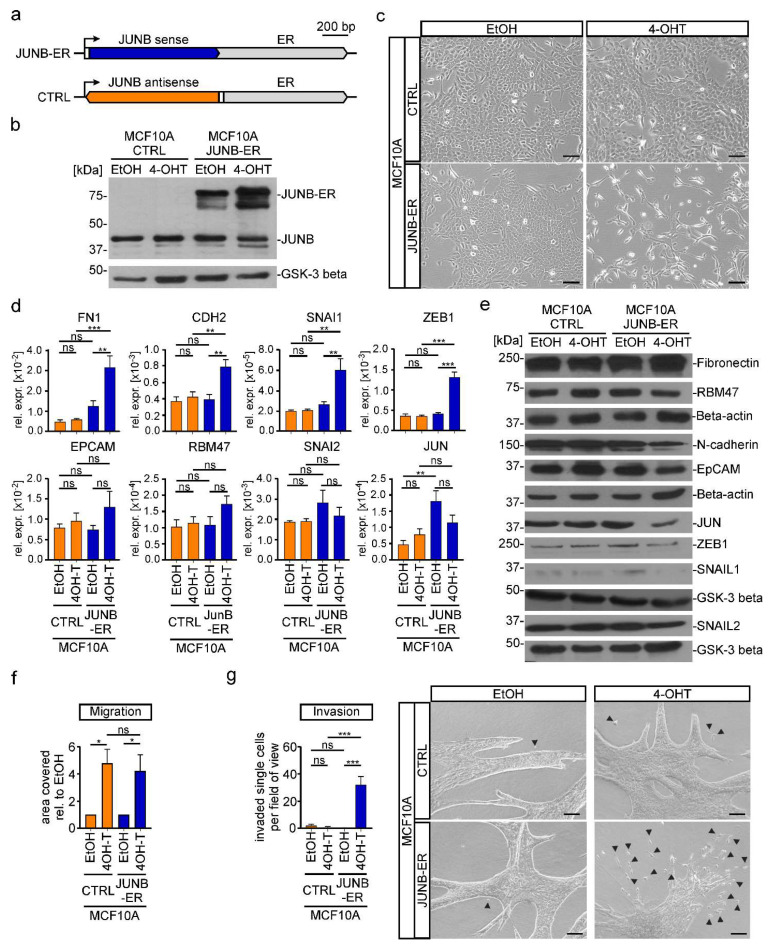
Increased JUNB activity is sufficient to induce EMT in MCF10A cells. (**a**) Schematic representation of the gene expression cassette for a fusion protein consisting of the human *JUNB* coding region (JUNB sense) and a mutant estrogen receptor hormone binding domain (ER). A control construct (CTRL) harbored the JUNB coding region in the opposite orientation (JUNB antisense). The angled arrow indicates the TSS; the white box represents the 5′-untranslated region. (**b**) Simultaneous detection of endogenous JUNB and ectopic JUNB-ER expression by Western blot using nuclear extracts from MCF10A cells that had been stably transduced with a retroviral vector for the expression of JUNB-ER or the control construct. Glycogen synthase kinase-3 beta (GSK-3 beta) was used as the loading control. Molecular weights are given in kilodaltons (kDa). One representative result from three independent biological replicates is presented. (**c**) Representative phase-contrast microscopy pictures from one of three independent biological replicates showing the indicated MCF10A *JUNB-ER* and CTRL cells. The scale bar represents 200 µm. (**d**) Gene expression analysis of epithelial and mesenchymal marker genes in MCF10A *JUNB-ER* and CTRL cells. RNA levels were measured by qRT-PCR and are shown as relative expression compared to those of *GAPDH*. (**e**) Detection of epithelial and mesenchymal markers in the cytoplasmic (Fibronectin, N-cadherin, EpCAM, RBM47) and nuclear fractions (SNAIL1, SNAIL2, ZEB1, JUN) of protein lysates from MCF10A *JUNB-ER* and CTRL cells. Beta-actin and GSK-3beta were used as loading controls. Molecular weights are given in kilodaltons (kDa). Representative results from one of three independent biological replicates are shown. (**f**) Results from transwell migration assays performed with MCF10A *JUNB-ER* or CTRL cells. Depicted is the area covered by cells on the bottom surface of transwell inserts relative to the value of EtOH treated cells. (**g**) Spheroid invasion assays performed with cellular aggregates embedded in a collagen I matrix. For the quantification single cells and small aggregates (exemplarily marked by arrow heads) that had detached from the bulk of the cell aggregates were counted in two fields of view per cell line and condition. Pictures of representative spheroids from one out of three independent biological replicates are shown on the right. The scale bars represent 100 μm. (**b**,**e**) Uncropped versions of immunoblots including densitometry readings can be found in Figure S23. (**b**–**g**) For all experiments, cells were treated with 100 nM 4-OHT or a corresponding volume of ethanol (EtOH) for 72 h prior to harvest. (**d**,**f**,**g**) Bars represent the mean values from at least three independent biological replicates. Error bars depict the standard error of the mean. Stars indicate *p*-values corrected for multiple testing by the FDR method. *: FDR < 0.05, **: FDR < 0.01, ***: FDR < 0.001, ns: not significant; one-way ANOVA.
