# Supplementary material for: TGFβ1-Induced EMT in the MCF10A Mammary Epithelial Cell Line Model Is Executed Independently of SNAIL1 and ZEB1 but Relies on JUNB-Coordinated Transcriptional Regulation
Source: Cancers (Basel). 2023 Jan 16;15(2):558. doi: 10.3390/cancers15020558 (PMC9856774; doi:10.3390/cancers15020558)
Supplement: Supplementary file 1 [file cancers-15-00558-s001.zip › cancers-1876179-Suppl_Mat_proofread.pdf]

Supplementary Material: TGFβ1-Induced EMT in the MCF10A Mammary Epithelial Cell Line Model Is Executed Independently of SNAIL1 and ZEB1 but Relies on JUNB-Coordinated Transcriptional Regulation

Pablo Antón-García, Elham Bavafaye Haghighi, Katja Rose, Georg Vladimirov, Melanie Boerries and Andreas Hecht

This document contains Figures S1 – S23 and Supplementary Tables S1 – S5

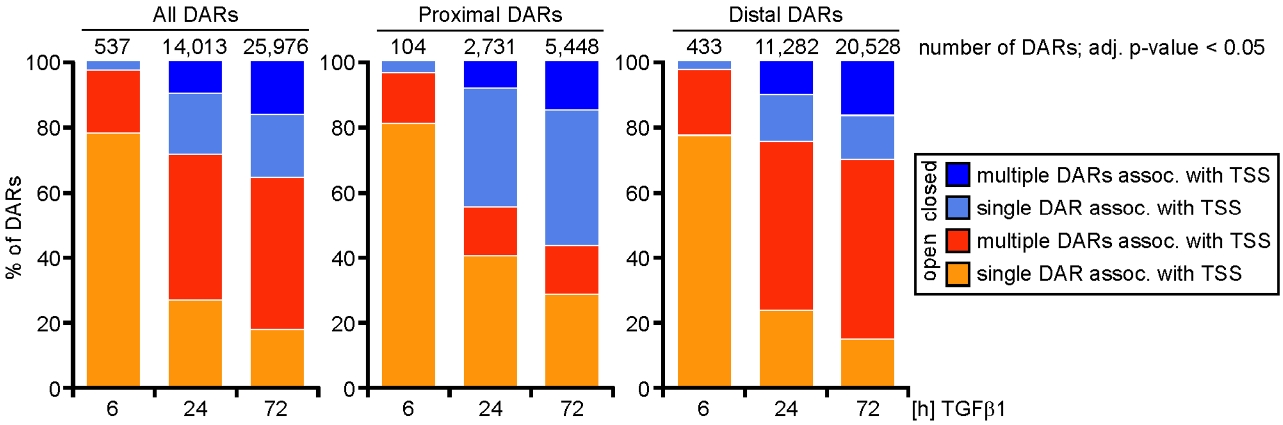

Figure S1.: Percentages of closed and open differentially accessible regions (DARs) in MCF10A cells treated with TGFβ1 compared to untreated control samples. The analyses were done for all DARs together and after segregation into promoter-proximal and promoter-distal DARs. Open and closed DARs were further stratified depending upon whether multiple DARs or a single DAR were associated (assoc.) with a given TSS. The numbers on top of each bar indicate the total number of DARs at each time point of TGFβ1 treatment.

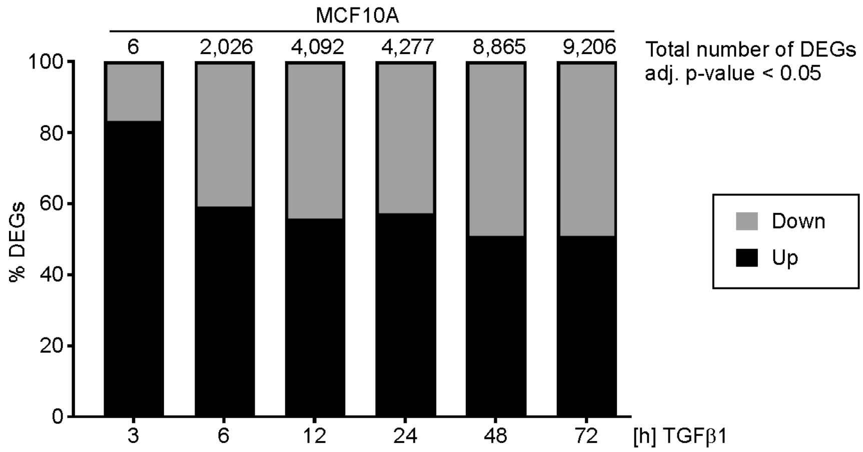

Figure S2.: Percentages of differentially expressed genes (DEGs) up- and downregulated in MCF10A cells treated with TGFβ1 compared to untreated control samples. The numbers on top of each bar indicate the total number of DEGs at each time point of TGFβ1 treatment compared to solvent-treated control samples.

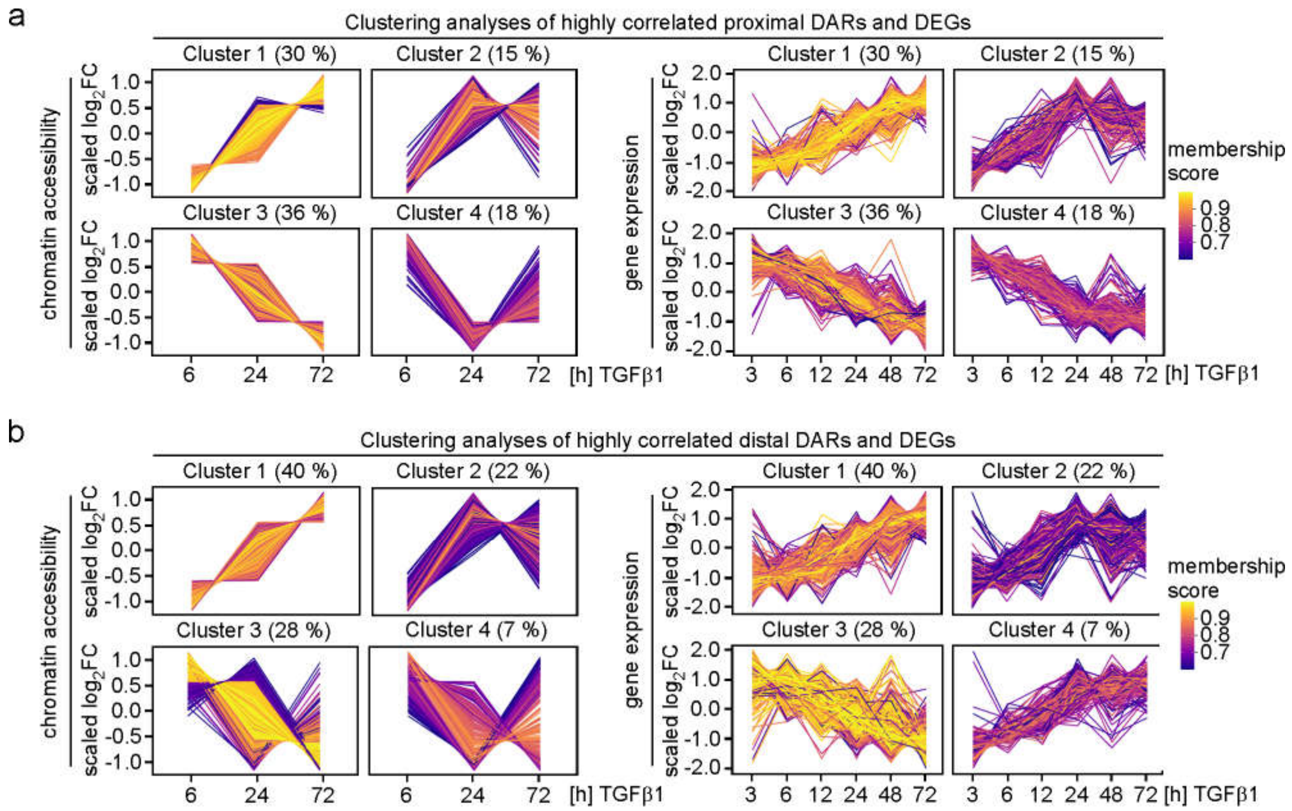

Figure S3.: TGFβ1 induces dynamic and coordinated changes in chromatin accessibility and gene expression. (**a, b**) Clustering analyses of time series data of highly correlated proximal DARs and DEGs (**a**) and highly correlated distal DARs and DEGs (**b**). Color code indicates cluster membership scores defined as the distance of each cluster member to the centroid of the cluster.

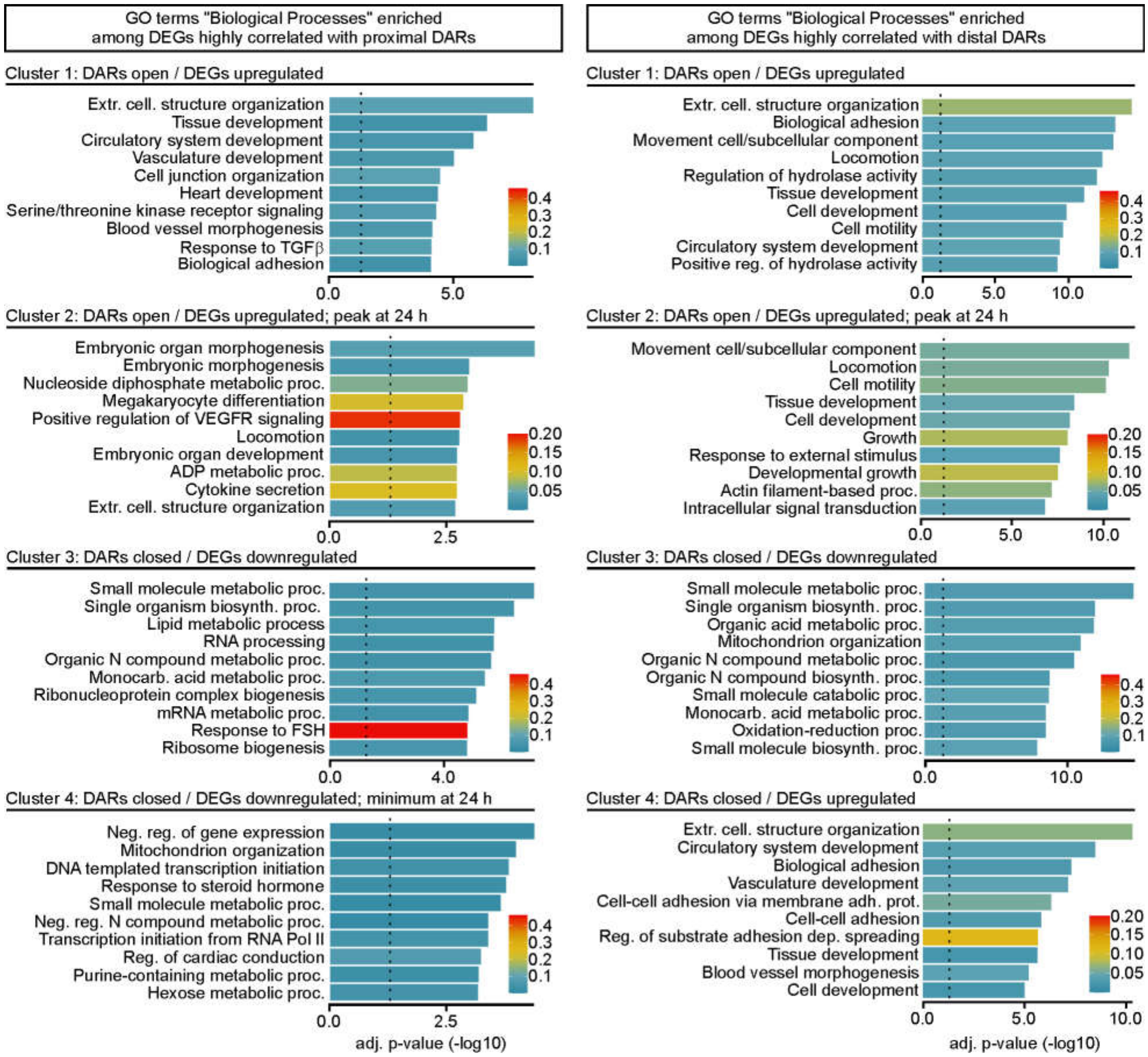

Figure S4.: Enrichment analysis of GO terms "Biological Processes" for DEGs highly correlated with proximal DARs (left) and distal DARs (right). Bar plots show the top ten enriched GO terms. Bar colors indicate the gene ratios. The length of the bars specifies statistical significance.

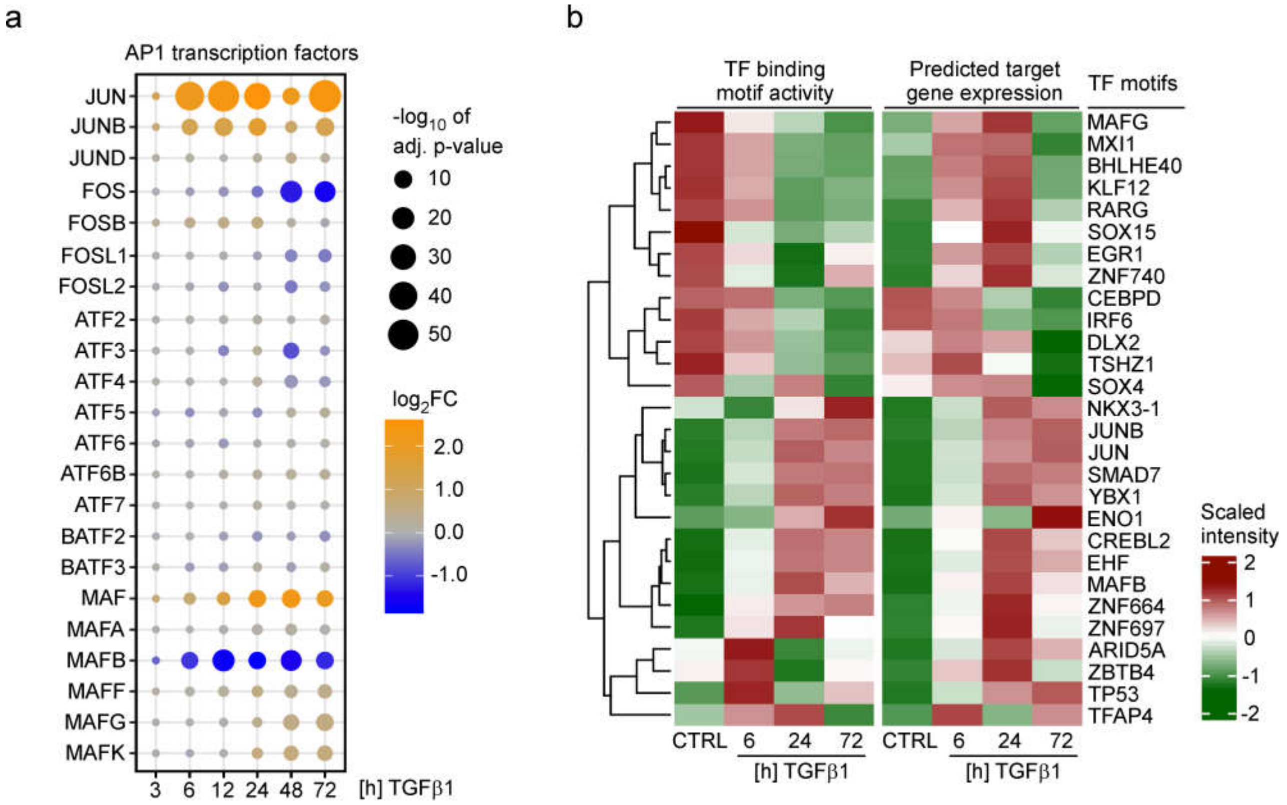

Figure S5.: Analyses of transcription factor binding motif activity suggests a prominent function for AP-1 family members JUN and JUNB in TGFβ1-induced EMT. (**a**) Changes in gene expression of all members of the AP-1 TF family extracted from RNA-seq data. The dot size indicates statistical significance as −log of the adj. *p*-value. The color of the dots corresponds to the scaled $\log_2$FC for each time point of TGFβ1 treatment compared to CTRL samples. (**b**) The heatmap displays significant changes in the activity of 28 TFBMs common to proximal and distal DARs as well as average expression of predicted targets during the TGFβ1 time course treatment. Distinct types of regulatory relationships are apparent: Declining TFBM activities can be correlated with increased and decreased expression of predicted target genes which might indicate the loss of activity of transcriptional repressors and activators, respectively. Instances where TFBM activity and target gene expression increased in parallel, most likely identify cases of TGFβ1-mediated upregulation of TFs and their downstream targets.

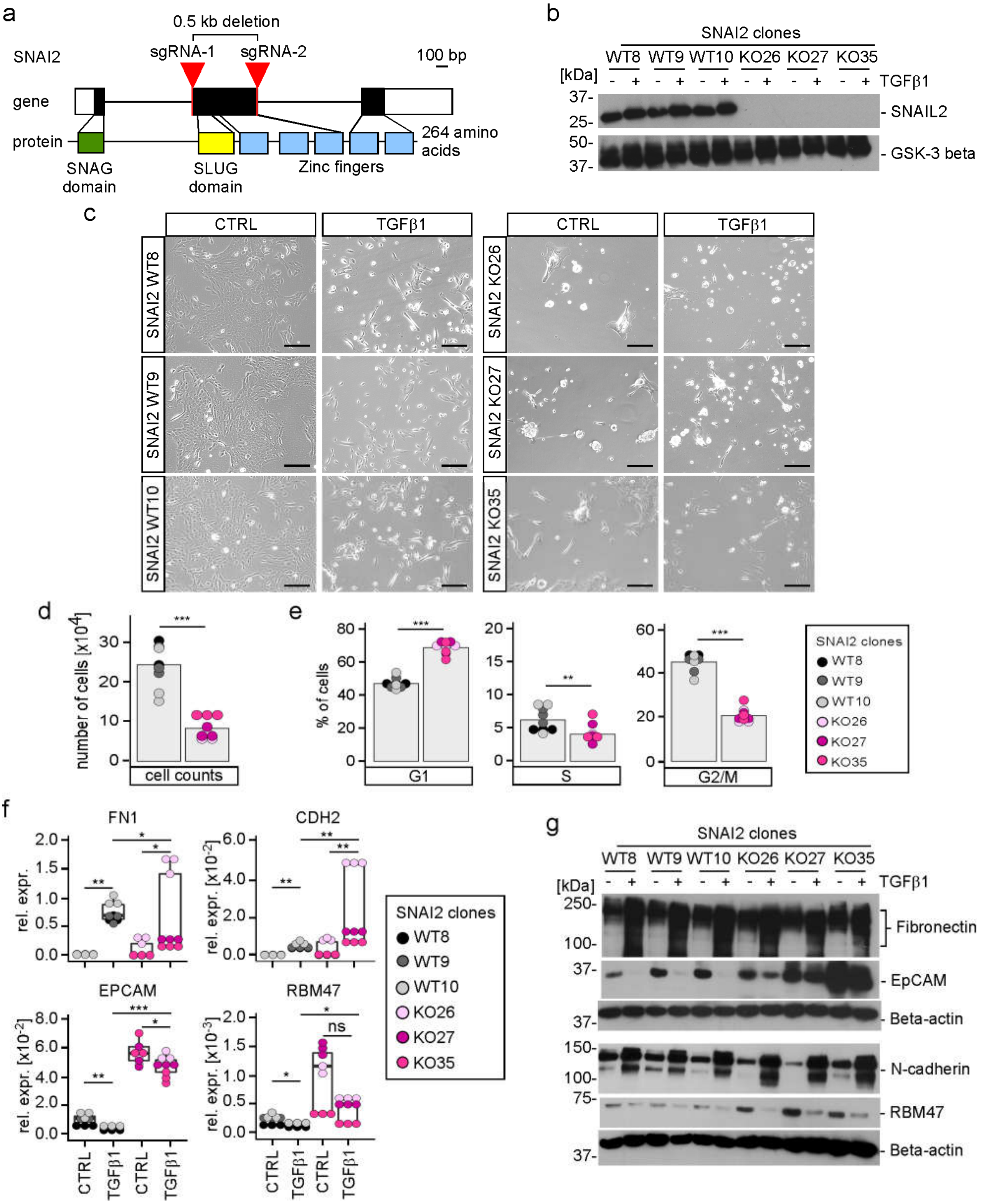

Figure S6.: SNAIL2 performs TGFβ1-independent functions regulating the basal state of MCF10A cells. (**a**) Schematic representation of the human *SNAI2* gene and protein. Exons are depicted as boxes with protein-coding parts colored in black and UTRs in white. Relevant functional domains of the protein are indicated by color and linked to the exonic regions by which they are encoded. The location of sgRNA targets is marked by red arrows. Exons are drawn to scale. (**b**) Detection of SNAIL2 by western blot using nuclear extracts from MCF10A *SNAI2* WT and KO cell clones. GSK-3 beta was used as loading control. Molecular weights are given in kilodaltons (kDa). One representative result out of three independent biological replicates is presented. (**c**) Representative phase-contrast microscopy pictures from one of three independent biological replicates showing the indicated *SNAI2* WT and KO clones after 72 hours of TGFβ1 treatment. The scale bar represents 200 μm. (**d**) Cell counts from *SNAI2* WT and KO clones 48 hours after seeding equal starting numbers of

cells for each cell clone. (**e**) The proportion of *SNAI2* WT and KO clones in G1, S, and G2/M phases of the cell cycle, analyzed by flow cytometry. (**f**) Targeted expression analyses of epithelial and mesenchymal markers after 72 hours of TGFβ1 treatment. RNA levels were measured by qRT-PCR and are shown as relative expression compared to the RNA levels of *GAPDH*. Box plots summarize qRT-PCR results with each dot representing a single measurement. Stars indicate *p*-values corrected for multiple testing by the false discovery rate (FDR) method. *: FDR < 0.05, **: FDR < 0.01, ***: FDR < 0.001, ns: not significant; Mann-Whitney *U* test. (**g**) Western blot detection of epithelial and mesenchymal markers after 72 hours of TGFβ1 treatment by using the cytoplasmic fractions of protein lysates from the *SNAI2* WT and KO cell clones indicated. Beta-actin was used as the loading control. Molecular weights are given in kilodaltons (kDa). Representative results from one of two independent biological replicates are shown. In the bar plots from panels (d) and (e), the height of the bars represents mean values, while each dot represents the result of one of three independent biological replicates. Dot color in panels (d), (e), and (f) identifies the different cell clones. Uncropped versions of immunoblots from panels (b) and (g) including densitometry readings can be found in Figure S23.

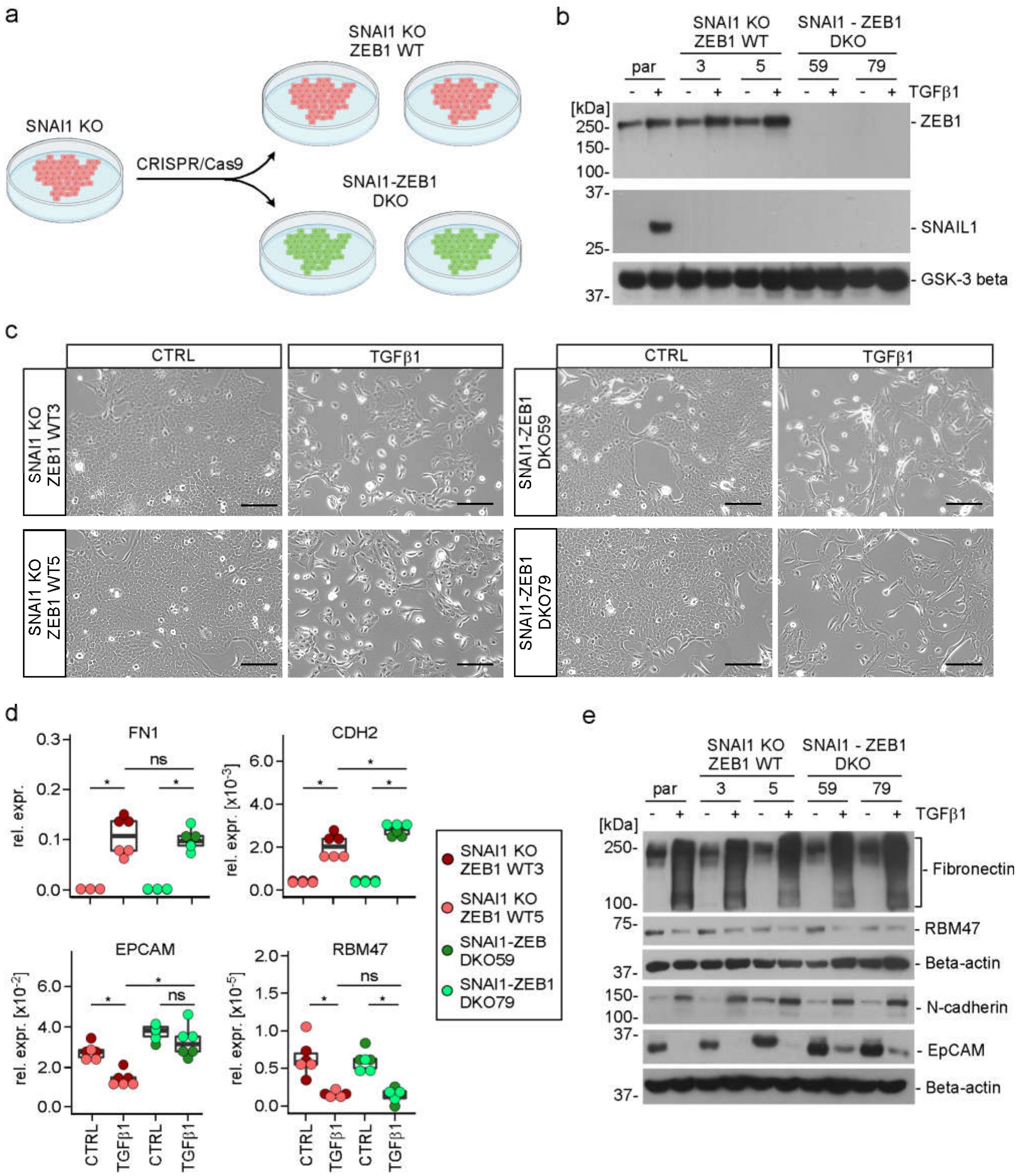

Figure S7.: The combined inactivation of *SNAI1* and *ZEB1* does not prevent EMT. (**a**) Experimental strategy to generate *SNAI1-ZEB1* double KO cells (DKO). By applying the CRISPR/Cas9 system to MCF10A *SNAI1* KO clone 112, *SNAI1-ZEB1* DKO clones were obtained. In parallel, *SNAI1* KO; *ZEB1* WT cell clones were retrieved and served as controls to assess the consequences of *ZEB1* inactivation in cells having undergone the same experimental procedure. (**b**) Detection of SNAIL1 and ZEB1 protein expression by western blotting using nuclear extracts from MCF10A *SNAI1* KO; *ZEB1* WT and *SNAI1-ZEB1 DKO* cells. The parental (par) MCF10A cell line was used as a positive control for the detection of SNAIL1. Glycogen synthase kinase-3 beta (GSK-3 beta) was used as the loading control. Molecular weights are given in kilodaltons (kDa). One representative result from three independent biological replicates is shown. (**c**) Representative phase-contrast microscopy pictures from one of three independent biological replicates showing the indicated MCF10A *SNAI1* KO; *ZEB1* WT and *SNAI1-ZEB1 DKO* cell clones after 72 hours of TGFβ1 treatment. The scale bar represents 200 μm. (**d**) Targeted gene expression analysis of epithelial and mesenchymal markers in the

indicated MCF10A *SNAI1* KO; *ZEB1* WT and *SNAI1-ZEB1 DKO* cell clones after 72 hours of TGFβ1 treatment. RNA levels were measured by qRT-PCR and are shown as relative expression (rel. expr.) compared to the RNA levels of *GAPDH*. Each dot in the box plots represents the result of a single measurement. Dot color identifies the cell clones. Stars indicate *p*-values corrected for multiple testing by the false discovery rate (FDR) method. *: FDR < 0.05, ns: not significant; Mann-Whitney *U* test. (**e**) Western blot detection of epithelial and mesenchymal markers after 72 hours of TGFβ1 treatment using the cytoplasmic fractions of protein lysates from parental (par) MCF10A cells and MCF10A *SNAI1* KO; *ZEB1* WT and *SNAI1-ZEB1 DKO* cell clones. Beta-actin was used as loading control. Molecular weights are given in kilodaltons (kDa). Representative results from one of two independent biological replicates are shown. Uncropped versions of immunoblots from panels (b) and (e) including densitometry readings can be found in Figure S23.

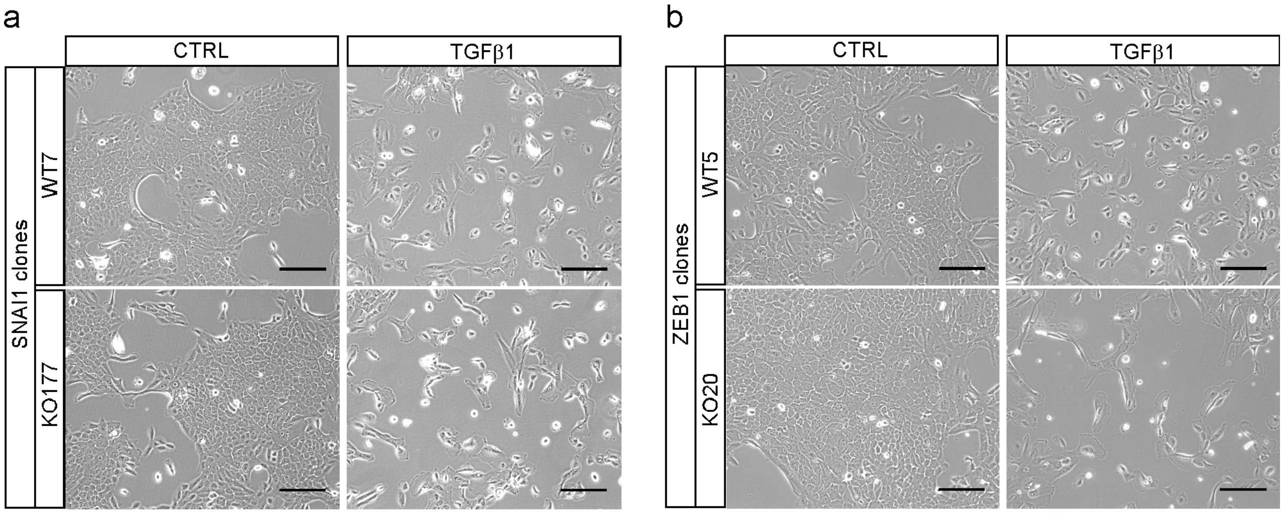

Figure S8.: Morphological changes of MCF10A *SNAI1* and *ZEB1* KO cell clones in response to TGFβ1. (**a**, **b**) Representative phase-contrast microscopy images from one of three independent biological replicates showing the *SNAI1* (a) and *ZEB1* (b) WT and KO cell clones indicated after 72 hours of TGFβ1 treatment. The scale bar represents 200 μm.

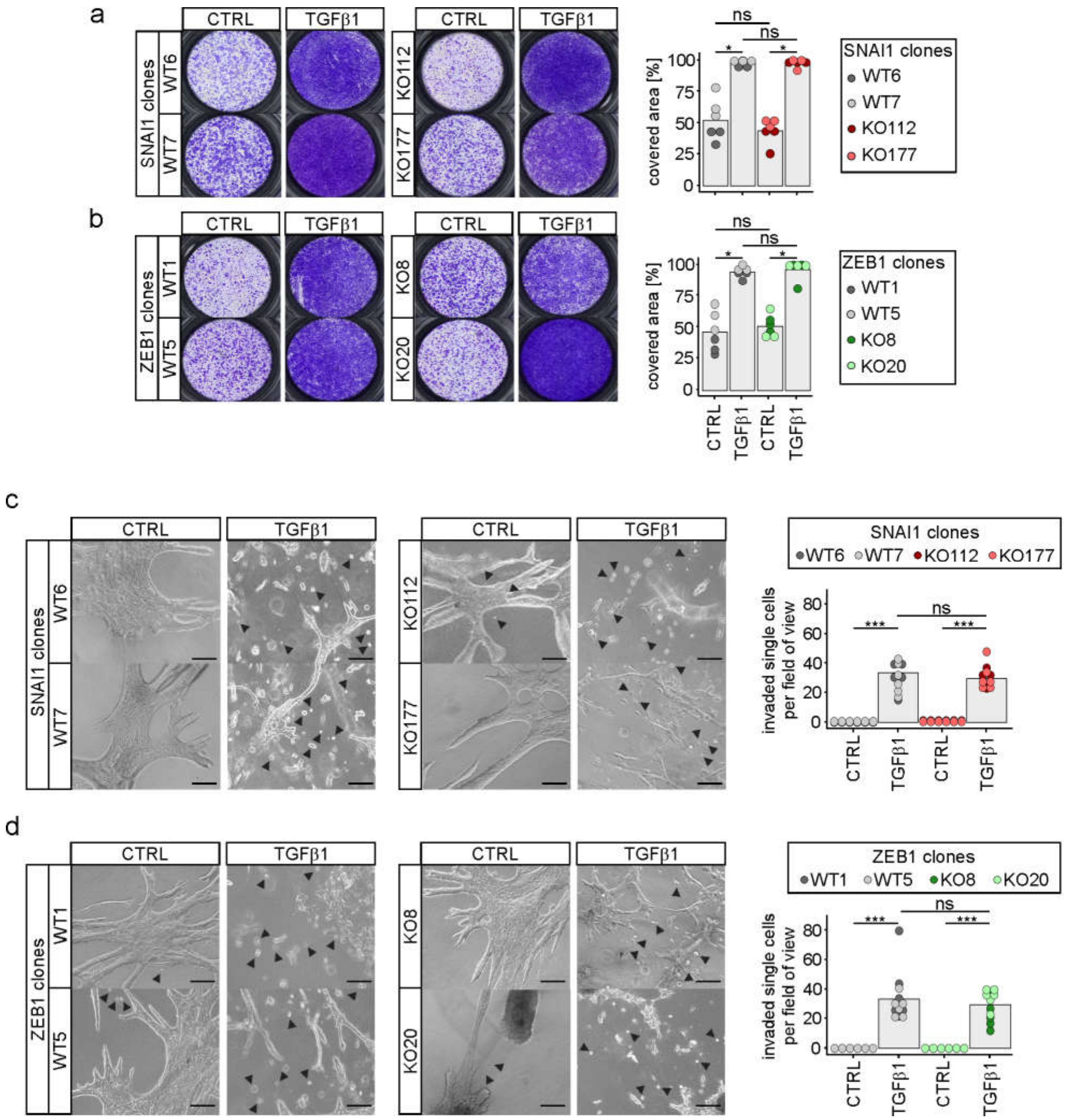

Figure S9.: Inactivation of *SNAI1* and *ZEB1* does not impair TGFβ1-induced migration and invasion. (**a, b**) The migratory phenotype of MCF10A *SNAI1* (a) and *ZEB1* (b) WT and KO cell clones treated with TGFβ1 for 72 h was measured in a transwell migration assay. Images on the left show crystal violet stainings from one out of three independent biological replicates. The quantifications of the migration assays are shown on the right, depicting the percentage of transwell area covered by migrated cells. (**c, d**) Spheroid invasion assays performed with MCF10A *SNAI1* and *ZEB1* WT and KO cell clones. Spheroids were embedded in a collagen I matrix and treated with TGFβ1 for 72 h. Pictures of representative cell aggregates from one out of three independent biological replicates are shown. For quantification of the invasion assays shown on the right, single cells that had detached from the bulk of the cell aggregates (exemplarily marked by arrow heads) were counted. The scale bars represent 100 µm. (a-d) The height of the bars indicates mean values. Each dot represents the result of one out of three independent biological replicates. The color code identifies the cell clones. Stars indicate p-values corrected for multiple testing by the false discovery rate (FDR) method. *: FDR < 0.05, ***: FDR < 0.001, ns: not significant; Mann-Whitney U test.

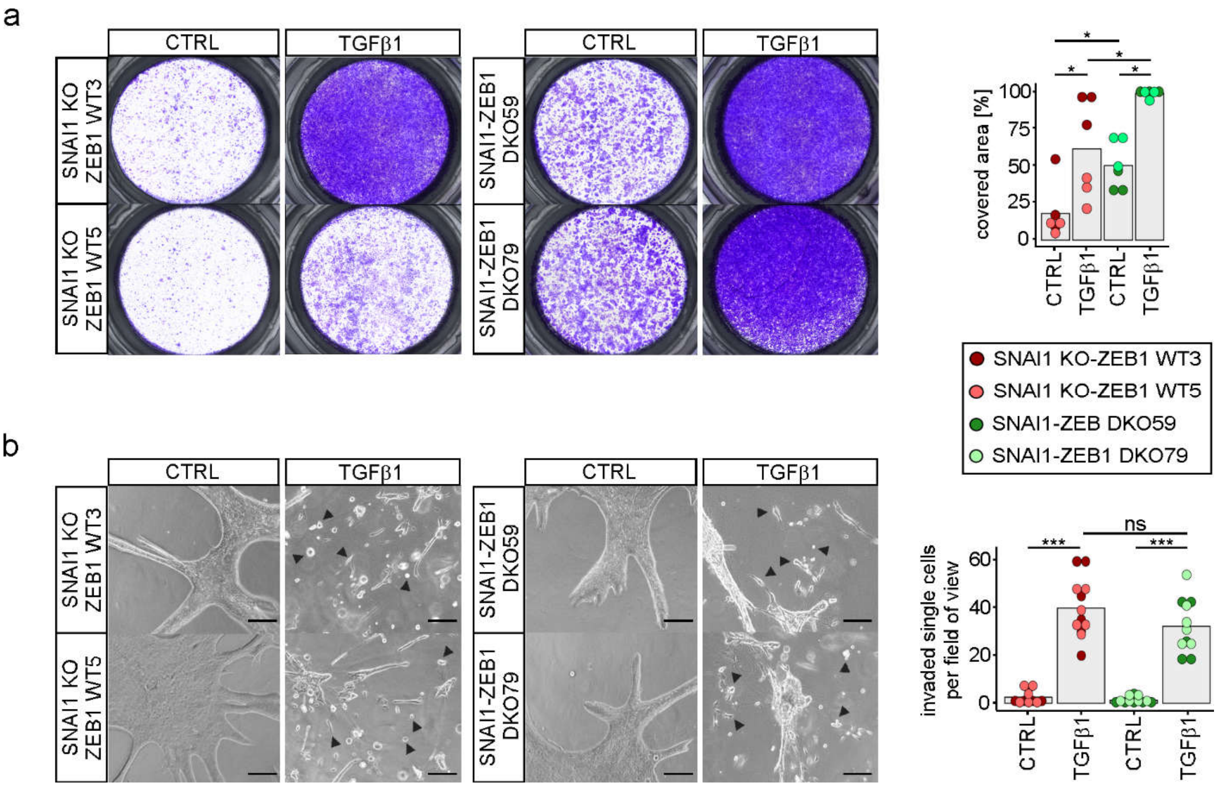

Figure S10.: TGFβ1-induced migration and invasion are not abolished by dual inactivation of *SNAI1* and *ZEB1*. (**a**) The migratory phenotype of MCF10A *SNAI1* KO; *ZEB1* WT and *SNAI1-ZEB1* DKO cell clones treated with TGFβ1 for 72 h was measured in a transwell migration assay. Images on the left show crystal violet stainings from one out of three independent biological replicates. The quantifications of the migration assays are shown on the right, depicting the percentage of transwell area covered by migrated cells. (**b**) Spheroid invasion assays performed with MCF10A *SNAI1* KO; *ZEB1* WT and *SNAI1-ZEB1* DKO cell clones. Spheroids were embedded in a collagen I matrix and treated with TGFβ1 for 72 h. Pictures of representative cell aggregates from one out of three independent biological replicates are shown. For quantification of the invasion assays shown on the right, single cells (exemplarily marked by arrow heads) that had detached from the bulk of the cell aggregates were counted. The scale bars represent 100 µm. (a, b) The height of the bar indicates mean values. Each dot represents the result of one out of three independent biological replicates. The color code identifies the cell clones. Stars indicate *p*-values corrected for multiple testing by the false discovery rate (FDR) method. *: FDR < 0.05, ***: FDR < 0.001, ns: not significant; Mann-Whitney *U* test.

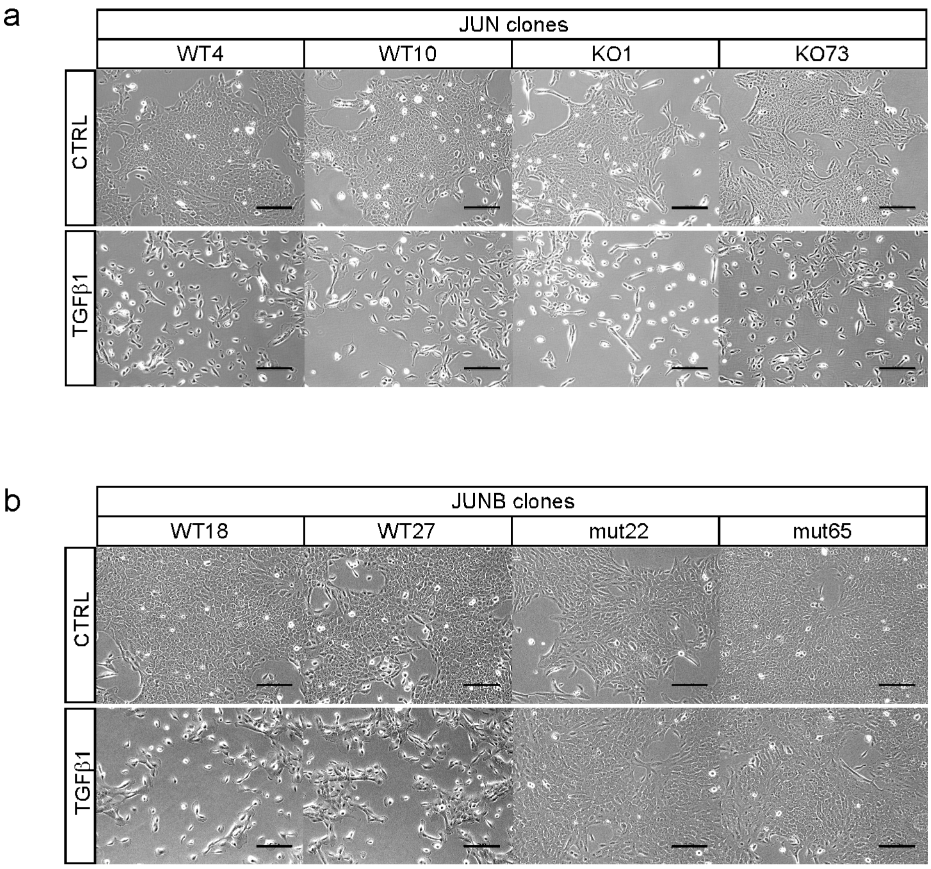

Figure S11.: Morphological changes of MCF10A *JUN* and *JUNB* WT and KO/mutant cell clones in response to TGFβ1. (**a, b**) Representative phase-contrast microscopy images from one of three independent biological replicates showing the *JUN* (a) and *JUNB* (b) WT and KO/mut cell clones indicated after 72 hours of TGFβ1 treatment. The scale bar represents 200 μm.

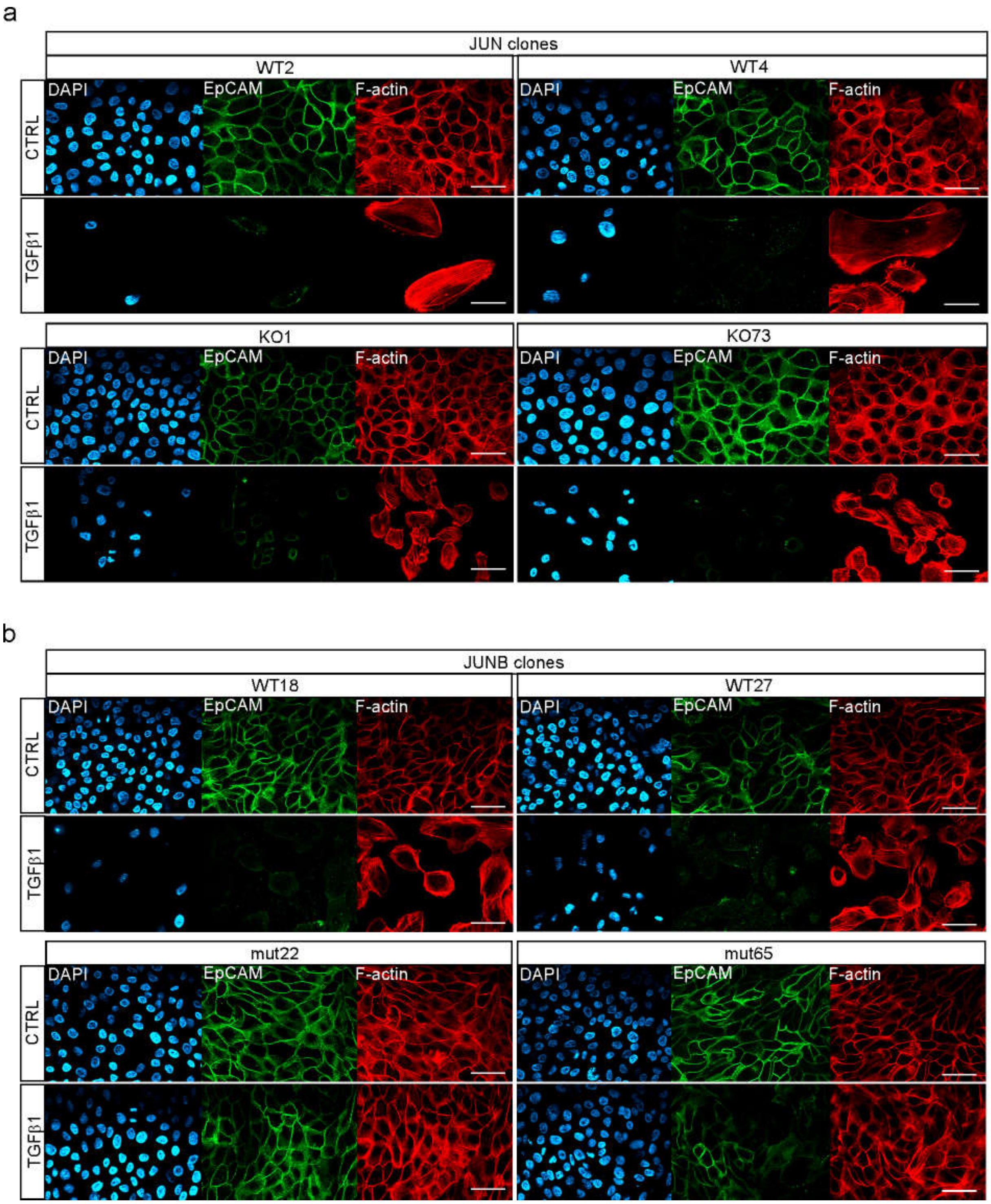

Figure S12.: Differential impact of *JUN* and *JUNB* inactivation on TGFβ1-induced EMT. (**a, b**) EpCAM expression and intracellular localization as well as F-actin intracellular distribution were analyzed by immunofluorescence staining and decoration with Alexa Fluor 555 phalloidin, respectively, for the MCF10A *JUN* (a) and *JUNB* (b) WT and KO/mut cell clones indicated. Representative images from one out of three independent biological replicates are shown. DAPI was used to stain nuclei of cells. The scale bar depicts 50 μm.

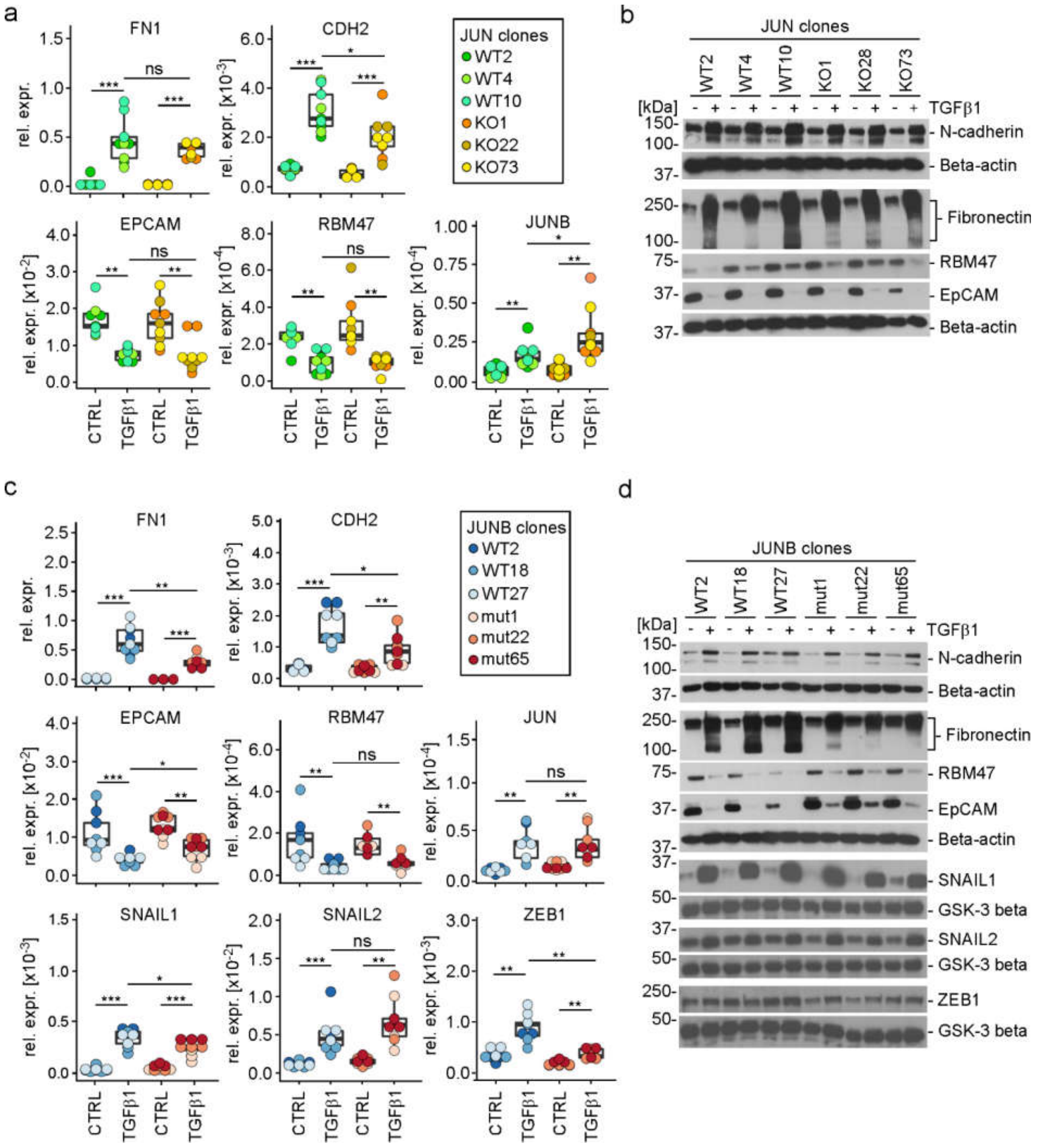

Figure S13.: Expression analysis of EMT marker genes and EMT-TFs after TGFβ1 treatment of MCF10A *JUN* and *JUNB* WT and KO/mut cell clones. (**a, c**) Targeted gene expression analyses of epithelial and mesenchymal markers in MCF10A *JUN* (a) and *JUNB* (c) WT and KO/mut cell clones after 72 h of TGFβ1 treatment. In *JUNB* WT and mut cell clones additionally expression of EMT-TFs was analyzed. RNA levels were measured by qRT-PCR and are given as relative expression compared to the RNA levels of *GAPDH*. Box plots summarize the qRT-PCR results. Each dot represents the result of a single measurement. Dot color identifies the cell clones. Stars indicate *p*-values corrected for multiple testing by the FDR method. *: FDR < 0.05, **: FDR < 0.01, ***: FDR < 0.001, ns: not significant; Mann-Whitney *U* test. (**b, d**) Western blot detection of epithelial and mesenchymal markers by using the cytoplasmic fraction of protein lysates from MCF10A *JUN* and *JUNB* WT and KO/mut cell clones as indicated. Expression of EMT-TFs was additionally analyzed in *JUNB* WT and mut cell clones using nuclear extracts. Beta-actin and GSK-3 beta were used as the loading controls as indicated. Molecular weights are given in kDa. Representative results from one of three independent biological replicates are shown. Uncropped versions of immunoblots including densitometry readings can be found in Figure S23.

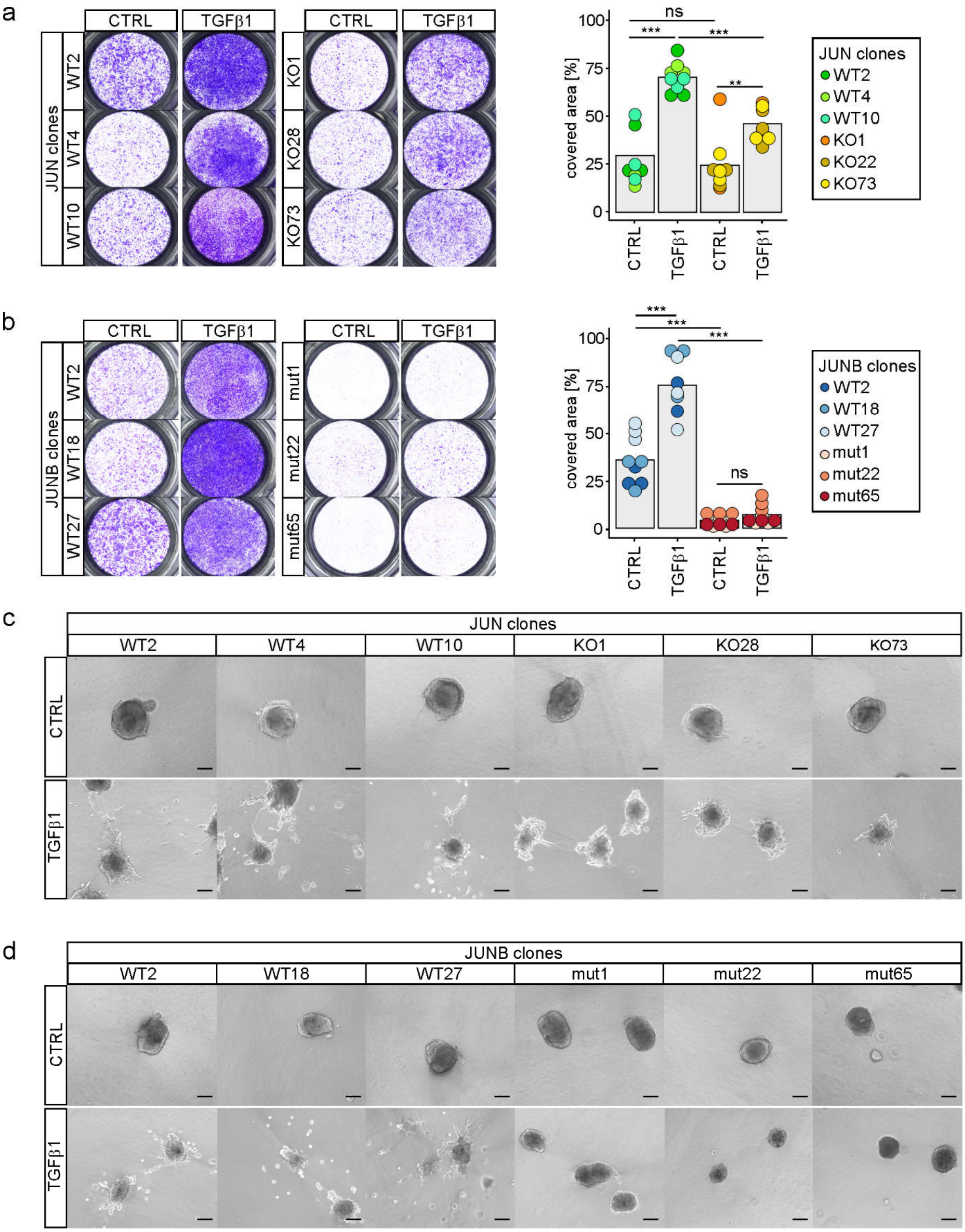

Figure S14.: Inactivation of JUN and JUNB impairs TGFβ1-induced migration and invasion. (**a, b**) The migratory phenotype of MCF10A *JUN* (a) and *JUNB* (b) WT and KO/mut cell clones treated with TGFβ1 for 72 h was measured in a transwell migration assay. Images on the left show crystal violet stainings from one out of three independent biological replicates. The quantifications of the migration assays are shown on the right, depicting the percentage of transwell area covered by migrated cells. The height of the bars indicates mean values. Each dot represents the result of one out of three independent biological replicates. The color code identifies the cell clones. Stars indicate p-values corrected for multiple testing by the false discovery rate (FDR) method. **: FDR < 0.01, ***: FDR < 0.001, ns: not significant; Mann-Whitney *U* test. (**c, d**) Spheroid invasion assays performed with MCF10A *JUN* and *JUNB* WT and KO/mut cell clones. Spheroids were embedded in a collagen I

matrix and treated with TGFβ1 for 72 h. Pictures of representative spheroids from one out of three independent biological replicates are shown. The scale bars represent 100 μm.

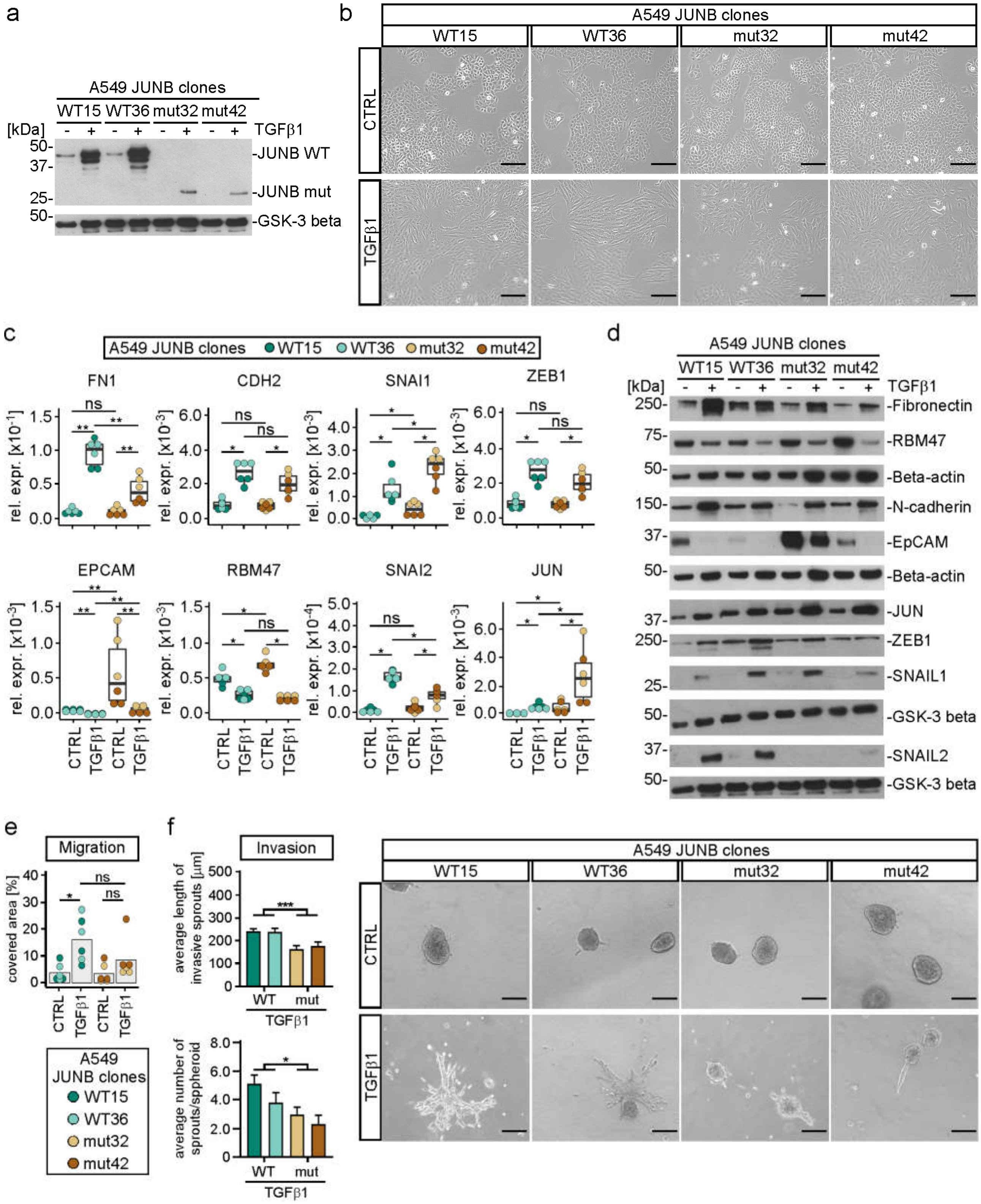

Figure S15.: Inactivation of JUNB has a comparatively mild effect on TGFβ1-induced EMT of A549 cells. (**a**) Detection of JUNB by western blot using nuclear extracts from A549 wild type (WT) and mutant (mut) cell clones. One representative result from three independent biological replicates is shown. Uncropped versions of immunoblots including densitometry readings can be found in Figure S23. (**b**) Representative phase-contrast microscopy pictures from one of three independent biological replicates showing the indicated A549 *JUNB* WT and mut cell clones. The scale bars depict 200 μm. (**c**) Targeted gene expression analyses of epithelial and mesenchymal markers in A549 *JUNB* WT and mut cell clones. RNA levels were measured by qRT-PCR and are given as relative expression compared to the RNA levels of *GAPDH*. Box plots summarize the qRT-PCR results. Each dot represents the result of a single measurement. Dot color identifies the cell clones. (**d**) Western blot

detection of epithelial and mesenchymal markers in protein lysates from A549 *JUNB* WT and mut cell clones as indicated. For the analyses of SNAIL1, SNAIL2, and ZEB1 nuclear extracts were utilized. Beta-actin and GSK-3 beta were used as the loading controls as indicated. Molecular weights are given in kDa. Representative results from one of three independent biological replicates are shown. Uncropped versions of the immunoblots including densitometry readings can be found in Figure S23. (**e**) Results from transwell migration assays performed with A549 *JUNB* WT and mut cell clones. Depicted are the percentages of the transwell areas covered by migrated cells. The height of the bars indicates mean values. Each dot represents the result of one out of three independent biological replicates. The color code identifies the cell clones. (**f**) Invasion assays performed with spheroids derived from A549 *JUNB* WT and mut cell clones embedded in a collagen I matrix. The height of the bars indicates for each of the cell clones the average number of sprouts per spheroid and mean sprout length from two fields of view each from three independent biological replicates. Error bars represent the standard error of the mean. The color code identifies the cell clones. Pictures of representative spheroids from one out of the three experiments are shown on the right. The scale bars represent 100 µm. (c, e, f) Stars indicate *p*-values corrected for multiple testing by the false discovery rate (FDR) method. *: FDR < 0.05, **: FDR < 0.01, ***: FDR < 0.001, ns: not significant; Mann-Whitney *U* test. (a-f) For all experiments, cells had been treated with TGFβ1 or a corresponding volume of solvent (CTRL) for 72 h prior to harvest.

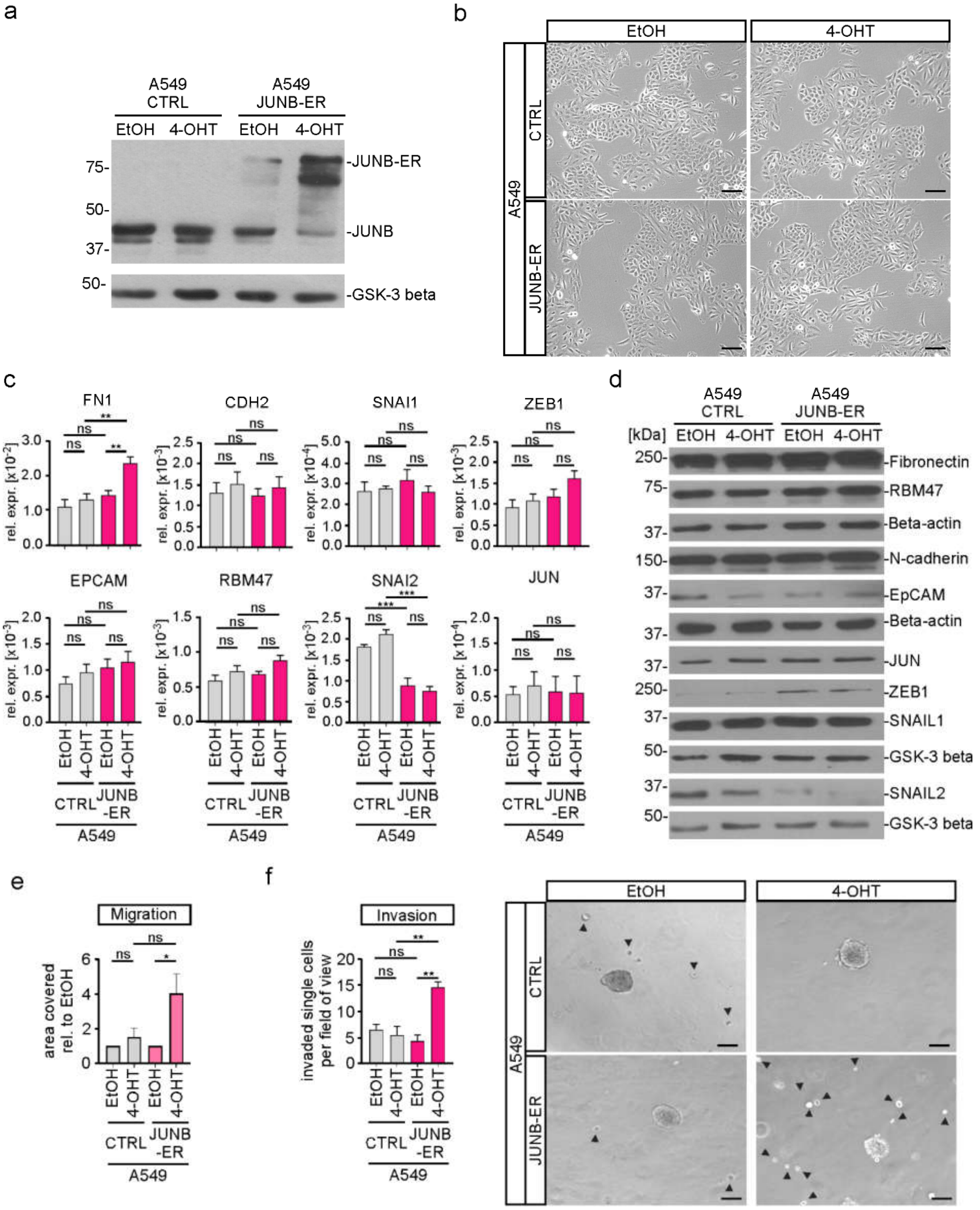

Figure S16.: Overexpression of JUNB does not elicit EMT in A549 cells. (**a**) Simultaneous western blot detection of endogenous JUNB und ectopic JUNB-ER in A549 cells that had been stably transduced with a retroviral vector for expression of a JUNB-ER fusion protein or a control construct (CTRL) harboring the JUNB coding region in antisense orientation. Nuclear extracts were probed with an antibody directed against JUNB. One representative result from three independent biological replicates is shown. Uncropped versions of immunoblots including densitometry readings can be found in Figure S23. (**b**) Representative phase-contrast microscopy pictures from one of three independent biological replicates showing the indicated A549 *JUNB-ER* or CTRL cells. The scale bars depict 200 µm. (**c**) Targeted gene expression analyses of epithelial and mesenchymal markers in A549 *JUNB-ER* or CTRL cells. RNA levels were measured by qRT-PCR and are given as relative expression compared to the RNA levels of *GAPDH*. (**d**) Western blot detection of epithelial and mesenchymal markers in protein lysates from A549 *JUNB-ER* or CTRL cells as indicated. For the

analyses of SNAIL1, SNAIL2, and ZEB1 nuclear extracts were utilized. Beta-actin and GSK-3 beta were used as the loading controls as indicated. Molecular weights are given in kDa. Representative results from one of three independent biological replicates are shown. Uncropped versions of the immunoblots including densitometry readings can be found in Figure S23. (**e**) Results from transwell migration assays performed with A549 *JUNB-ER* or CTRL cells. Depicted are the areas covered by cells on the lower surface of transwell inserts relative to the values of EtOH-treated cells. (**f**) Results from spheroid invasion assays performed with A549 *JUNB-ER* or CTRL cells embedded in a collagen I matrix. For quantification of the invasion assays shown on the left, single cells and small aggregates that had detached from the bulk of the cell aggregates were counted (exemplarily marked by arrow heads) in two fields of view per cell line and condition. Pictures of representative spheroids from one out of three independent biological replicates are shown on the right. The scale bars represent 100 μm. (c, e, f) The height of the bars represents the mean values from three independent biological replicates. Error bars depict the standard error of the mean. Stars indicate *p*-values corrected for multiple testing by the FDR method. *: FDR< 0.05, **: FDR < 0.01, ***: FDR < 0.001, ns: not significant; one-way ANOVA. (a-f) For all experiments, cells were treated with 100 nM 4-OHT or a corresponding volume of ethanol (EtOH) for 72 h prior to harvest.

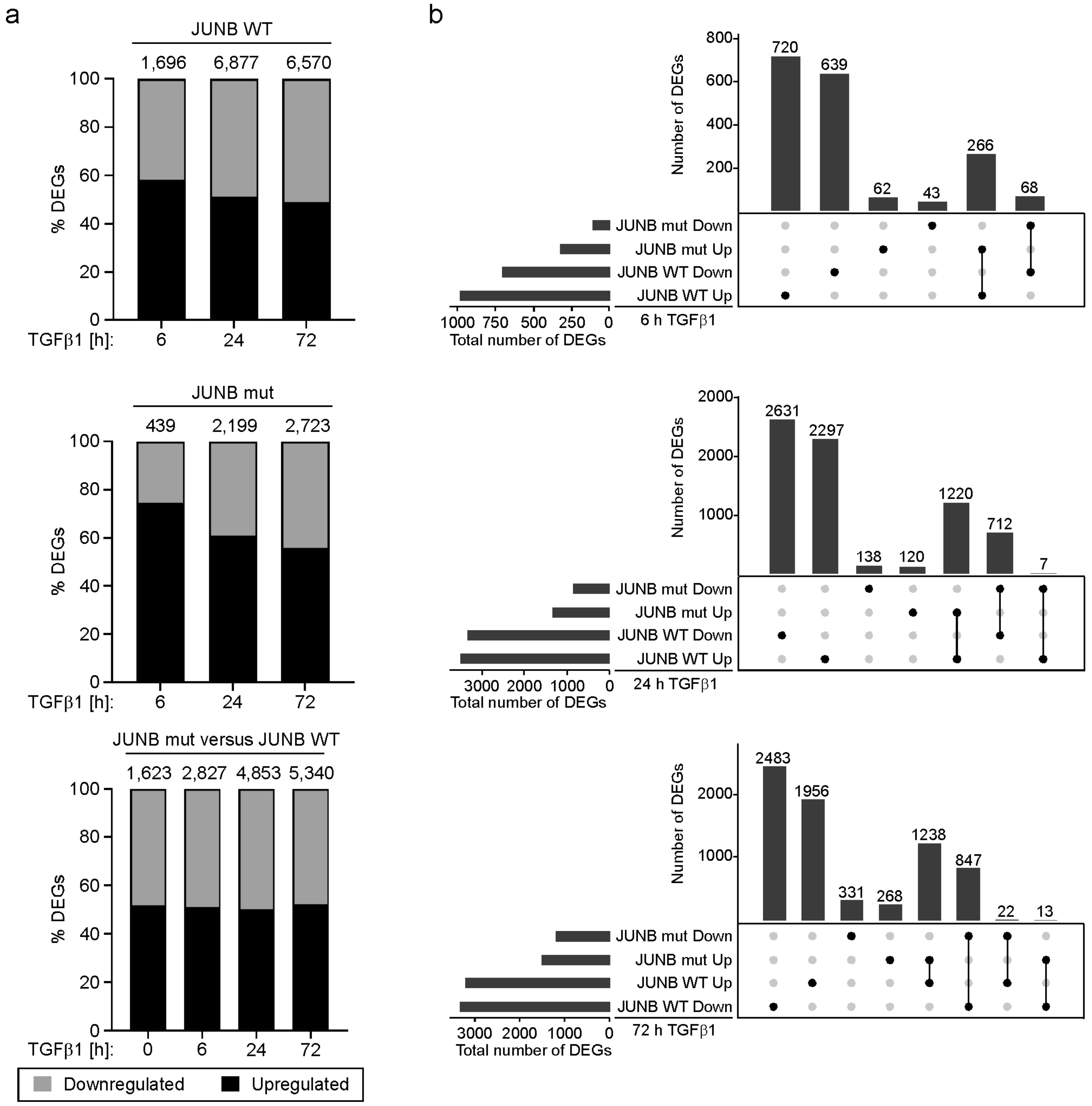

Figure S17.: JUNB is a major determinant of gene expression in treatment-naïve and TGFβ1-stimulated MCF10A cells (**a**) Percentages of differentially expressed genes (DEGs) up- and downregulated in MCF10A JUNB WT and mutant cells treated with TGFβ1 for the indicated time periods. The analyses were done for *JUNB* WT cell clones (top), *JUNB* mut cell clones (middle) and for the comparison of *JUNB* mut versus WT cell clones (bottom). (**b**) Upset plots depicting the numbers of DEGs that are up- and downregulated exclusively and commonly in MCF10A JUNB WT and JUNB mut cells at 6 h (top), 24 h (middle), and 72 h (bottom) of TGFβ1 stimulation. (a, b) The numbers on top of each bar indicate the total number of DEGs at each time point of TGFβ1 treatment compared to CTRL samples using an adj. p-value < 0.05 as threshold.

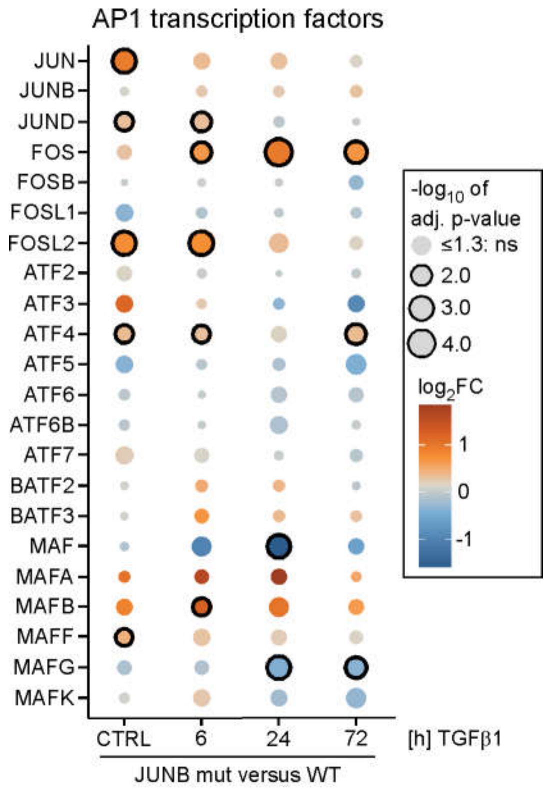

Figure S18.: *JUNB* inactivation alters the expression of some AP-1 family members. The dot plot shows the expression changes of members of the AP-1 TF family in MCF10A *JUNB* mut cell clones versus WT cell clones in untreated control cells (CTRL) and in response to treatment with TGFβ1 for the indicated time periods. Dot size indicates statistical significance as -log of the adj. p-value. Expression changes with a $-\log_{10}$ adj. *p*-value equal or below 1.3 are statistically not significant (ns). Dots that represent statistically significant gene expression changes are highlighted with black borders. Dot colors reflect the $\log_2$FC of the genes listed for each condition in *JUNB* mut versus WT cells.

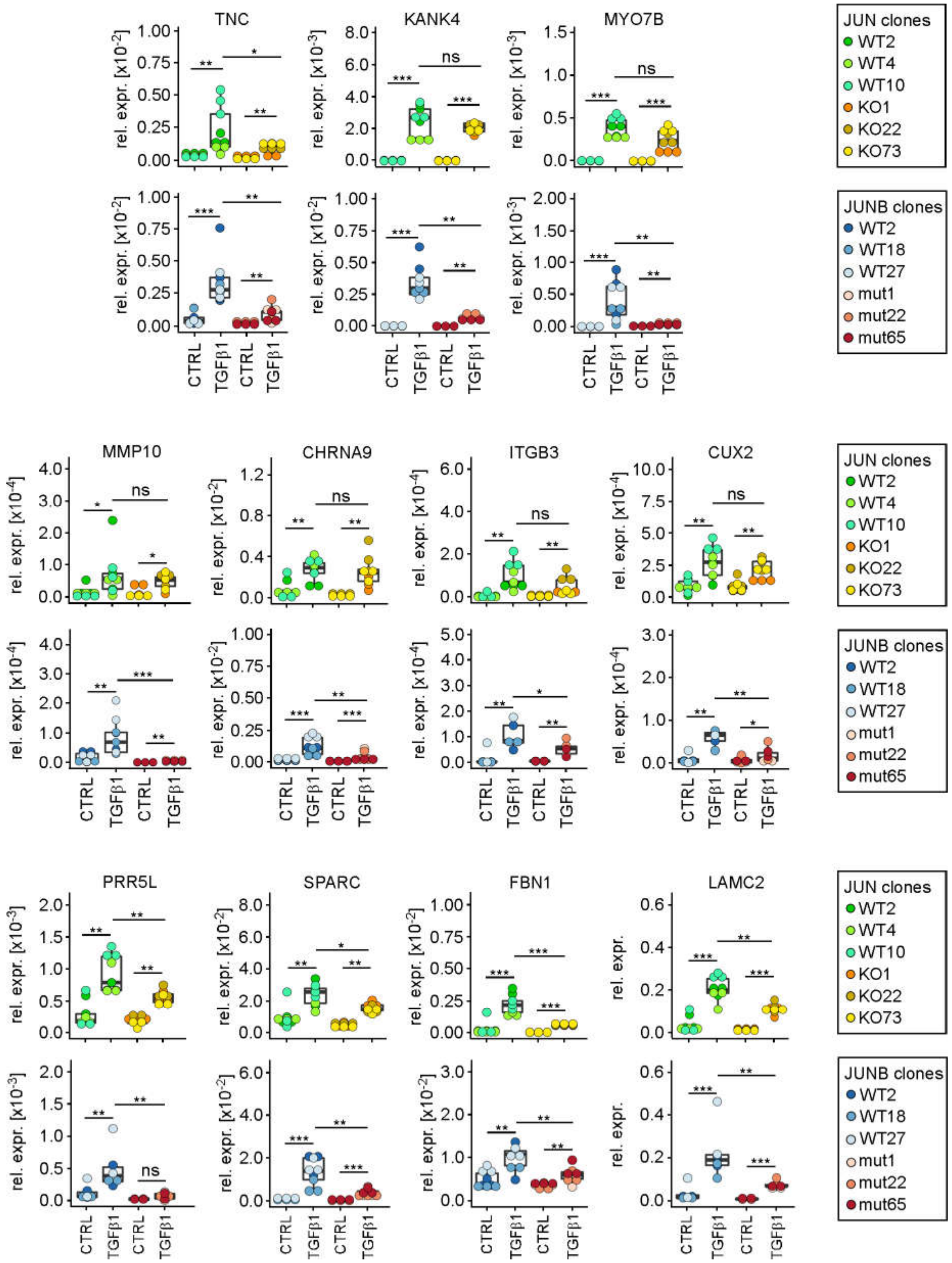

Figure S19.: Effects of *JUN* and *JUNB* inactivation on the expression of predicted *JUNB* target genes during TGFβ1-induced EMT. MCF10A *JUN* and *JUNB* WT and KO/mut cell clones were treated with TGFβ1 or solvent (CTRL) for 72 h and RNA levels of the genes indicated were measured by qRT-PCR. Transcript levels are expressed as relative expression compared to those of *GAPDH*. Box plots summarize qRT-PCR results. Each dot represents the result of a single measurement. Dot color identifies cell clones. Stars indicate *p*-values corrected for multiple testing by the FDR method. *: FDR < 0.05, **: FDR < 0.01, ***: FDR < 0.001, ns: not significant; Mann-Whitney *U* test.

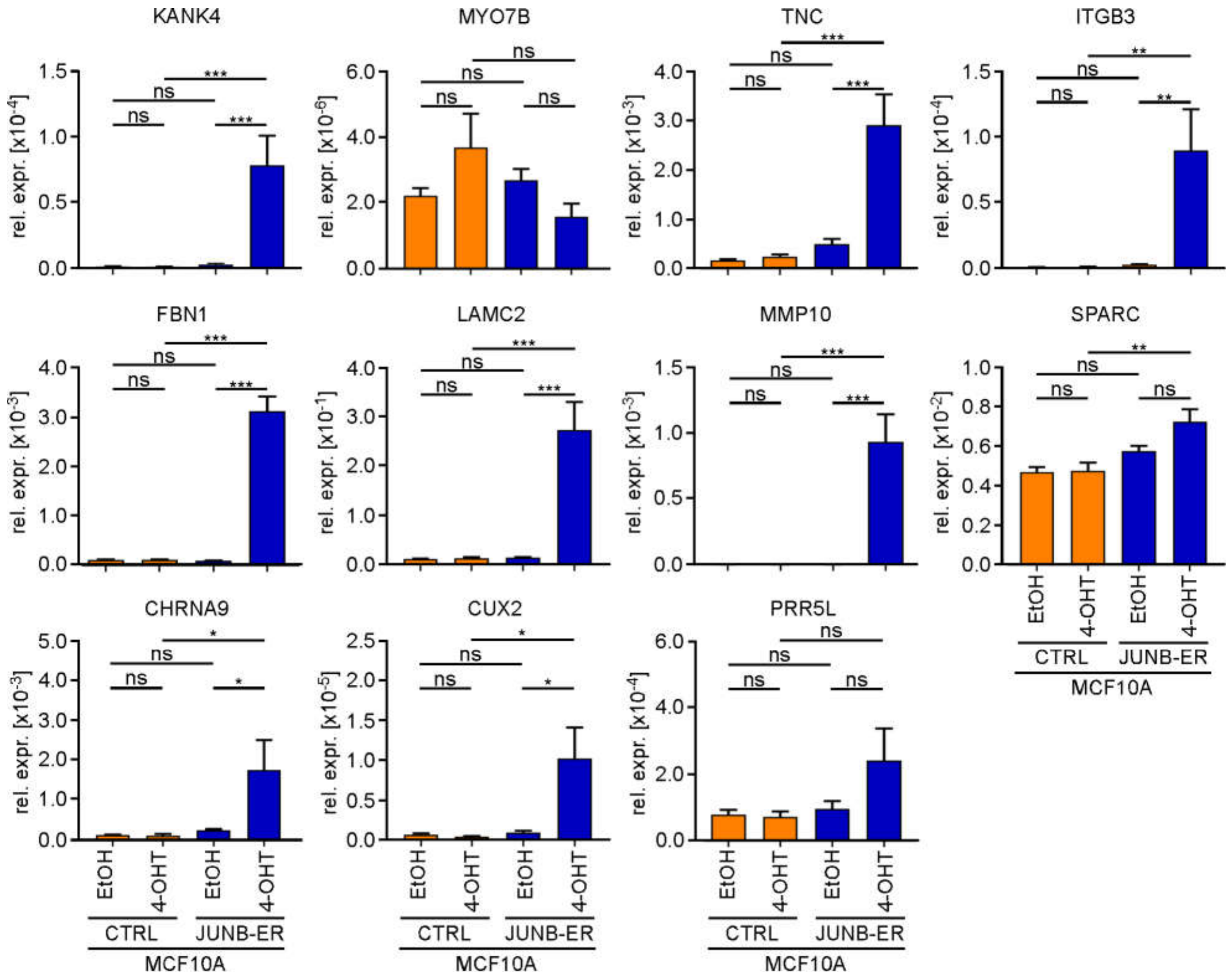

Figure S20.: Activation of TGFβ1-regulated and JUNB-dependent genes by JUNB-ER in MCF10A cells. MCF10A that had been stably transduced with a retroviral vector for expression of a JUNB-ER fusion protein or a control construct (CTRL) harboring the JUNB coding region in antisense orientation, were treated with 100 nM 4-OHT or a corresponding volume of ethanol (EtOH) for 72 h. Thereafter, RNA levels of the genes indicated were measured by qRT-PCR. Transcript levels are expressed as relative expression compared to those of *GAPDH*. The height of each bar represents the mean values from at least three independent biological replicates. Error bars depict the standard error of the mean. Stars indicate *p*-values corrected for multiple testing by the FDR method. *: FDR< 0.05, **: FDR < 0.01, ***: FDR < 0.001, ns: not significant; one-way ANOVA.

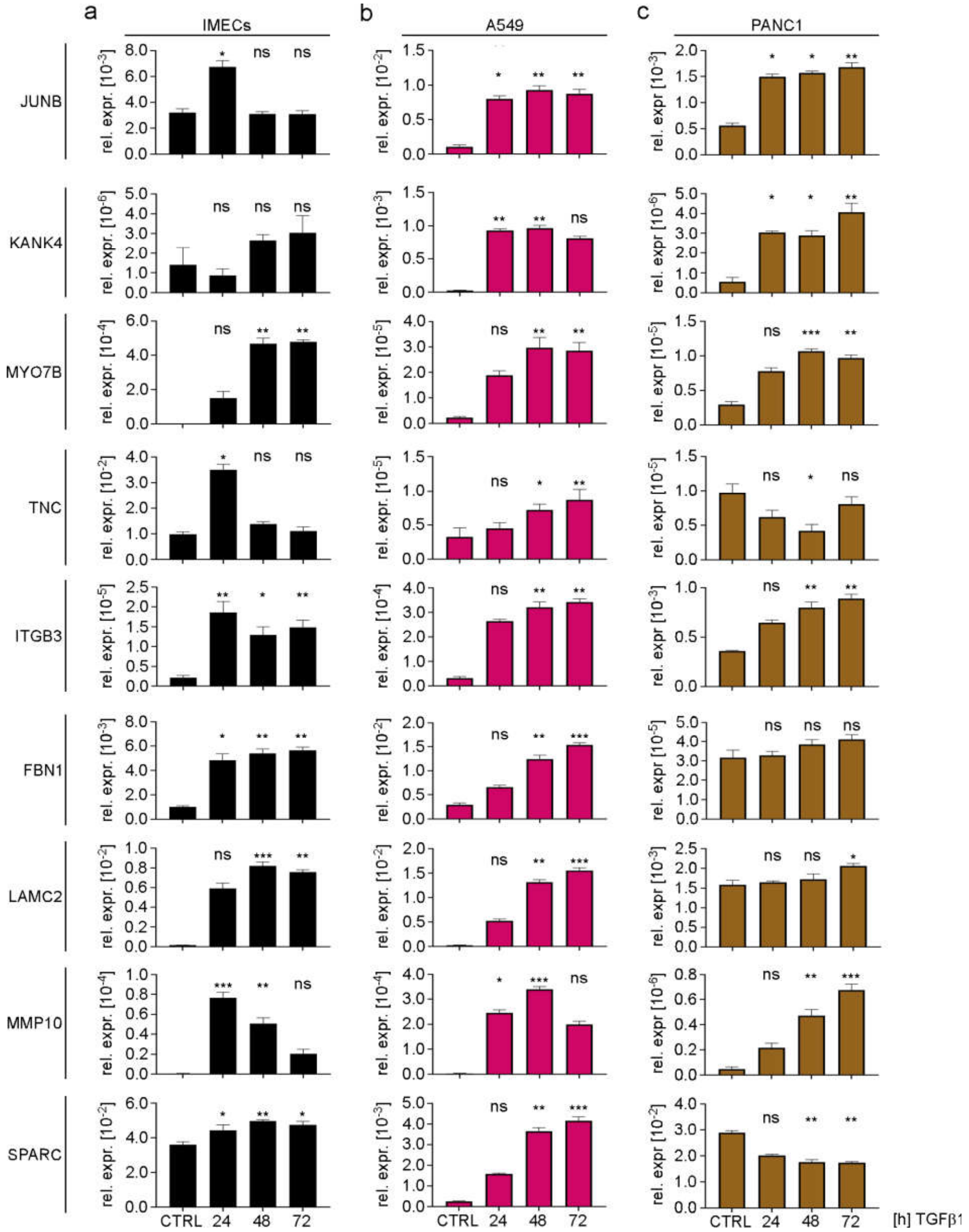

Figure S21.: Components of the TGFβ1-regulated EMT signature and *JUNB* can be activated by TGFβ1 in immortalized human mammary epithelial cells (IMECs) (**a**), the lung adenocarcinoma cell line A549 (**b**), and the pancreatic cancer cell line PANC1 (**c**). The expression of the indicated genes was analyzed by qRT-PCR in cells treated with solvent (CTRL) or TGFβ1 for the times indicated. Transcript levels are given as relative expression compared to those of *GAPDH*. The height of each bar represents mean values from three independent biological replicates. Error bars depict the standard error of the mean. Stars indicate *p*-values corrected for multiple testing by the FDR method. *: FDR< 0.05, **: FDR < 0.01, ***: FDR < 0.001, ns: not significant; Mann-Whitney *U* test.

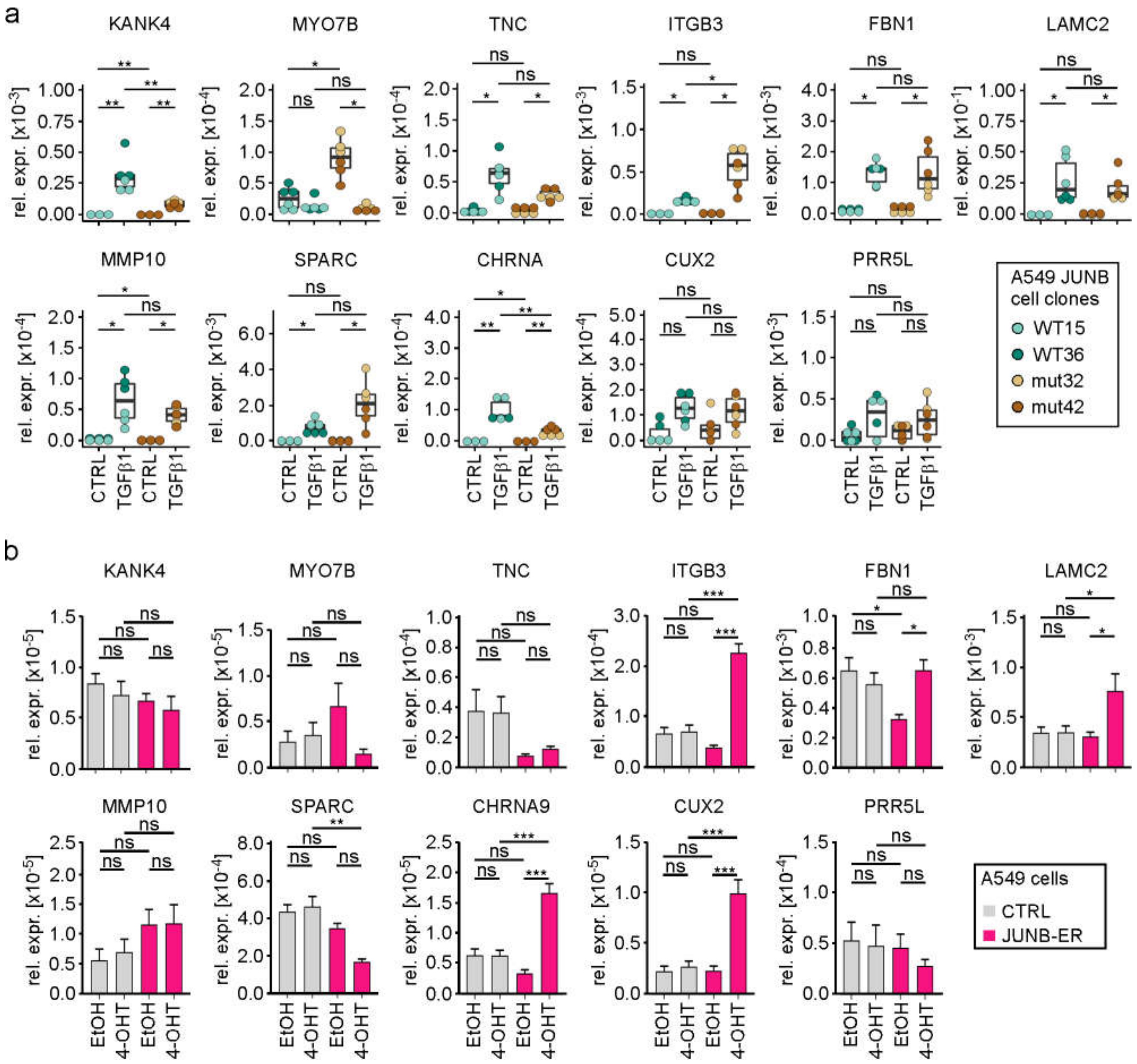

Figure S22.: JUNB-independent regulation of selected TGFβ1-inducible genes in A549 cells. (**a**) A549 *JUNB* WT and mut cell clones were treated with TGFβ1 or solvent (CTRL) for 72 h and RNA levels of the genes indicated were measured by qRT-PCR. Transcript levels are expressed as relative expression compared to those of *GAPDH*. Box plots summarize qRT-PCR results. Each dot represents the result of a single measurement. Dot color identifies cell clones. Stars indicate *p*-values corrected for multiple testing by the FDR method. *: FDR < 0.05, **: FDR < 0.01, ***: FDR < 0.001, ns: not significant; Mann-Whitney *U* test. (**b**) A549 cells that had been stably transduced with a retroviral vector for expression of a JUNB-ER fusion protein or a control construct (CTRL) harboring the JUNB coding region in antisense orientation, were treated with 100 nM 4-OHT or a corresponding volume of ethanol (EtOH) for 72 h. Thereafter, RNA levels of the genes indicated were measured by qRT-PCR. Transcript levels are expressed as relative expression compared to those of *GAPDH*. The height of each bar represents the mean values from at least three independent biological replicates. Error bars depict the standard error of the mean. Stars indicate *p*-values corrected for multiple testing by the FDR method. *: FDR< 0.05, **: FDR < 0.01, ***: FDR < 0.001, ns: not significant; one-way ANOVA.

(a)

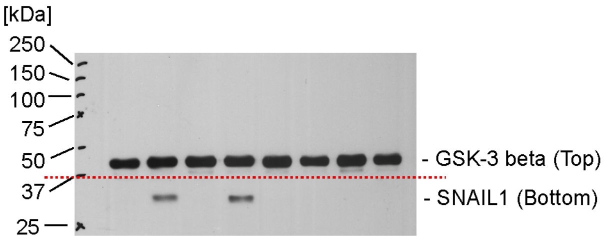

Epitope	Abundance relative to GSK-3 beta							
SNAIL1	0.06	0.45	0.06	0.54	0.04	0.04	0.03	0.03

(b)

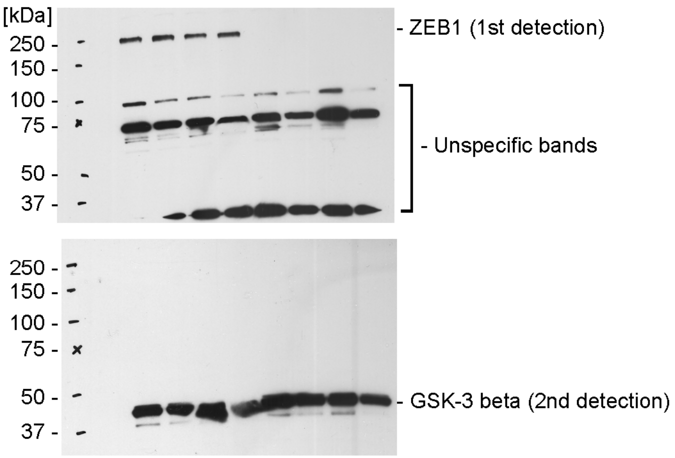

Epitope	Abundance relative to GSK-3 beta							
ZEB1	0.38	0.46	0.25	0.37	0.04	0.05	0.06	0.06

(c)

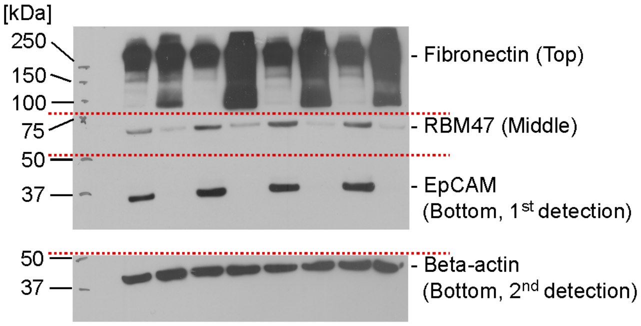

Epitope	Abundance relative to Beta-actin							
Fibronectin	1.47	1.68	1.02	2.52	1.44	2.57	1.19	2.38
RBM47	0.29	0.06	0.45	0.13	0.62	0.05	0.50	0.07
EpCAM	0.73	0.01	0.92	0.01	0.95	0.03	1.00	0.05

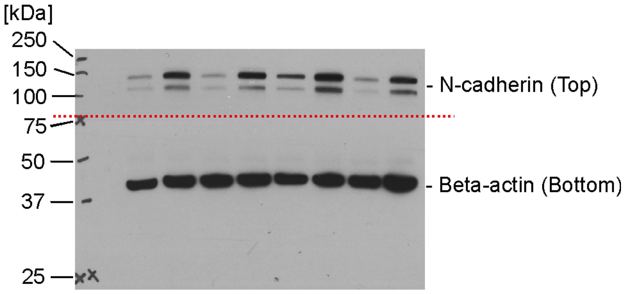

Epitope	Abundance relative to Beta-actin							
N-cadherin	0.10	0.47	0.07	0.37	0.21	0.68	0.09	0.37

(d)

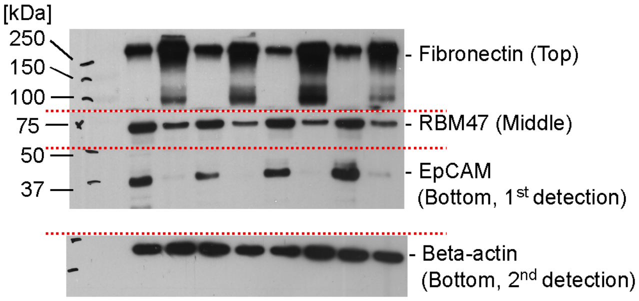

Epitope	Abundance relative to Beta-actin							
Fibronectin	0.64	1.40	0.52	1.88	0.55	1.69	0.64	1.65
RBM47	1.39	0.60	0.92	0.58	1.33	0.56	1.22	0.79
EpCAM	0.82	0.07	0.41	0.11	0.80	0.16	1.18	0.22

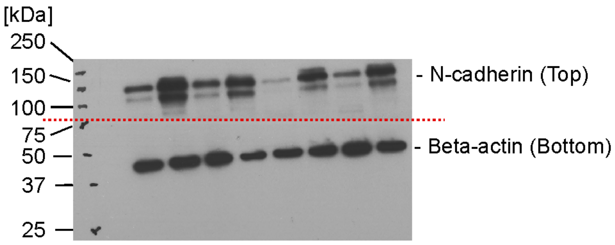

Epitope	Abundance relative to Beta-actin							
N-cadherin	0.42	0.92	0.24	0.91	0.25	0.83	0.52	1.13

continued →

(e)

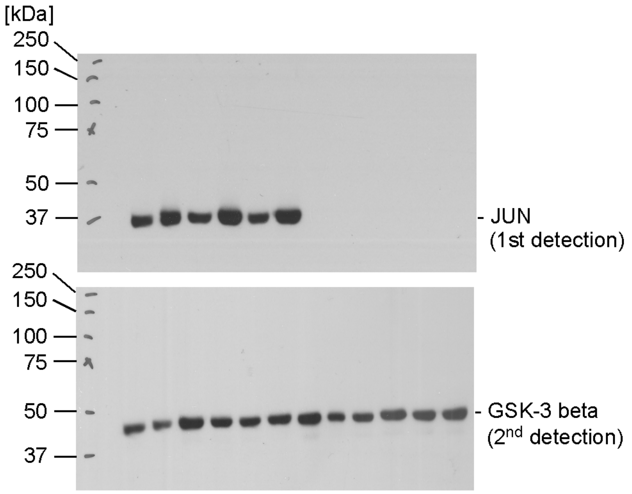

Epitope	Abundance relative to GSK-3 beta											
JUN	0.97	1.76	0.63	1.40	0.84	1.26	0.00	0.00	0.00	0.00	0.00	0.00

(f)

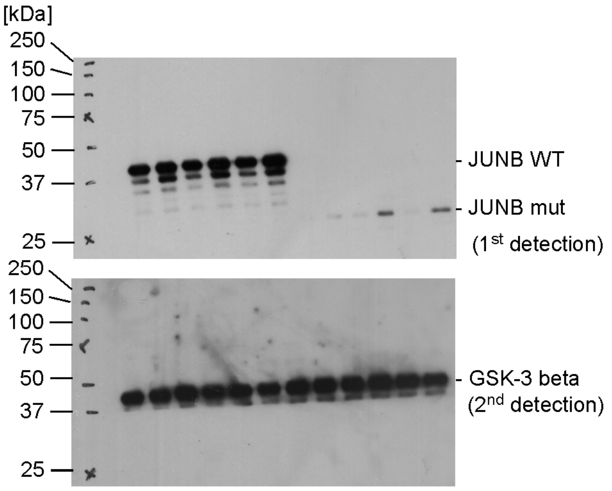

Epitope	Abundance relative to GSK-3 beta											
JUNB WT	0.99	1.10	0.74	0.98	0.80	1.18	0.10	0.09	0.07	0.07	0.08	0.07
JUNB mut	0.10	0.02	0.01	0.01	0.01	0.04	0.08	0.12	0.10	0.38	0.11	0.53

(g)

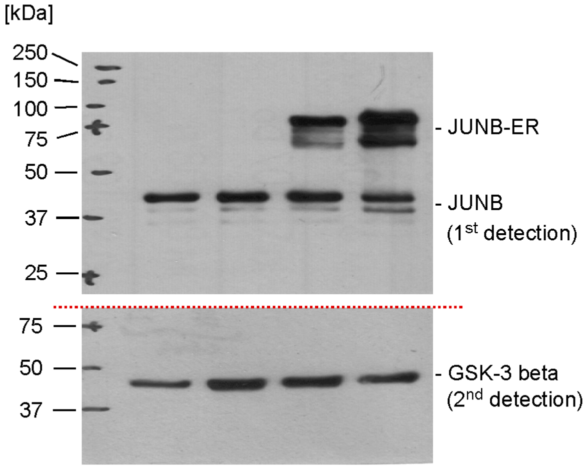

Epitope	Abundance relative to GSK-3 beta			
JUNB	1.2	0.83	0.97	1.32
JUNB-ER	0.04	0.03	1.22	2.58

continued →

(h)

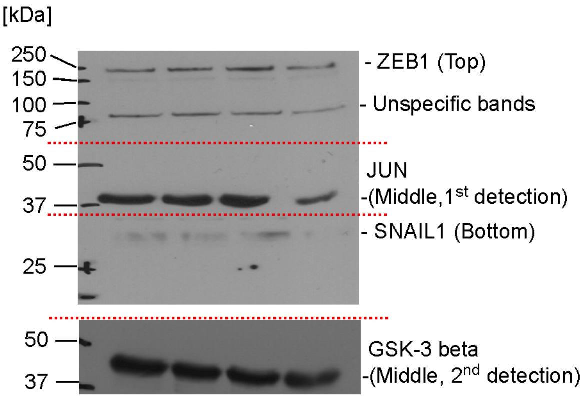

Epitope	Abundance relative to GSK-3 beta			
ZEB1	0.35	0.46	0.68	0.39
JUN	1.50	1.76	1.83	0.85
SNAIL1	0.38	0.39	0.57	0.25

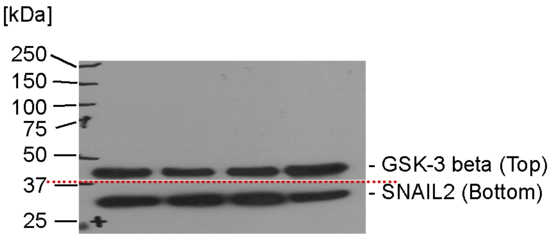

Epitope	Abundance relative to GSK-3 beta			
SNAIL2	0.98	1.28	1.18	0.95

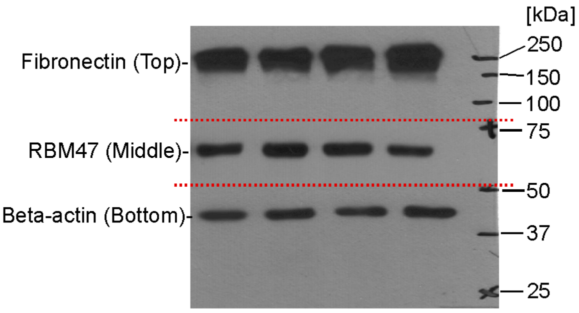

Epitope	Abundance relative to GSK-3 beta			
Fibronectin	1.14	1.10	1.12	1.20
RBM47	1.05	1.11	1.22	0.92

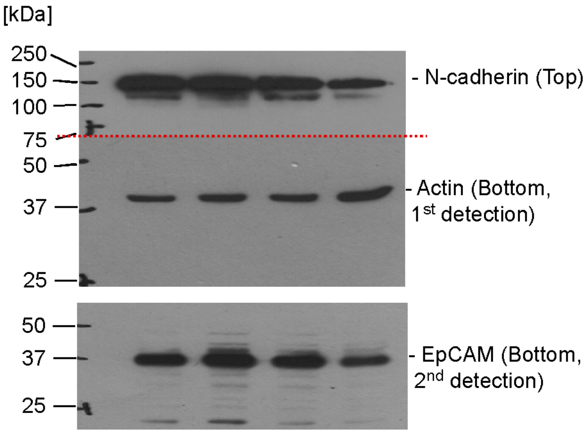

Epitope	Abundance relative to GSK-3 beta			
N-cadherin	3.57	2.94	2.76	1.21
EpCAM	2.36	2.40	2.39	1.39

(i)

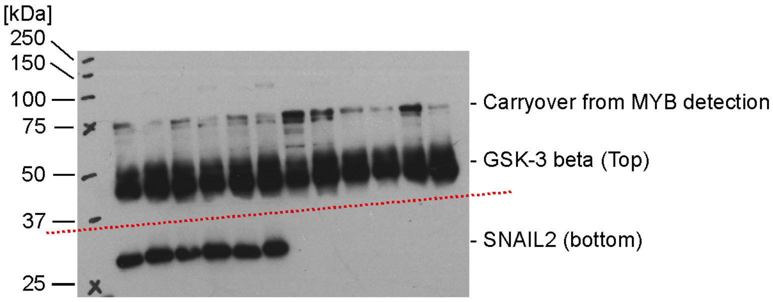

Epitope	Abundance relative to GSK-3 beta											
SNAIL2	0.63	0.76	0.56	0.75	0.62	0.63	0.07	0.02	0.02	0.00	0.00	0.00

continued →

(j)

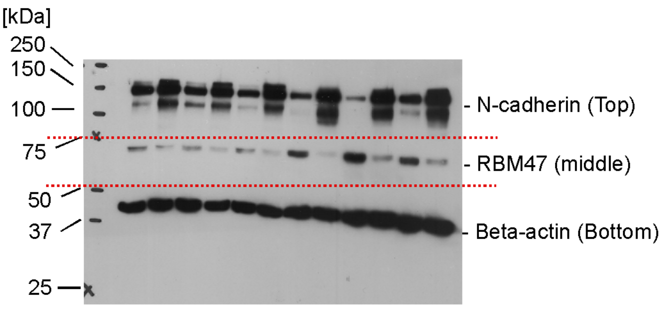

Epitope	Abundance relative to Beta-actin											
Fibronectin	0.72	1.00	0.56	1.05	0.47	1.09	0.41	1.18	0.28	0.90	0.50	1.02
EpCAM	0.24	0.09	0.14	0.10	0.20	0.13	0.47	0.20	0.72	0.30	0.44	0.25

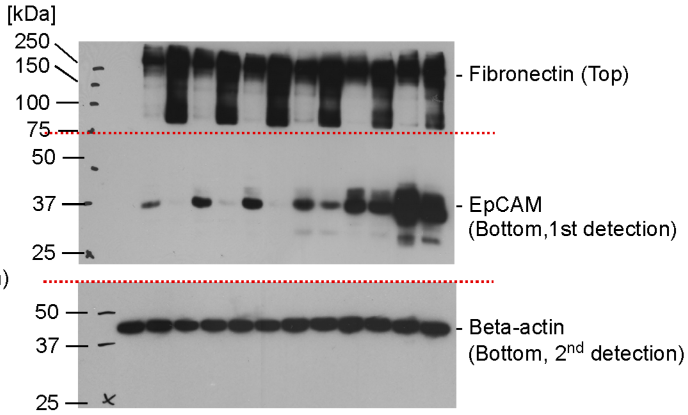

Epitope	Abundance relative to Beta-actin											
Fibronectin	0.72	1.66	0.91	1.84	0.81	1.93	0.90	1.72	0.72	1.40	0.79	1.35
EpCAM	0.14	0.00	0.38	0.00	0.43	0.03	0.51	0.34	0.80	0.89	1.50	1.28

(k)

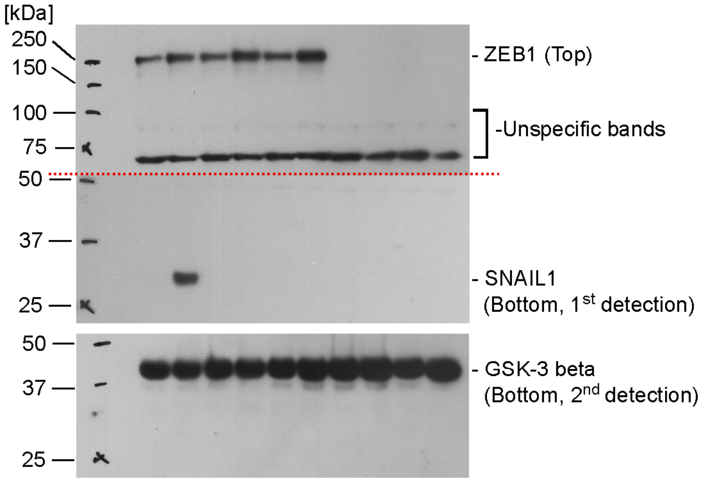

Epitope	Abundance relative to GSK-3 beta									
ZEB1	0.38	0.49	0.39	0.62	0.46	0.60	0.10	0.09	0.10	0.07
SNAIL1	0.02	0.49	0.00	0.00	0.00	0.00	0.00	0.00	0.00	0.00

(l)

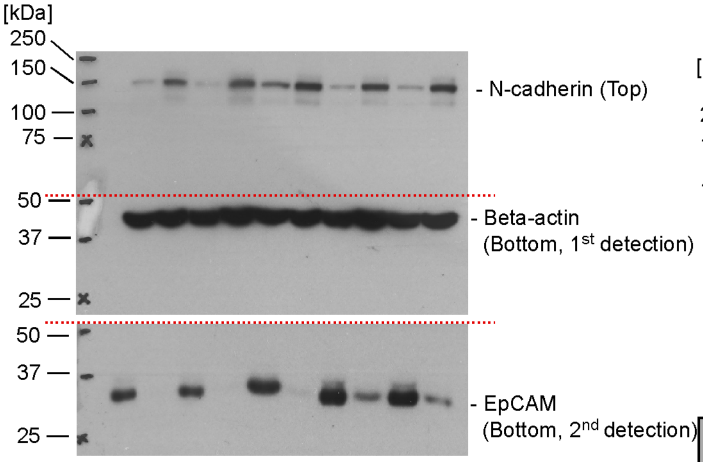

Epitope	Abundance relative to Beta-actin									
N-cadherin	0.33	0.87	0.48	1.02	0.81	1.06	0.50	0.90	0.58	1.24
EpCAM	0.65	0.06	0.59	0.10	0.85	0.18	0.92	0.47	1.02	0.38

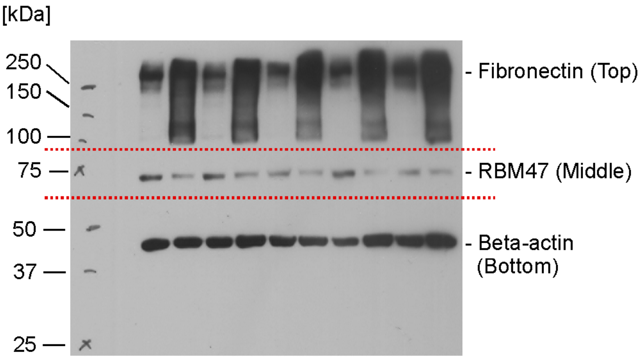

Epitope	Abundance relative to Beta-actin									
Fibronectin	0.73	2.13	0.82	1.79	0.86	2.51	1.48	1.72	0.96	1.51
RBM47	0.34	0.16	0.32	0.20	0.32	0.28	0.71	0.17	0.25	0.19

continued →

(m)

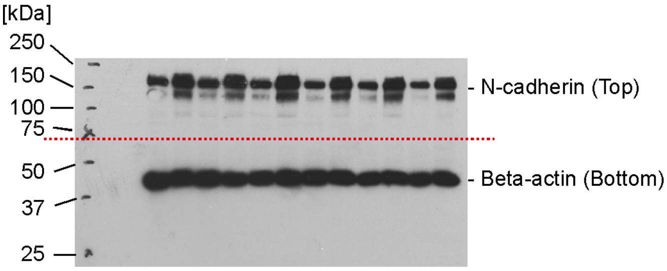

Epitope	Abundance relative to Beta-actin											
N-cadherin	0.50	1.04	0.70	1.30	0.69	1.29	0.47	1.14	0.57	1.20	0.40	1.08

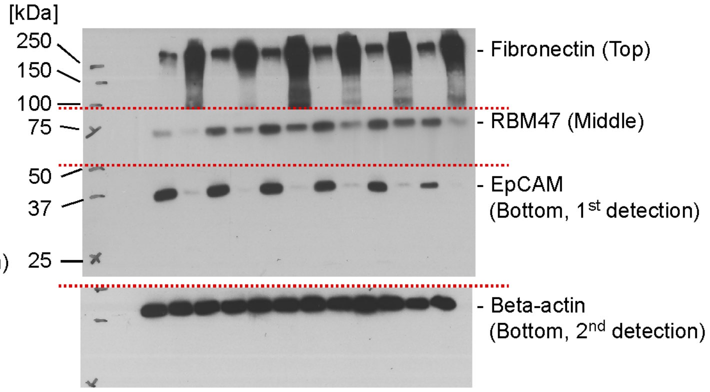

Epitope	Abundance relative to Beta-actin											
Fibronectin	0.40	1.30	0.46	1.01	0.44	1.61	0.46	1.20	0.37	1.26	0.56	1.71
RBM47	0.19	0.11	0.53	0.36	0.67	0.53	0.73	0.45	0.61	0.54	0.79	0.47
EpCAM	0.79	0.10	0.72	0.07	0.65	0.07	0.54	0.08	0.44	0.07	0.35	0.05

(n)

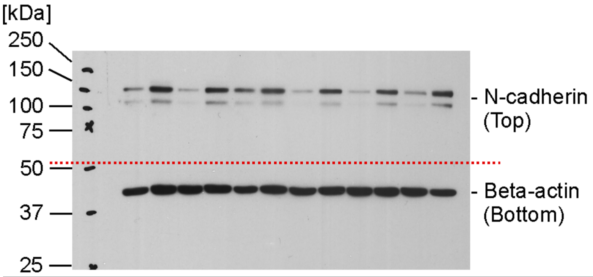

Epitope	Abundance relative to Beta-actin											
N-cadherin	0.33	0.71	0.14	0.66	0.43	0.59	0.13	0.54	0.14	0.55	0.31	0.91

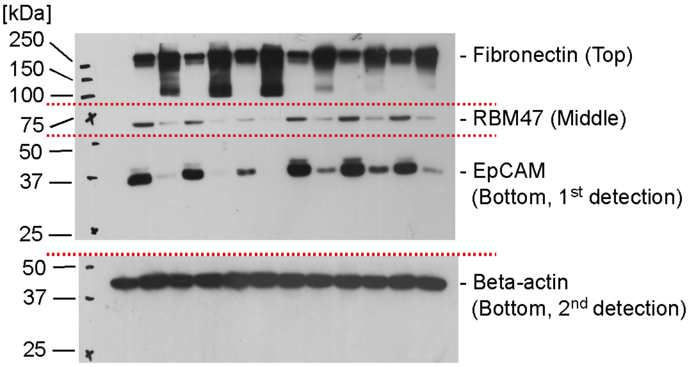

Epitope	Abundance relative to Beta-actin											
Fibronectin	0.75	1.36	0.49	1.76	0.76	1.98	0.72	1.49	0.88	1.24	0.86	1.27
RBM47	0.55	0.10	0.29	0.08	0.13	0.01	0.47	0.27	0.66	0.30	0.58	0.31
EpCAM	0.59	0.16	0.33	0.14	0.18	0.09	0.51	0.33	0.69	0.36	0.62	0.36

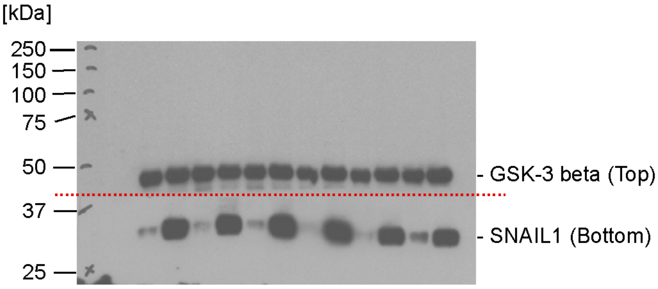

Epitope	Abundance relative to GSK-3 beta											
SNAIL1	0.15	0.77	0.18	0.84	0.20	0.94	0.28	0.94	0.31	0.76	0.27	0.61

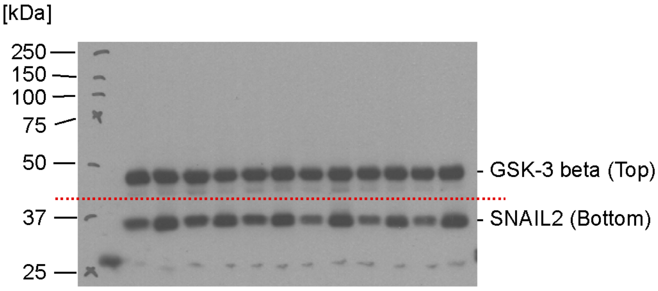

Epitope	Abundance relative to GSK-3 beta											
SNAIL2	0.73	0.94	0.72	0.93	0.72	0.85	0.58	0.92	0.55	0.77	0.58	0.96

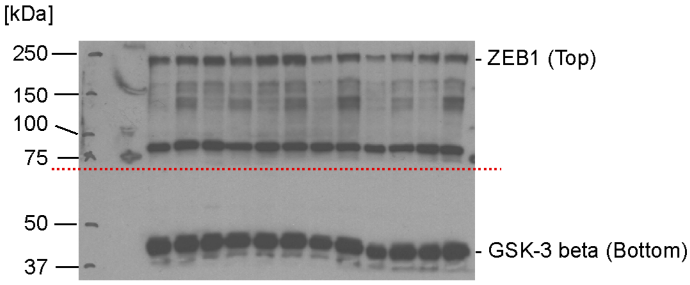

Epitope	Abundance relative to GSK-3 beta											
ZEB1	0.65	0.67	0.74	0.73	0.84	0.88	0.58	0.64	0.51	0.58	0.71	0.72

continued →

(o)

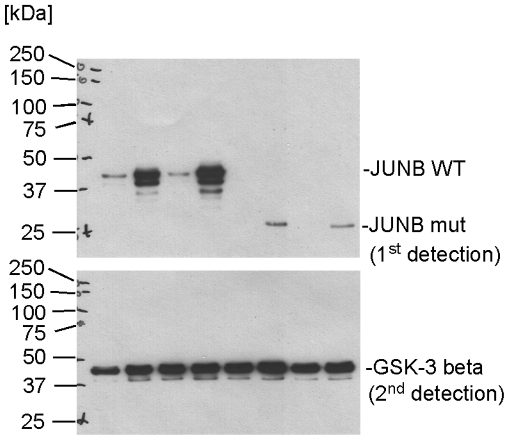

Epitope	Abundance relative to Beta-actin							
JUNB WT	0.28	0.99	0.18	1.14	0.06	0.07	0.03	0.00
JUNB mut	0.01	0.00	0.04	0.07	0.07	0.35	0.02	0.27

(p)

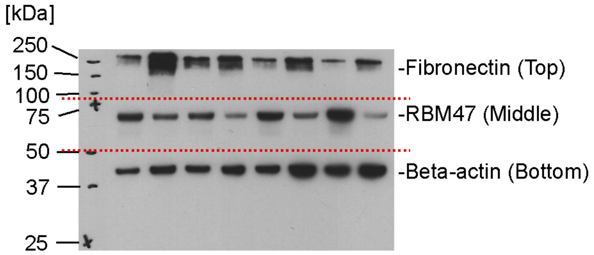

Epitope	Abundance relative to Beta-actin							
Fibronectin	0.60	1.59	0.81	0.87	0.38	0.59	0.18	0.36
RBM47	1.26	0.51	0.76	0.33	0.95	0.35	1.05	0.18

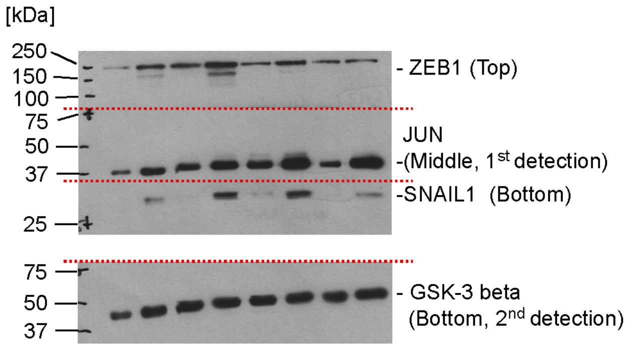

Epitope	Abundance relative to GSK-3 beta							
ZEB1	0.33	1.12	0.99	1.93	0.70	1.00	0.67	0.50
JUN	0.78	1.02	0.95	1.42	1.39	1.89	1.01	1.77
SNAIL1	0.00	0.38	0.03	1.02	0.25	1.09	0.07	0.39

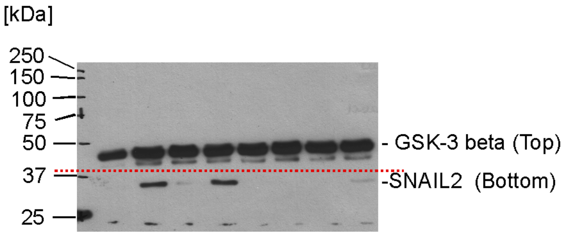

Epitope	Abundance relative to GSK-3 beta							
SNAIL2	0.04	0.57	0.03	0.56	0.01	0.01	0.00	0.06

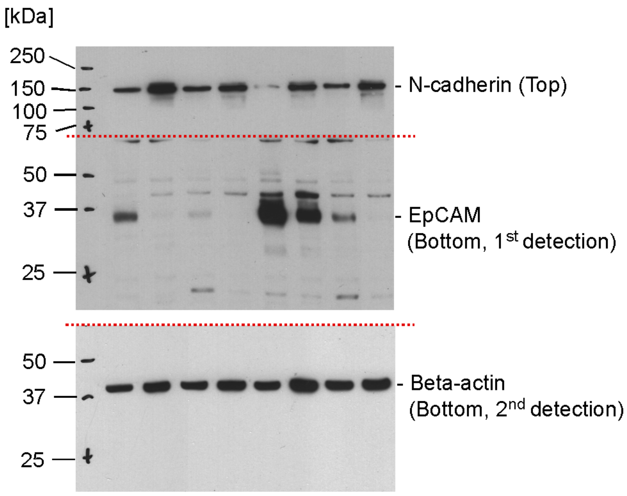

Epitope	Abundance relative to Beta-actin							
N-cadherin	0.65	1.24	0.77	0.84	0.15	0.67	0.53	0.86
EpCAM	0.87	0.04	0.14	0.07	2.04	1.04	0.52	0.04

continued →

(q)

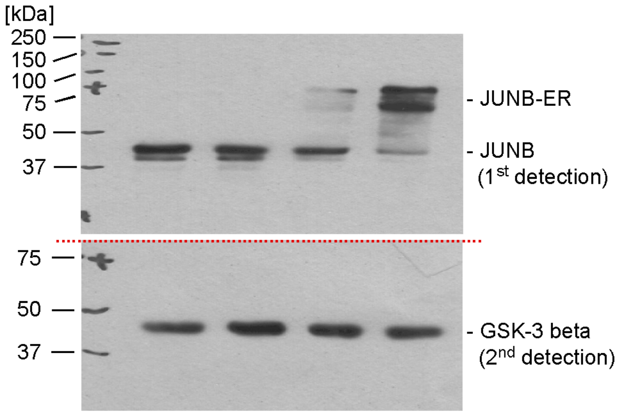

Epitope	Abundance relative to GSK-3 beta			
JUNB	1.54	1.02	0.84	0.46
JUNB-ER	0.00	0.00	0.32	2.02

(r)

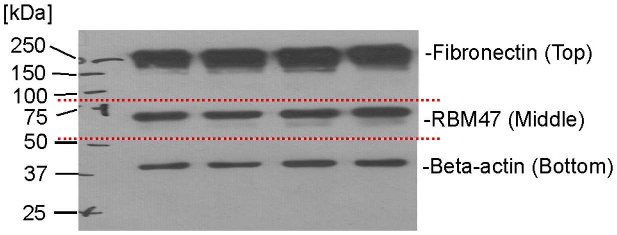

Epitope	Abundance relative to Beta-actin			
Fibronectin	1.45	1.73	1.49	1.41
RBM47	1.21	1.15	1.12	1.29

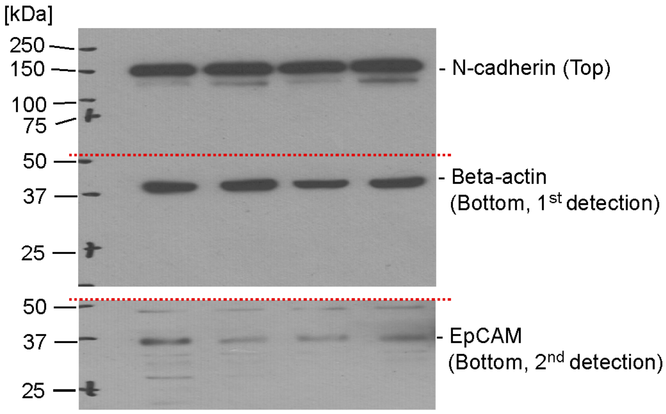

Epitope	Abundance relative to Beta-actin			
N-cadherin	0.82	0.90	1.15	1.23
EpCAM	0.46	0.22	0.33	0.64

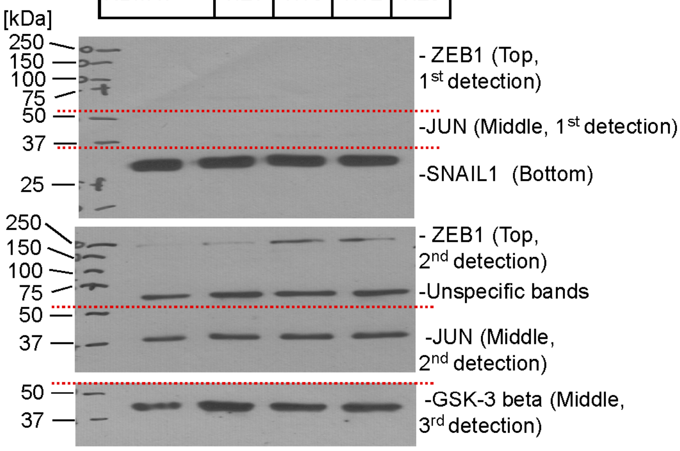

Epitope	Abundance relative to GSK-3 beta			
ZEB1	0.05	0.05	0.39	0.27
JUN	0.84	0.71	0.94	0.91
SNAIL1	1.52	0.94	1.17	1.04

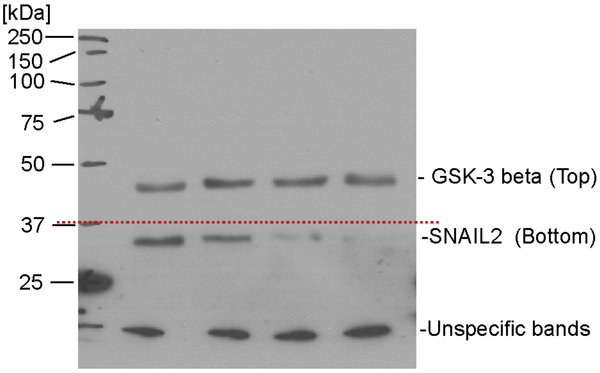

Epitope	Abundance relative to GSK-3 beta			
SNAIL2	1.33	0.88	0.16	0.17

Figure S23.: Compilation of uncropped immunoblots for all figures and quantification of proteins analyzed. The positions of molecular weight standards [kDa] are given on the left of the blots, while the proteins detected are identified on the right. In cases where membranes were divided into two or more pieces to enable simultaneous detection of different epitopes, dotted red lines indicate the position(s) of the cut(s). It is also indicated when a membrane was incubated multiple times to sequentially detect different proteins. Carryover signals from previous detections are marked, too. Tables show the relative protein abundances which were determined by dividing background-corrected densitometric readings for the signals of the proteins-of-interest by the corresponding values

for the loading controls. The uncropped immunoblots images are related to: (**a**) Figure 4c, (**b**) Figure 4d, (**c**) Figure 4g, (**d**) Figure 4j, (**e**) Figure 5c, (**f**) Figure 5d, (**g**) Figure 6b, (**h**) Figure 6e, (**i**) Figure S6b, (**j**) Figure S6g, (**k**) Figure S7b, (**l**) Figure S7e, (**m**) Figure S13b, (**n**) Figure S13d, (**o**) Figure S15a, (**p**) Figure S15d, (**q**) Figure S16a, (**r**) Figure S16d.

Table S1.: List of cell lines and their culture conditions

<table>
<tr><th>Cell line</th><th>Source</th><th>Culture conditions</th></tr>
<tr><td>MCF10A[a]</td><td>ATCC American Type Culture Collection (CRL-10317, Manassas, VA, USA)</td><td rowspan="2">Gibco™ Advanced DMEM/F12 with 5 % (v/v) horse serum, 1% (v/v) penicillin/streptomycin, 20 ng/ml human EGF, 0.5 µg/ml hydrocortisone, 0.1 µg/ml cholera toxin, and 10 µg/ml insulin at 37 °C and 5 % CO_2</td></tr>
<tr><td>MCF10A WT and KO/mut clonal derivatives; JUNB-ER and CTRL derivatives[a]</td><td>Generated in this study</td></tr>
<tr><td>IMEC[b]</td><td>Gift from Dr. Alessio Zippo (University of Trento, Trento, Italy)</td><td>Supplemented MCF10A medium with 17.5 µg/ml bovine pituitary extract at 37 °C and 5 % CO_2.</td></tr>
<tr><td>PANC1[a]</td><td>Gift from Dr. Thomas Brabletz (Friedrich-Alexander-Universität, Erlangen-Nürnberg, Germany)</td><td rowspan="3">Dulbecco’s modified Eagle’s medium (DMEM) supplemented with 10% (v/v) foetal calf serum, 1% penicillin/streptomycin, 0.01 M HEPES/KOH and 1X MEM non-essential amino acids at 37 °C and 5 % CO_2.</td></tr>
<tr><td>A549[a]</td><td>CLS Cell Lines Service GmbH (#300114, Eppelheim, Germany)</td></tr>
<tr><td>A549 WT and mut clonal derivatives; JUNB-ER and CTRL derivatives[a]</td><td>Generated in this study</td></tr>
</table>

[a] Cell line identity was determined by SNP profiling at Multiplexion Inc. (Friedrichshafen, Germany)
[b] STR profile of IMEC cells was generated at Multiplexion Inc.

Table S2.: Sequences of oligonucleotides and their application

Gene	Application	Forward primer (5´ - 3´)	Reverse primer (5´ - 3´)
FN1	qRT-PCR	AAAAGTGCCAGATGATGAGC	TGGCACCGAGATATTCCTTC
CDH2	qRT-PCR	GCCAGGCCAAACAACTTTTA	TGGATGGGTCTTTCATCCAT
RBM47	qRT-PCR	GAAATGTATGAACGCGGGCC	CCCCAGGTGATGAGTCCAAC
EPCAM	qRT-PCR	TGCAGGGTCTAAAAGCTGGT	GTCCCACGCACACACATTTG
MMP10	qRT-PCR	CTTGTGCTGTTGTGTCTGCC	AGGAGCTGAAGTGACCAACG
TNC	qRT-PCR	GGAAGAGACGGTCAACCTGG	GCCTTCAGGTGCAGGTAAGT
KANK4	qRT-PCR	GTCAATCTGCAGGACCACGA	CGAGGGAGGAGTCCAGAGAA
MYO7B	qRT-PCR	ACGTCATCCTCATCGCCATC	GTTCATGGCACTCAGGAGCT
LAMC2	qRT-PCR	GGAGCTGGAGTTTGACACGA	TGAGAGGCTGGTCCATCAGA
SPARC	qRT-PCR	AGCTGCGGGTGAAGAAGATC	GAAAAAGCGGGTGGTGCAAT
FBN1	qRT-PCR	GGGGAGCTACAAGTGCATGT	AGCCTCTGGGGAGAGTGAAT
ITGB3	qRT-PCR	GCCCTGCTCATCTGGAAACT	TTGGTGAAGGTAGACGTGGC
CUX2	qRT-PCR	GGAGTGGGAGTTCTGAAGGC	AGTTCCTCCCTCCCTTACCC
PRR5L	qRT-PCR	TCCAGTGAGCCCAACATCAC	GGTCCTCATCCTGGTTGCAA
CHRNA9	qRT-PCR	CCGAGATCAGTACGATGGCC	GATCAGCCCATCATACCGCA
JUN	qRT-PCR	ACATGCTCAGGGAACAGGTG	TCTCTCCGTCGCAACTTGTC
JUNB	qRT-PCR	ACACCAACCTCAGCAGCTAC	GTCTGCGGTTCCTCCTTGAA
GAPDH	qRT-PCR	CCCACTCCTCCACCTTTGAC	GAGATTCAGTGTGGTGGGGG
*TNC*_C	ChIP-qPCR	TGGCAGGGATGGAAAGGAAC	CGATGTCCCCTTTTGGAGCT
TNC_1	ChIP-qPCR	AGCGAGCTCCTTCCTCTGTA	CCAGGAGTGAGTGCGTCTTT
*KANK4*_C	ChIP-qPCR	GTTTGGTGCCACTACCTCGA	GCCTGCAGACTTCTGAGGTT
*KANK4*_1	ChIP-qPCR	ACACAAAACCAGAAGCCCCA	GTGGAGATGCCCAAACCTGA
*KANK4*_2	ChIP-qPCR	CAGCACACCCCTTCATCTGT	CAAATGGCGATGCTCTGAGC
*MYO7B*_C	ChIP-qPCR	CAAGTGGAGGACTGGAGCTG	GCGCTGGCATCCATTAAAGG
*MYO7B*_1	ChIP-qPCR	GATCCGAGTGTGCCAAGGAA	CCCCAGTCCTGCTTTCTGAG
*MYO7B*_2	ChIP-qPCR	GTCTGGCTGACTCAGTGTCC	GACAGGTCACCAAAGAGGGG
*MYO7B*_3	ChIP-qPCR	GCCCTGTTCCACATCTGTCA	CCAGCTAAAGGGTGAGCTCC

Table S3.: List of antibodies and their applications

Primary antibodies					
**Antigen**	**Species of origin**	**Working dilution**			**Supplier (catalogue no.)**
		**WB[a]**	**IF[b]**	**ChIP[c]**	
SNAIL1	Rabbit	1:1000	-	-	Cell Signaling Technology (C15D3)
SNAIL2	Rabbit	1:1000	-	-	Cell Signaling Technology (C19G7)
ZEB1	Rabbit	1:5000	-	-	Sigma-Aldrich (#HPA027524)
JUN	Rabbit	1:1000	-	-	Cell Signaling Technology (60A8)
JUNB	Rabbit	1:10000	-	1:50	Cell Signaling Technology (C37F9)
EpCAM	Rabbit	1:1000	-	-	Cell Signaling Technology (E6V8Y)
EpCAM	Mouse	-	1:400	-	Cell Signaling Technology (VU1D9)
N-cadherin	Rabbit	1:1000	-	-	Cell Signaling Technology (D4R1H)
Fibronectin	Rabbit	1:2000		-	Abcam (#ab2413)
RBM47	Rabbit	1:1000	-	-	Abcam (#ab167164)
Beta-actin	Mouse	1:10000		-	MP Biomedicals (0869100-CF)
GSK-3 beta	Rabbit	1:1000		-	BD Biosciences (#610404)

Secondary antibodies					
**Antigen**	**Species of origin**	**Conjugation**	**Working dilution**		**Supplier (catalogue no.)**
			**WB[a]**	**IF[b]**	
Mouse IgG	Goat	Horseradish peroxidase (HRP)	1:10000	-	Dianova (Biozol) (#115-035-146)
Rabbit IgG	Goat	Horseradish peroxidase (HRP)	1:10000	-	Dianova (Biozol) (#111-035-045)

Mouse IgG	Donkey	Alexa Fluor555	-	1:500	Invitrogen (Thermo Fisher Scientific) (#A-31570)
Rabbit IgG	Goat	Alexa Fluor488	-	1:200	Invitrogen (Thermo Fisher Scientific) (#A-11008)

[a]WB: Western blotting
[b]IF: Immunofluorescence
[c]ChIP: Chromatin immunoprecipitation

Table S4.: List summarizing the genomic status of MCF10A and A549 cell clones derived from CRISPR/Cas9 genome-editing experiments

MCFD10A cells				
**Clone name**	**sgRNA target(s)**	**Allele 1**	**Allele 2**	**Reference for genomic coordinates**
SNAI1 WT6	exons 1 and 2	WT	WT	5′-end of ENSE00001172559
SNAI1 WT7	exons 1 and 2	WT	WT	5′-end of ENSE00001172559
SNAI1 KO112	exons 1 and 2	1665 bp deletion starting at position 126	1665 bp deletion starting at position 126	5′-end of ENSE00001172559
SNAI1 KO177	exons 1 and 2	inverted sequence (positions 125-1791)	inverted sequence (positions 125-1791)	5′-end of ENSE00001172559
ZEB1 WT1	exon 6	WT	WT	5′-end of ENSE00003701577
ZEB1 WT5	exon 6	WT	WT	5′-end of ENSE00003701577
ZEB1 KO8	exon 6	inverted sequence (positions 15-439)	inverted sequence (positions 15-439)	5′-end of ENSE00003701577
ZEB1 KO20	exon 6	414 bp deletion starting at position 15	414 bp deletion starting at position 15	5′-end of ENSE00003701577
SNAI1 KO-ZEB1 WT3	*SNAI1*: exons 1 and 2 *ZEB1*: exon 6	*SNAI1*: 1665 bp deletion starting at position 126 *ZEB1*: WT	*SNAI1*: 1665 bp deletion starting at position 126 *ZEB1*: WT	*SNAI1*: 5′-end of ENSE00001172559 *ZEB1*: 5′-end of ENSE00003701577
SNAI1 KO-ZEB1 WT5	*SNAI1*: exons 1 and 2 *ZEB1*: exon 6	*SNAI1*: 1665 bp deletion starting at position 126 *ZEB1*: WT	*SNAI1*: 1665 bp deletion starting at position 126 *ZEB1*: WT	*SNAI1*: 5′-end of ENSE00001172559 *ZEB1*: 5′-end of ENSE00003701577
SNAI1-ZEB1 DKO59	*SNAI1*: exons 1 and 2 *ZEB1*: exon 6	*SNAI1*: 1665 bp deletion starting at position 126 *ZEB1*: 414 bp deletion starting at position 15	*SNAI1*: 1665 bp deletion starting at position 126 *ZEB1*: 414 bp deletion starting at position 15	*SNAI1*: 5′-end of ENSE00001172559 *ZEB1*: 5′-end of ENSE00003701577
SNAI1-ZEB1 DKO79	*SNAI1*: exons 1 and 2	*SNAI1*: 1665 bp deletion starting at position 126 *ZEB1*:	*SNAI1*: 1665 bp deletion starting at position 126 *ZEB1*:	*SNAI1*: 5′-end of ENSE00001172559 *ZEB1*:

	ZEB1: exon 6	414 bp deletion starting at position 15	754 bp insertion at position 15 consisting of 340 bp unrelated sequences and inversion of *ZEB1* exon 6 positions 15-429	5′-end of ENSE00003701577
SNAI2 WT8	exon 2	WT	WT	5′-end of ENSE00001674368
SNAI2 WT9	exon 2	WT	WT	5′-end of ENSE00001674368
SNAI2 WT10	exon 2	WT	WT	5′-end of ENSE00001674368
SNAI2 KO26	exon 2	526 bp deletion starting at position 15	526 bp deletion starting at position 15	5′-end of ENSE00001674368
SNAI2 KO27	exon 2	895 bp deletion starting at position 15	756 bp insertion at position 15 consisting of 230 bp unrelated sequences and inversion of *SNAI2* exon 2 positions 15-541	5′-end of ENSE00001674368
SNAI2 KO35	exon 2	526 bp deletion starting at position 15	526 bp deletion starting at position 15	5′-end of ENSE00001674368
JUN WT2	exon 1	WT	WT	5′-end of ENSE00001454665
JUN WT4	exon 1	WT	WT	5′-end of ENSE00001454665
JUN WT10	exon 1	WT	WT	5′-end of ENSE00001454665
JUN KO1	exon 1	1021 bp deletion starting at position 963	1021 bp deletion starting at position 963	5′-end of ENSE00001454665
JUN KO28	exon 1	1021 bp deletion starting at position 963	1022 bp deletion starting at position 962	5′-end of ENSE00001454665
JUN KO28	exon 1	1021 bp deletion starting at position 963	1022 bp deletion starting at position 962	5′-end of ENSE00001454665
JUN KO73	exon 1	1021 bp deletion starting at position 963	1022 bp deletion starting at position 962	5′-end of ENSE00001454665
JUNB WT2	exon 1	WT	WT	5′-end of ENSE00001129119
JUNB WT18	exon 1	WT	WT	5′-end of ENSE00001129119
JUNB WT27	exon 1	WT	WT	5′-end of ENSE00001129119

JUNB mut1	exon 1	181 bp deletion starting at position 444	181 bp deletion starting at position 444	5′-end of ENSE00001129119
JUNB mut22	exon 1	1 bp deletion at position 444	1 bp deletions at positions 444 and 623	5′-end of ENSE00001129119
JUNB mut65	exon 1	181 bp deletion starting at position 444	181 bp deletion starting at position 444	5′-end of ENSE00001129119
**A549 cells**				
**Clone name**	**sgRNA target(s)**	**Allele 1**	**Allele 2**	**Reference for genomic coordinates**
JUNB WT15	exon 1	WT	WT	5′-end of ENSE00001129119
JUNB WT36	exon 1	WT	WT	5′-end of ENSE00001129119
JUNB mut32	exon 1	181 bp deletion starting at position 444	181 bp deletion starting at position 444	5′-end of ENSE00001129119
JUNB mut42	exon 1	181 bp deletion starting at position 444	184 bp deletion starting at position 444	5′-end of ENSE00001129119

Table S5.: List of sgRNA target sequences used in CRISRP/Cas9 experiments

sgRNAs target sequences excluding PAM (5´ - 3´)	
SNAI1 sgRNA-1	AGAGCGCGGCATAGTGGTCG
SNAI1 sgRNA-2	CGAGTCTTCTAACTTCTAGC
ZEB1 sgRNA-1	AGAGACATGTGACGCAGTCT
ZEB1 sgRNA-2	TTGCACAATTAGCAGGGGAA
SNAI2 sgRNA-1	GTAACTCTCATAGAGATACG
SNAI2 sgRNA-2	GGACACATTAGAACTCACAC
JUN sgRNA-1	ACTAACCTCACGTGAAGTGA
JUN sgRNA-2	TTTTGAAGAGAGACCGTCGG
JUNB sgRNA-1	CCGGAGTCTCAAAGCGCCTG
JUNB sgRNA-2	CAGTACTTTTACCCCCGCGG
